# Supplementary material for: Copper-catalyzed asymmetric C(sp3)-H cyanoalkylation of glycine derivatives and peptides
Source: Nat Commun. 2023 Jun 6;14:3295. doi: 10.1038/s41467-023-38871-1 (PMC10244411; doi:10.1038/s41467-023-38871-1)
Supplement: Supplementary file 1 — Supplementary Information [file 41467_2023_38871_MOESM1_ESM.pdf]

Supplementary Information

Copper-catalyzed asymmetric C(sp^3)-H cyanoalkylation of glycine derivatives and peptides

Rupeng Qi†, Qiao Chen†, Liangyu Liu†, Zijian Ma, Da Pan, Hongying Wang, Zhixuan Li, Chao Wang* and Zhaoqing Xu*

†These authors contributed equally to this work.

*Corresponding authors: wangchao@lzu.edu.cn   zqxu@lzu.edu.cn

Table of Contents

1 Supplementary Methods 2

1.1 General information 2

1.2 Substrates synthesis 2

1.3 General procedure of cyanoalkylation 20

1.4 Characterization of products 27

2 Supplementary Discussion 53

2.1 Synthetic applications 53

2.2 The mechanistic studies 58

2.3 X-ray crystallography 67

2.4 NMR spectra 60

3 Supplementary References 105

1 Supplementary Methods

1.1 General information

All commercially available reagents were used without further purification unless otherwise stated. All solvents were purified and dried according to standard methods prior to use. NMR spectra were recorded on a Bruker 300 M instrument spectrometer in $CDCl_3$ using tetramethylsilane (TMS) as internal standard unless otherwise stated. Data for ^1H NMR are recorded as follows: chemical shift (δ, ppm), multiplicity (s = singlet, d = doublet, t = triplet, m = multiplet, q = quartet, dd = doublet of doublets, brs = broad signal, coupling constant (s) in Hz, integration). Data for ^{13}C NMR are reported in terms of chemical shift (δ, ppm). Reactions were monitored by thin layer chromatography (TLC) and column chromatography purifications were carried out using silica gel. Melting points were measured on a SCW X-4 and values are uncorrected. All new compounds were further characterized by high resolution mass spectra (HRMS, ESI source). HPLC was performed on Waters 1525 Binary HPLC, using CHIRALPAK IA and IC chiral column, eluted with a mixture of hexane and ethanol.

1.2 Substrates synthesis

1.2.1 Synthesis of glycine derivatives 1a, 1b and 1e

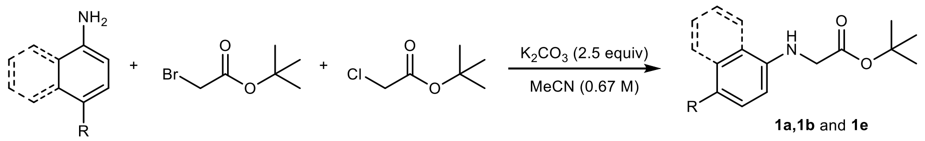

A mixture of substituted amine (20.0 mmol, 1.0 equiv), *tert*-butyl bromoacetate (16.0 mmol, 0.8 equiv), *tert*-butyl chloroacetate (14.0 mmol, 0.7 equiv) and anhydrous sodium carbonate (50.0 mmol, 2.5 equiv) in 30 mL of acetonitrile was refluxed in an oil bath for 10-16 h. After completion, filtered and concentrated under reduced pressure to afford crude residue. The crude residue was purified by silica gel column chromatography using petroleum ether/ethyl acetate as a solvent system to afford the

desired product (**1a**, **1b**, or **1e**, 70-85% yield).

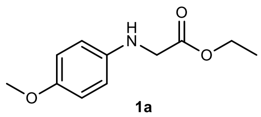

1a

ethyl (4-methoxyphenyl)glycinate (1a): ^{1}H NMR (300 MHz, $CDCl_3$) δ 6.77 (d, *J* = 9.0 Hz, 2H), 6.55 (d, *J* = 8.9 Hz, 2H), 4.20 (q, *J* = 7.1 Hz, 2H), 4.02 (s, 1H), 3.82 (s, 2H), 3.71 (s, 3H), 1.26 (t, *J* = 7.2 Hz, 3H). **^{13}C NMR** (75 MHz, $CDCl_3$) δ 171.22, 152.33, 141.12, 114.62, 114.10, 60.93, 55.42, 46.51, 13.96. **HRMS (ESI):** m/z $[M+H]^+$ calcd for $C_{11}H_{16}NO_3$: 210.1125, found: 210.1122.

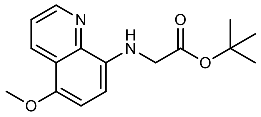

1b

***tert*-butyl (5-methoxyquinolin-8-yl)glycinate (1b): ^{1}H NMR** (300 MHz, $CDCl_3$) δ 8.79 (d, *J* = 4.4 Hz, 1H), 8.49 (d, *J* = 8.4 Hz, 1H), 7.39 (dd, *J* = 4.3 Hz, 8.5 Hz, 1H), 6.75 (d, *J* = 8.2 Hz, 1H), 6.48 (d, *J* = 8.2 Hz, 1H), 3.98 (s, 2H), 3.92 (s, 3H), 1.48 (s, 9H). **^{13}C NMR** (75 MHz, $CDCl_3$) δ 170.35, 147.88, 146.15, 138.91, 138.25, 130.66, 121.12, 120.70, 105.25, 104.41, 81.61, 55.85, 46.78, 28.07. **HRMS (ESI):** m/z $[M+H]^+$ calcd for $C_{16}H_{21}N_2O_3$: 289.1547, found: 289.1545.

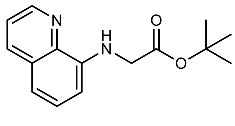

1e

***tert*-butyl quinolin-8-ylglycinate (1e): ^{1}H NMR** (300 MHz, $CDCl_3$) δ 8.73 (dd, *J* = 1.4 Hz, 5.4 Hz, 1H), 8.01 (dd, *J* = 1.7 Hz, 8.3 Hz, 1H), 7.41 – 7.27 (m, 2H), 7.06 (dd, *J* = 1.4 Hz, 8.1 Hz, 1H), 6.54 (dd, *J* = 1.4 Hz, 7.6 Hz, 1H), 4.00 (d, *J* = 1.4 Hz, 2H), 1.48 (s, 9H). **^{13}C NMR** (75 MHz, $CDCl_3$) δ 169.85, 147.07, 143.82, 138.20, 135.70, 128.43, 127.38, 121.36, 114.67, 104.78, 81.69, 46.01, 27.96. **HRMS (ESI):** m/z $[M+H]^+$ calcd for $C_{15}H_{19}N_2O_2$: 259.1441, found: 259.1444.

1.2.2 Synthesis of glycine derivatives 1c

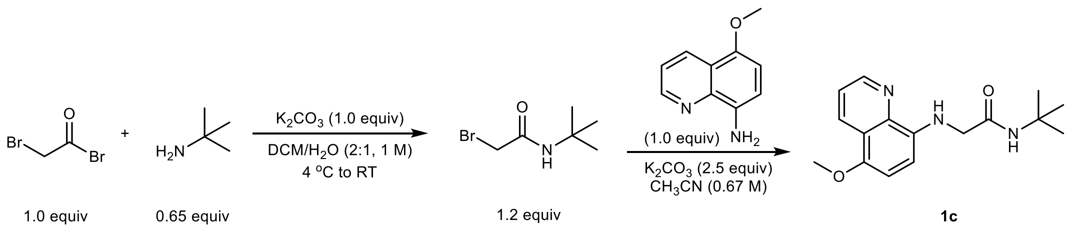

1c

A mixture of 2-bromoacetyl bromide (10.0 mmol, 1.0 equiv) in CH_2Cl_2 (3 mL) and K_2CO_3 (10.0 mmol, 1.0 equiv) in H_2O (3 mL) at 4 °C. *tert*-Butylamine (6.5 mmol, 0.65 equiv) is dissolved in CH_2Cl_2 (3 mL) and added dropwise to the above system. The mixture was then allowed to warm up to room temperature and stirred for 12 h. Then the organic layer was separated and the aqueous layer was extracted with CH_2Cl_2 (3×5 mL). Then organic layers were combined and dried over Na_2SO_4 and CH_2Cl_2 was removed in vacuo. Subsequently, CH_3CN (7.5 mL), 5-methoxyquinolin-8-amine (5.0 mmol, 1.0 equiv), and K_2CO_3 (12.5 mmol, 2.5 equiv) were added to the residue. The resulting mixture was refluxed for 12 h, and then filtered. The solvent of the filtrate was removed in vacuo. The crude residue was purified by silica gel column chromatography using petroleum ether/ethyl acetate as a solvent system to afford the desired product (**1c**, 70% yield). ***N*-(*tert*-butyl)-2-((5-methoxyquinolin-8-yl)amino)acetamide (1c):** **^{1}H NMR** (300 MHz, $CDCl_3$) δ 8.78 (d, J = 4.3 Hz, 1H), 8.53 (d, J = 8.3 Hz, 1H), 7.54 – 7.34 (m, 1H), 6.84 (brs, 1H), 6.74 (d, J = 8.2 Hz, 1H), 6.55 (d, J = 8.2 Hz, 1H), 6.12 (brs, 1H), 3.92 (s, 3H), 3.86 (s, 2H), 1.33 (s, 9H). **^{13}C NMR** (75 MHz, $CDCl_3$) δ 169.97, 147.82, 146.81, 138.55, 137.98, 130.95, 120.81, 120.66, 105.78, 104.87, 55.59, 50.61, 50.23, 28.48. **HRMS (ESI):** m/z $[M+H]^+$ calcd for $C_{16}H_{22}N_3O_2$: 288.1710, found: 288.1707.

1.2.3 Synthesis of glycine derivatives 1d[1]

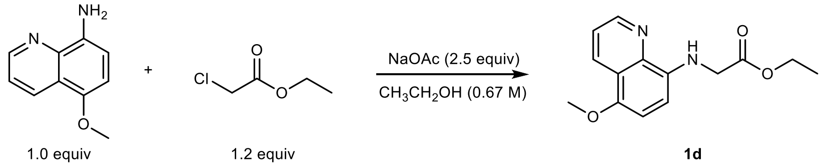

1d

A mixture of 5-methoxyquinolin-8-amine (5.0 mmol, 1.0 equiv), ethyl chloroacetate

(6.0 mmol, 1.2 equiv) and anhydrous sodium acetate (12.5 mmol, 2.5 equiv) in 7.5 mL of ethanol was refluxed in an oil bath for 6 h. The reaction mixture was left overnight at room temperature. After completion, filtered and concentrated under reduced pressure to afford crude residue. The crude residue was purified by silica gel column chromatography using petroleum ether/ethyl acetate as a solvent system to afford the desired product (**1d**, 80% yield). **ethyl (5-methoxyquinolin-8-yl)glycinate (1d): ^{1}H NMR** (300 MHz, $CDCl_3$) δ 8.78 (dd, *J* = 1.9 Hz, 4.0 Hz, 1H), 8.47 (dd, *J* = 1.9 Hz, 8.4 Hz, 1H), 7.44 – 7.33 (m, 1H), 6.71 (dd, *J* = 1.8 Hz, 8.3 Hz, 1H), 6.54 – 6.42 (m, 1H), 6.20 (brs, 1H), 4.24 (q, *J* = 6.0 Hz, 2H), 4.07 (s, 2H), 3.88 (s, 3H), 1.27 (t, *J* = 6.0 Hz, 3H). **^{13}C NMR** (75 MHz, $CDCl_3$) δ 171.09, 147.78, 146.15, 138.75, 137.95, 130.59, 121.01, 120.62, 105.05, 104.35, 60.99, 55.68, 46.01, 14.11. **HRMS (ESI):** m/z $[M+H]^+$ calcd for $C_{14}H_{17}N_2O_3$: 261.1234, found: 261.1234.

1.2.4 Synthesis of dipeptide derivatives 1f-1l[1]

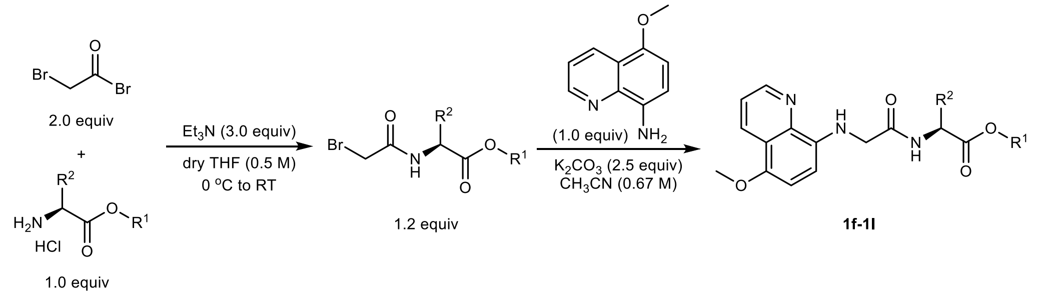

A mixture of amino acid hydrochloride (10.0 mmol, 1.0 equiv) in dry THF (20 mL) and Et_3N (30.0 mmol, 3.0 equiv) at 0 °C was added with 2-bromoacetyl bromide (20.0 mmol, 2.0 equiv) in dropwise. The mixture was then allowed to warm up to room temperature and stirred for 12 h. Then 10 mL of water was added to quench the reaction and the aqueous layer was extracted with CH_2Cl_2 (3×5 mL). Then organic layers were combined and dried over Na_2SO_4, and then, CH_2Cl_2 was removed in vacuo. Subsequently, CH_3CN (15 mL), 5-methoxyquinolin-8-amine (8.0 mmol, 1.0 equiv), and K_2CO_3 (20.0 mmol, 2.5 equiv) were added to the residue. The resulting mixture was refluxed for 12 h, and then filtered. The solvent of the filtrate was removed in vacuo. The crude residue was purified by silica gel column chromatography using petroleum

ether/ethyl acetate as a solvent system to afford the desired product (**1f-1l**, 65-80% yield).

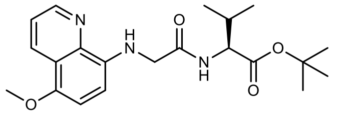

1f

***tert*-butyl (5-methoxyquinolin-8-yl)glycyl-*L*-valinate (1f): ^{1}H NMR** (300 MHz, $CDCl_3$) δ 8.80 (d, *J* = 3.9 Hz, 1H), 8.53 (d, *J* = 8.4 Hz, 1H), 7.43 (q, *J* = 4.2 Hz, 1H), 7.36 (d, *J* = 9.3 Hz, 1H), 6.75 (d, *J* = 8.4 Hz, 1H), 6.58 (d, *J* = 8.4 Hz, 1H), 6.36 (brs, 1H), 4.50 (q, *J* = 4.2 Hz, 1H), 3.99 (t, *J* = 6.0 Hz, 2H), 3.93 (s, 3H), 2.17 - 2.06 (m, 1H), 1.37 (s, 9H), 0.89 (d, *J* = 6.9 Hz, 3H), 0.77 (d, *J* = 6.9 Hz, 3H). **^{13}C NMR** (75 MHz, $CDCl_3$) δ 170.57, 170.20, 147.66, 146.61, 138.41, 137.75, 130.56, 120.62, 120.46, 105.53, 104.59, 81.34, 56.97, 55.40, 49.17, 31.09, 27.54, 18.69, 17.29. **HRMS (ESI):** m/z $[M+H]^+$ calcd for $C_{21}H_{30}N_3O_4$: 388.2231, found: 388.2237.

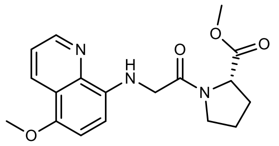

1g

methyl (5-methoxyquinolin-8-yl)glycyl-*L*-prolinate (1g): ^{1}H NMR (300 MHz, $CDCl_3$) δ 8.77 (d, *J* = 4.3 Hz, 1H), 8.46 (d, *J* = 8.4 Hz, 1H), 7.36 (dd, *J* = 4.2 Hz, 8.4Hz, 1H), 6.73 (d, *J* = 8.1 Hz, 1H), 6.47 (d, *J* = 8.1 Hz, 1H), 4.57 (dd, *J* = 3.4 Hz, 8.6 Hz, 1H), 4.03 (s, 2H), 3.90 (s, 3H), 3.72 (s, 3H), 3.68 - 3.67 (m, 1H), 3.58 - 3.52 (m, 1H), 2.28 – 1.83 (m, 4H). **^{13}C NMR** (75 MHz, $CDCl_3$) δ 172.51, 168.21, 147.68, 145.75, 138.78, 138.31, 130.33, 120.93, 120.54, 105.19, 104.03, 77.42, 77.00, 76.57, 58.81, 55.72, 52.13, 46.39, 45.78, 28.82, 24.60. **HRMS (ESI):** m/z $[M+H]^+$ calcd for $C_{18}H_{22}N_3O_4$: 344.1605, found: 344.1606.

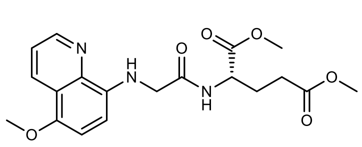

1h

dimethyl (5-methoxyquinolin-8-yl)glycyl-*L*-glutamate (1h): ^{1}H NMR (300 MHz, $CDCl_3$) δ 8.70 (d, *J* = 4.4 Hz, 1H), 8.44 (d, *J* = 8.2 Hz, 1H), 7.65 (d, *J* = 8.3 Hz, 1H),

7.45 – 7.27 (m, 1H), 6.70 (d, *J* = 8.2 Hz, 1H), 6.53 (d, *J* = 8.2 Hz, 1H), 6.36 (brs, 1H), 4.70 (q, *J* = 6.3 Hz, 1H), 3.99 (s, 2H), 3.87 (s, 3H), 3.65 (s, 3H), 3.56 (s, 3H), 2.46 – 2.10 (m, 3H), 1.98 -1.85 (m, 1H). **^{13}C NMR** (75 MHz, $CDCl_3$) δ 172.32, 171.41, 171.01, 147.39, 146.25, 138.14, 137.52, 130.25, 120.33, 120.20, 105.10, 104.42, 55.14, 51.79, 51.08, 50.72, 48.57, 29.41, 26.40. **HRMS (ESI):** m/z $[M+H]^+$ calcd for $C_{19}H_{24}N_3O_6$: 390.1660, found: 390.1660.

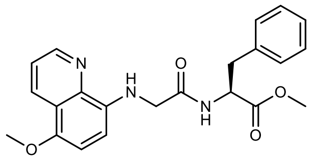

1i

methyl (5-methoxyquinolin-8-yl)glycyl-*L*-phenylalaninate (1i): ^{1}H NMR (300 MHz, $CDCl_3$) δ 8.74 (d, *J* = 4.5 Hz, 1H), 8.51 (d, *J* = 8.5 Hz, 1H), 7.44 – 7.35 (m, 1H), 7.32 (d, *J* = 8.4 Hz, 1H), 7.08 - 7.03 (m, 1H), 6.97 (t, *J* = 7.3 Hz, 2H), 6.88 (d, *J* = 7.4 Hz, 2H), 6.66 (d, *J* = 8.3 Hz, 1H), 6.40 (d, *J* = 8.3 Hz, 1H), 6.26 (brs, 1H), 4.93 (q, *J* = 6.8 Hz, 1H), 4.09 – 3.78 (m, 5H), 3.65 (s, 3H), 3.02 (qd, *J* = 6.3 Hz, 13.9 Hz, 2H). **^{13}C NMR** (75 MHz, $CDCl_3$) δ 171.46, 170.76, 147.75, 146.70, 138.46, 137.54, 135.46, 130.74, 128.80, 128.16, 126.66, 120.75, 120.61, 105.57, 104.80, 55.56, 52.39, 52.02, 48.90, 37.56. **HRMS (ESI):** m/z $[M+H]^+$ calcd for $C_{22}H_{24}N_3O_4$: 394.1761, found: 394.1760.

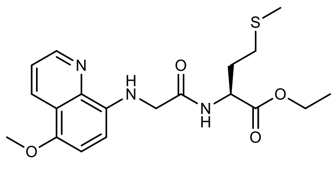

1j

ethyl (5-methoxyquinolin-8-yl)glycyl-*L*-methioninate (1j): ^{1}H NMR (300 MHz, $CDCl_3$) δ 8.83 – 8.70 (m, 1H), 8.49 (d, *J* = 9.3 Hz, 1H), 7.54 (d, *J* = 8.6 Hz, 1H), 7.42 - 7.35 (m, 1H), 6.72 (d, *J* = 10.9 Hz, 1H), 6.55 (d, *J* = 8.3 Hz, 1H), 6.38 (brs, 1H), 4.78 (q, *J* = 6.8 Hz, 1H), 4.13 (q, *J* = 7.2 Hz, 2H), 3.99 (s, 2H), 3.91 (s, 3H), 2.40 (t, *J* = 7.6 Hz, 2H), 2.19 - 2.10 (m, 1H), 1.97 - 1.84 (m, 4H), 1.20 (t, *J* = 7.1 Hz, 3H). **^{13}C NMR (75 MHz, $CDCl_3$)** δ 171.21, 170.93, 147.75, 146.74, 138.44, 137.67, 130.70, 120.70, 120.57, 105.49, 104.62, 61.19, 55.50, 50.90, 49.06, 31.25, 29.65, 15.03, 13.81. **HRMS**

(ESI): m/z [M+H]$^+$ calcd for $C_{19}H_{26}N_3O_4S$: 392.1639, found: 392.1643.

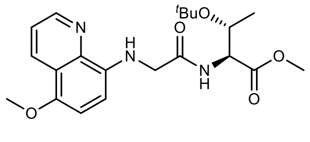

1k

methyl *O*-(tert-butyl)-*N*-((5-methoxyquinolin-8-yl)glycyl)-*L*-threoninate (1k): ^{1}H NMR (300 MHz, $CDCl_3$) δ 8.76 (d, *J* = 4.5 Hz, 1H), 8.48 (d, *J* = 8.1 Hz, 1H), 7.56 (d, *J* = 9.0 Hz, 1H), 7.36 (dd, *J* = 4.5 Hz, 9.0 Hz, 1H), 6.73 (d, *J* = 8.1 Hz, 1H), 6.62 (d, *J* = 8.1 Hz, 1H), 4.54 (d, *J* = 9.0 Hz, 1H), 4.16 (q, *J* = 6.4 Hz, 1H), 4.08 (s, 2H), 3.88 (s, 3H), 3.67 (s, 3H), 1.08 (d, *J* = 6.2 Hz, 3H), 0.89 (s, 9H). **^{13}C NMR** (75 MHz, $CDCl_3$) δ 171.36, 170.66, 147.49, 146.36, 138.36, 137.61, 130.43, 120.51, 120.28, 105.61, 104.68, 73.38, 66.80, 57.34, 55.35, 51.69, 48.76, 27.64, 20.55. **HRMS (ESI):** m/z [M+H]$^+$ calcd for $C_{21}H_{30}N_3O_5$: 404.2180, found: 404.2177.

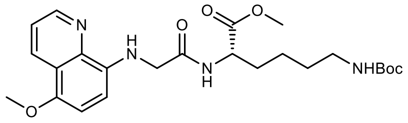

1l

methyl *N*^{6}-(tert-butoxycarbonyl)-*N*^{2}-((5-methoxyquinolin-8-yl)glycyl)-*L*-lysinate (1l): ^{1}H NMR (300 MHz, $CDCl_3$) δ 8.79 (d, *J* = 4.3 Hz, 1H), 8.53 (d, *J* = 8.4 Hz, 1H), 7.42 (dd, *J* = 5.0 Hz, 9.1 Hz, 1H), 7.37 (d, *J* = 8.4 Hz, 1H), 6.77 (d, *J* = 8.3 Hz, 1H), 6.57 (d, *J* = 8.4 Hz, 1H), 6.36 (s, 1H), 4.71 - 4.63 (m, 2H), 4.02 (s, 2H), 3.93 (s, 3H), 3.68 (s, 3H), 3.02 (q, *J* = 6.8 Hz, 2H), 1.89 - 1.77 (m, 1H), 1.66 - 1.53 (m, 1H), 1.48 - 1.36 (m, 11H), 1.26 (dd, *J* = 6.0 Hz, 15 Hz, 2H). **^{13}C NMR** (75 MHz, $CDCl_3$) δ 172.30, 171.07, 155.82, 147.98, 147.05, 138.68, 137.87, 130.97, 120.89, 120.76, 105.96, 104.79, 78.92, 55.69, 52.17, 51.44, 49.41, 40.11, 31.71, 29.20, 28.28, 22.46. **HRMS (ESI):** m/z [M+H]$^+$ calcd for $C_{24}H_{35}N_4O_6$: 475.2551, found: 475.2545.

1.2.5 Synthesis of peptide derivatives 1m-1o[2]

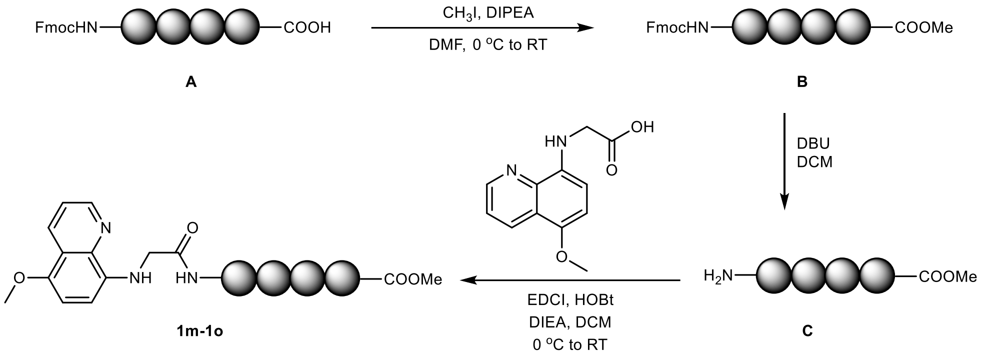

Polypeptide **A** was prepared via standard solid phase peptide synthesis procedure. To a 10 mL round bottom flask, **A** (1.0 mmol, 1.0 equiv) was dissolved by DMF (5 mL) at 0 °C. CH_3I (2.5 mmol, 2.5 equiv) and DIPEA (2.0 mmol, 2.0 equiv) were added slowly. Then, the reaction mixture was stirred at room temperature. After 12 h, the reaction mixture was slowly added to the stirred water. The undissolved residue was filtrated and dried under a vacuum to give product **B** as a white solid, which was used for the next step without further purification. DBU (3.0 mmol, 3.0 equiv) was added into the solution of **B** in CH_2Cl_2 at room temperature. The reaction was monitored by TLC. When the reaction was completed, the mixture was concentrated under vacuum to give the crude product of peptide **C**, which was used for the next step without further purification. To a 10 mL round-bottom flask, peptide **C** (1.0 mmol, 1.0 equiv), EDCl (1.2 mmol, 1.2 equiv), and HOBt (1.2 mmol, 1.2 equiv) were dissolved in 5 mL DCM at 0 °C, DIEA (3.0 mmol, 3.0 equiv) was added and stirred for 10 min. (5-methoxyquinolin-8-yl)glycine (1.2 mmol, 1.2 equiv) was added. The reaction mixture maintained the temperature for 30min and then warmed to room temperature and stirred overnight. After the reaction was completed, the reaction mixture was extracted with DCM and washed with 5% Citric acid (5 mL), saturated $NaHCO_3$ (5 mL), and brine (2 x 5 mL), the combined organic layers were dried over anhydrous Na_2SO_4 and concentrated in vacuo. The residue was purified by column chromatography and the desired product **1m-1o** was obtained.

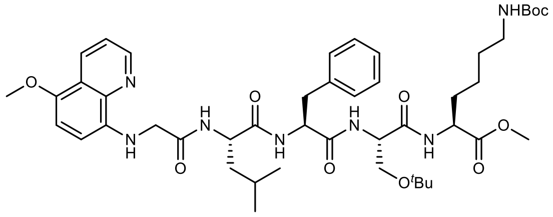

1m

methyl *N*^{6}-(tert-butoxycarbonyl)-*N*^{2}-(*O*-(*tert*-butyl)-*N*-(5-methoxyquinolin-8-yl)glycyl-*L*-leucyl-*L*-phenylalanyl-*L*-seryl)-*L*-lysinate (1m): ^{1}H NMR (300 MHz, $CDCl_3$) δ 8.82 (d, *J* = 3.9 Hz, 1H), 8.55 (d, *J* = 8.4 Hz, 1H), 7.46 (dd, *J* = 4.2 Hz, 8.6 Hz, 1H), 7.27 - 7.24 (m, 4H), 7.13 (d, *J* = 6.8 Hz, 2H), 7.05 (d, *J* = 7.4 Hz, 1H), 6.82 (brs, 2H), 6.73 (d, *J* = 8.3 Hz, 1H), 6.43 (d, *J* = 8.2 Hz, 1H), 6.25 (brs, 1H), 4.66 - 4.52 (m, 3H), 4.42 (brs, 2H), 3.93 (s, 3H), 3.91 – 3.78 (m, 3H), 3.72 (s, 3H), 3.42 - 3.34 (m, 1H), 3.23 – 2.95 (m, 4H), 1.75 - 1.63 (m, 1H), 1.46 - 1.42 (m, 11H), 1.34 (t, *J* = 7.5 Hz, 4H), 1.19 (s, 9H), 0.77 (dd, *J* = 6.0 Hz, 11.5 Hz, 6H). **^{13}C NMR** (75 MHz, $CDCl_3$) δ 172.24, 172.05, 171.81, 170.44, 169.69, 155.87, 148.08, 147.14, 138.67, 137.63, 136.41, 131.06, 129.08, 128.52, 126.88, 121.03, 120.92, 105.62, 104.89, 78.92, 73.96, 61.00, 55.75, 54.61, 52.20, 51.99, 51.36, 49.07, 40.14, 39.45, 37.45, 31.83, 28.31, 27.27, 24.36, 22.80, 22.36, 21.47. **HRMS (ESI):** m/z $[M+H]^+$ calcd for $C_{46}H_{68}N_7O_{10}$: 878.5022, found: 878.5017.

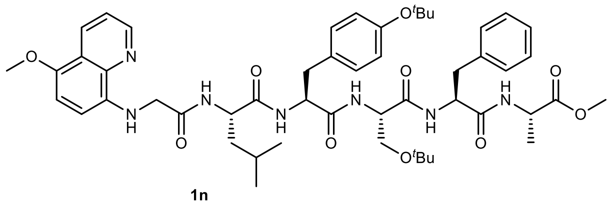

1n

methyl *N*-((*S*)-3-(4-(*tert*-butoxy)phenyl)-2-((*S*)-2-(2-((5-methoxyquinolin-8-yl)amino)acetamido)-4-methylpentanamido)propanoyl)-*O*-(*tert*-butyl)-*L*-seryl-*L*-phenylalanyl-*L*-alaninate (1n): ^{1}H NMR (300 MHz, DMSO-d_6) δ 8.82 (d, *J* = 4.3 Hz, 1H), 8.45 (d, *J* = 8.4 Hz, 1H), 7.54 (dd, *J* = 8.6, 4.2 Hz, 1H), 7.35 – 7.13 (m, 8H), 7.07 (dd, *J* = 17.9, 8.1 Hz, 3H), 6.89 - 6.83 (m, 2H), 6.77 (d, *J* = 7.9 Hz, 2H), 6.56 – 6.47 (m, 1H), 6.43 (d, *J* = 8.3 Hz, 1H), 4.68 - 4.51 (m, 3H), 4.43 – 4.21 (m, 4H), 3.87 (s, 4H), 3.61 (brs, 4H), 3.06 - 2.91 (m, 3H), 2.85 - 2.65 (m, 3H), 1.29 - 1.27 (m, 4H), 1.18 (s, 9H), 1.07 (s, 9H), 0.78 (d, *J* = 6.4 Hz, 6H).**^{13}C NMR** (75 MHz, DMSO-d_6) δ 172.76,

171.82, 171.09, 170.40, 169.56, 169.48, 153.30, 147.83, 145.02, 138.24, 138.04, 137.37, 132.41, 130.27, 129.67, 129.24, 127.99, 126.23, 123.30, 121.11, 120.32, 105.98, 104.57, 77.48, 72.93, 62.35, 55.78, 53.37, 51.91, 47.55, 41.66, 37.66, 36.83, 28.56, 28.48, 27.10, 24.05, 23.07, 21.46, 16.92. **HRMS (ESI):** m/z $[M+H]^+$ calcd for $C_{51}H_{70}N_7O_{10}$: 940.5179, found: 940.5170.

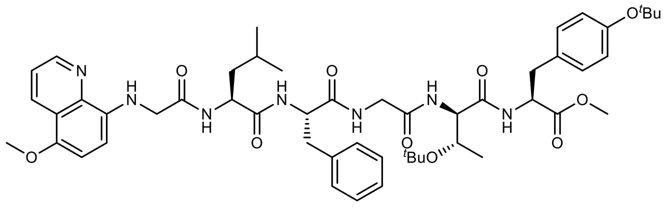

1o

methyl (2*S*,5*R*,11*S*,14*S*)-11-benzyl-2-(4-(*tert*-butoxy)benzyl)-5-((*S*)-1-(*tert*-butoxy)ethyl)-14-isobutyl-17-((5-methoxyquinolin-8-yl)amino)-4,7,10,13,16-pentaoxo-3,6,9,12,15-pentaazaheptadecanoate (1o): **^{1}H NMR** (300 MHz, $CDCl_3$) δ 8.80 (d, *J* = 3.9 Hz, 1H), 8.54 (d, *J* = 8.3 Hz, 1H), 7.61 (d, *J* = 7.8 Hz, 1H), 7.44 (dd, *J* = 4.2 Hz, 8.4 Hz, 1H), 7.20 - 7.18 (m, 4H), 7.09 - 7.05 (m, 6H), 6.95 (d, *J* = 7.8 Hz, 1H), 6.87 (d, *J* = 8.1 Hz, 2H), 6.75 (d, *J* = 8.3 Hz, 1H), 6.50 (d, *J* = 8.2 Hz, 1H), 6.29 (brs, 1H), 4.85 (q, *J* = 7.4 Hz, 1H), 4.71 (q, *J* = 7.6 Hz, 1H), 4.51 – 4.31 (m, 2H), 4.13 – 3.98 (m, 2H), 3.91 - 3.89 (m, 6H), 3.65 (s, 3H), 3.21 - 3.06 (m, 2H), 3.02 - 2.84 (m, 2H), 1.31 - 1.29 (m, 11H), 1.23 (s, 9H), 0.83 (d, *J* = 6.3 Hz, 3H), 0.75 (dd, *J* = 6.1 Hz, 9.1 Hz, 6H).**^{13}C NMR** (75 MHz, $CDCl_3$) δ 172.14, 171.95, 171.72, 171.16, 168.89, 168.21, 154.30, 148.15, 147.23, 138.74, 137.67, 136.65, 131.17, 130.73, 129.65, 129.06, 128.50, 126.81, 124.24, 121.11, 120.98, 105.74, 104.99, 78.35, 75.49, 65.95, 57.34, 55.80, 54.13, 53.74, 52.19, 51.91, 49.08, 42.89, 39.80, 37.60, 37.47, 28.73, 28.07, 24.45, 22.81, 21.48, 16.91. **HRMS (ESI):** m/z $[M+H]^+$ calcd for $C_{51}H_{70}N_7O_{10}$: 940.5179, found: 940.5164.

1.2.6 Synthesis of glycine derivatives 1p

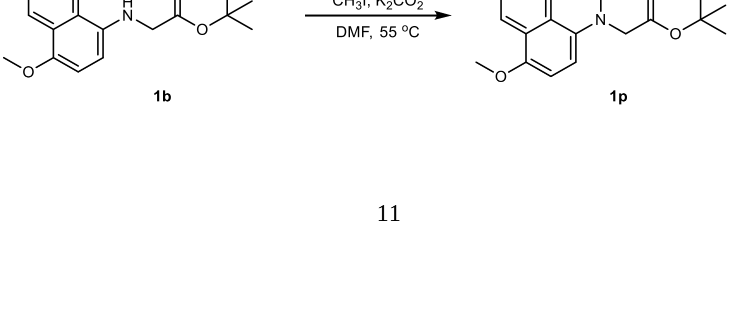

1b (1.0 mmol, 1.0 equiv), CH_3I (1.2 mmol, 1.2 equiv) and K_2CO_3 (1.3 mmol, 1.3 equiv) were dissolved by DMF (5 mL). Then, the reaction mixture was stirred at 55 °C. The reaction was monitored by TLC. When the reaction was completed (about 24 h), the reaction was quenched with water (5 mL), extracted with ethyl acetate, washed with brine, dried over anhydrous sodium sulfate, concentrated in vacuo, and purified by column chromatography (petroleum ether/ethyl acetate) to afford the product **1p** (70% yield). ***tert*-butyl *N*-(5-methoxyquinolin-8-yl)-*N*-methylglycinate (1p): ^{1}H NMR** (300 MHz, $CDCl_3$) δ 8.85 (d, *J* = 4.4 Hz, 1H), 8.52 (d, *J* = 8.5 Hz, 1H), 7.31 (dd, *J* = 4.2 Hz, 8.4 Hz, 1H), 7.14 (d, *J* = 8.3 Hz, 1H), 6.76 (d, *J* = 8.4 Hz, 1H), 4.46 (s, 2H), 3.89 (s, 3H), 3.15 (s, 3H), 1.36 (s, 9H). **^{13}C NMR** (75 MHz, $CDCl_3$) δ 170.58, 149.30, 147.86, 142.28, 140.83, 130.72, 121.23, 119.56, 116.47, 103.74, 80.16, 57.61, 55.25, 40.78, 27.82. **HRMS (ESI):** m/z $[M+H]^+$ calcd for $C_{17}H_{23}N_2O_3$: 303.1703, found: 303.1702.

1.2.7 Synthesis of glycine derivatives 1q[3]

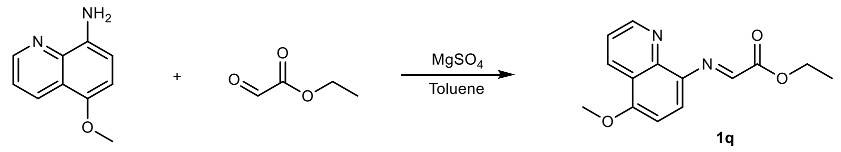

1q was prepared in a similar manner to the reported procedure[3]. To a solution of ethyl glyoxolate (50 wt.-% in toluene, 1.0 equiv) in toluene were added $MgSO_4$ (4.0 equiv) and the corresponding amine (1.0 equiv). It was stirred at room temperature for 30 min. Then the solids were filtered off and the solvent removed under reduced pressure. The product was used in the next step without further purification. **ethyl (*E*)-2-((5-methoxyquinolin-8-yl)imino)acetate (1q):** ^{1}H NMR (300 MHz, $CDCl_3$) δ 8.77 (d, *J* = 4.2 Hz, 1H), 8.50 (d, *J* = 8.4 Hz, 2H), 7.42 – 7.32 (m, 2H), 7.02 (d, *J* = 8.3 Hz, 1H), 6.69 (d, *J* = 8.2 Hz, 2H), 5.60 (d, *J* = 7.4 Hz, 1H), 4.34 (q, *J* = 6.5 Hz, 3H), 3.87 (s, 3H), 1.37 – 1.29 (m, 4H). **HRMS (ESI):** m/z $[M+H]^+$ calcd for $C_{14}H_{15}N_2O_3$: 259.1077, found: 259.1083.

1.2.8 Synthesis of cyclobutanone oxime esters 2a-2k[4]

Step 1: The ketone (5.0 mmol, 1.0 equiv) and hydroxylamine hydrochloride (7.5 mmol, 1.5 equiv) were placed in a flask equipped with stirrer. The pH of the solution was held at 7-8 by adding saturated aq. sodium carbonate. Then stirring solution at 40 °C. After completion, the mixture was extracted with DCM, the solution was dried over Na_2SO_4 and concentrated in vacuo, provide crude products which were used in next step without further purification.

Step 2: To a solution of oxime (1.0 equiv) and 3,5-dimethoxybenzoic acid (1.5 equiv) in DCM (0.2 M) was added EDCI (2.0 equiv) and DMAP (0.2 equiv). The mixture was stirred at r.t. overnight. After completion, the reaction was diluted with water and extracted with DCM (three times). The extract was washed with brine (one time) and dried over Na_2SO_4, and concentrated in vacuo. The residue was purified by flash column chromatography on silica gel with PE-EtOAc as an eluent to give oxime esters.

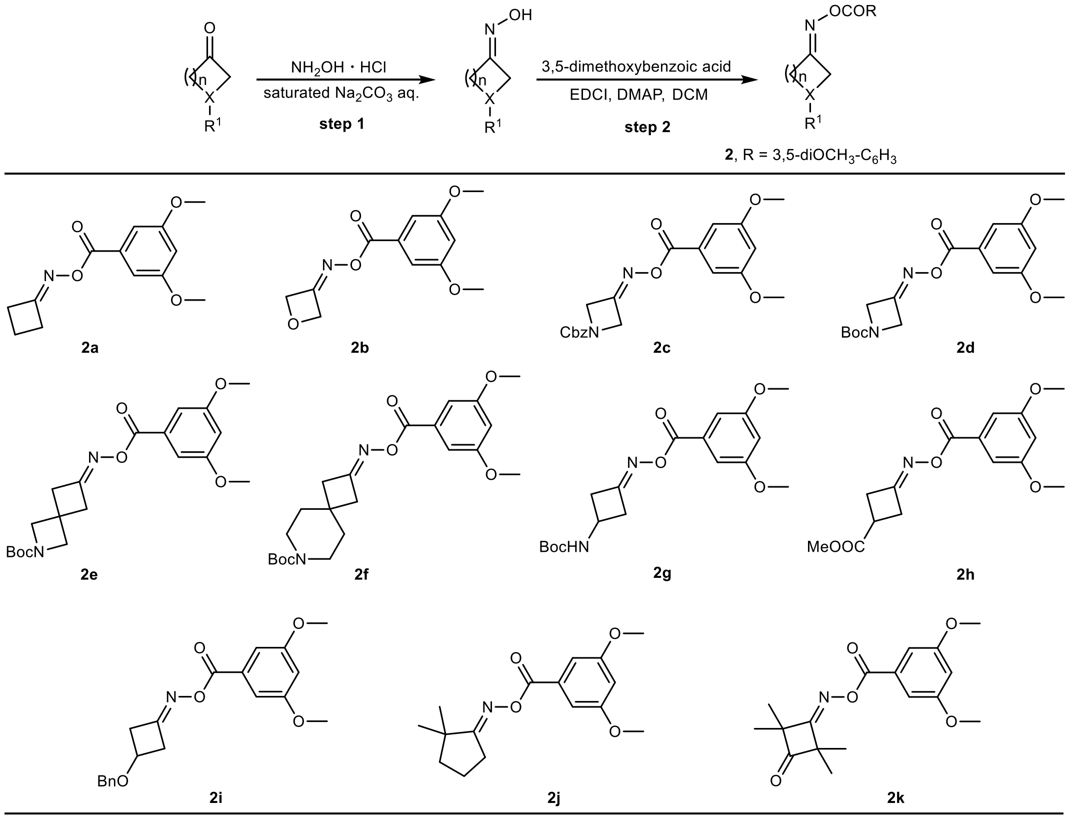

Supplementary Fig. 1. Cyclobutanone oxime esters **2a-2k**.

1.2.9 Synthesis of cyclobutanone oxime esters 2l-2o

Step 1[5]: To a suspension of (3-bromopropyl)-triphenylphosphonium bromide (1.2 equiv) in dry THF (4 mL/1 mmol of aldehyde) was added potassium *tert*-butoxide (2.4 equiv) in three portions over 30 min. The reaction mixture was heated in an oil bath to reflux for 2 h, after which the aldehyde (1.0 equiv) was added. The reaction mixture was heated in an oil bath to reflux overnight, and then cooled to room temperature and diluted with water (2 mL/1 mmol of aldehyde). The aqueous layer was extracted with pentane (3 x 1 mL/1 mmol of aldehyde). The combined organic layers were washed with brine (3 x 1 mL/1 mmol of aldehyde), dried over anhydrous $MgSO_4$, filtered, and concentrated in vacuo. The resulting oil was purified by flash column chromatography (pentane) to yield the cyclopropylidene derivative.

Step 2[5]: To the solution of cyclopropylidene derivative (1.0 equiv) in DCM (0.15 M) was added a solution of *m*-CPBA (1.0 equiv) in DCM (0.38 M) dropwise at 0 °C and stirred for 1 h. Then, the solution was diluted with a saturated solution of aqueous Na_2SO_3 (2 mL mL/1 mmol of cyclopropylidene derivative) and extracted with DCM (3 x 1 mL mL/1 mmol of cyclopropylidene derivative). The organic phase was washed successively with a saturated solution of aqueous $NaHCO_3$ (3 x 1.5 mL mL/1 mmol of cyclopropylidene derivative), and brine (1.5 mL/1 mmol of cyclopropylidene derivative), then dried over Na_2SO_4 and concentrated in vacuum. The crude material was then purified by column chromatography on silica gel with a mixture of petroleum ether and ethyl acetate (20:1) to give various cyclobutanones derivative.

Step 3[4]: The cyclobutanones derivative (1.0 equiv) and hydroxylamine hydrochloride (1.5 equiv) were placed in a flask equipped with stirrer. The pH of the solution was held at 7-8 by adding saturated aq. sodium carbonate and then stirred the solution at 40 °C. After completion, the mixture was extracted with DCM, and the solution was dried over Na_2SO_4 and concentrated in vacuo, provide crude products which were used in next step without further purification.

Step 2[4]: To a solution of oxime (1.0 equiv) and 3,5-dimethoxybenzoic acid (1.5 equiv)

in DCM (0.2 M) was added EDCI (2.0 equiv) and DMAP (0.2 equiv). The mixture was stirred at r.t. overnight. After completion, the reaction was diluted with water and extracted with DCM (three times). The extract was washed with brine (one time) and dried over Na_2SO_4 and concentrated in vacuo. The residue was purified by flash column chromatography on silica gel with PE-EtOAc as an eluent to give oxime esters.

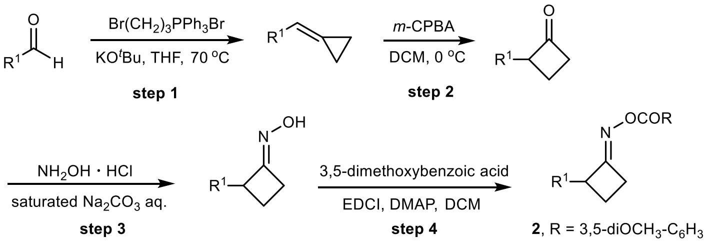

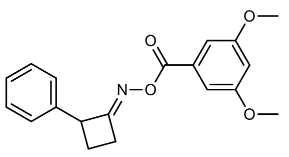

2l

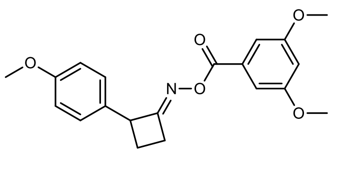

2m

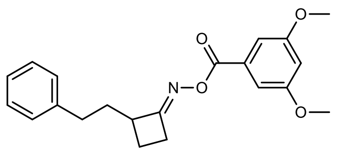

2n

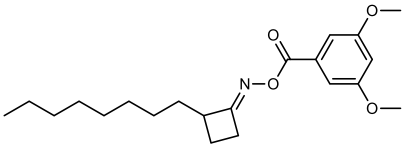

2o

Supplementary Fig. 2. Cyclobutanone oxime esters **2l-2o**.

1.2.10 Spectral data of 2a-2o

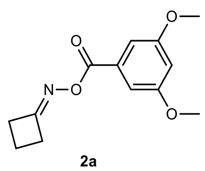

2a

cyclobutanone *O*-(3,5-dimethoxybenzoyl) oxime (2a): ^{1}H NMR (300 MHz, $CDCl_3$) δ 7.18 (s, 2H), 6.67 (s, 1H), 3.83 (s, 6H), 3.14 (t, J = 7.8 Hz, 4H), 2.17 - 2.06 (m, 2H). **^{13}C NMR** (75 MHz, $CDCl_3$) δ 169.31, 163.67, 160.55, 130.74, 107.17, 105.43, 55.47, 31.74, 14.16. **HRMS (ESI):** m/z $[M+Na]^+$ calcd for $C_{13}H_{15}NO_4Na$: 272.0893, found: 272.0896.

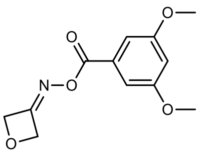

2b

oxetan-3-one *O*-(3,5-dimethoxybenzoyl) oxime (2b): ^{1}H NMR (300 MHz, $CDCl_3$) δ 7.13 (s, 2H), 6.69 (s, 1H), 5.46 (s, 4H), 3.84 (s, 6H). **^{13}C NMR** (75 MHz, $CDCl_3$) δ163.00, 160.70, 129.71, 107.37, 105.82, 78.34, 78.30, 55.56. **HRMS (ESI):** m/z $[M+H]^+$ calcd for $C_{12}H_{14}NO_5$: 252.0866, found: 252.0870.

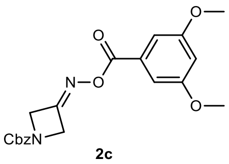

2c

benzyl 3-(((3,5-dimethoxybenzoyl)oxy)imino)azetidine-1-carboxylate (2c): ^{1}H NMR (300 MHz, $CDCl_3$) δ 7.37 (s, 5H), 7.12 (s, 2H), 6.68 (s, 1H), 5.15 (s, 2H), 4.88 (s, 4H), 3.82 (s, 6H). **^{13}C NMR** (75 MHz, $CDCl_3$) δ 162.83, 160.69, 157.89, 156.13, 135.74, 129.55, 128.52, 128.34, 128.16, 128.09, 107.30, 105.95, 71.18, 67.55, 55.52. **HRMS (ESI):** m/z $[M+H]^+$ calcd for $C_{20}H_{21}N_2O_6$: 385.1394, found: 385.1390.

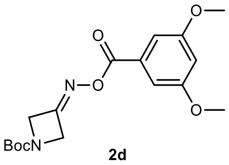

2d

***tert*-butyl 3-(((3,5-dimethoxybenzoyl)oxy)imino)azetidine-1-carboxylate (2d): ^{1}H NMR** (300 MHz, $CDCl_3$) δ 7.14 (s, 2H), 6.69 (s, 1H), 4.81 (s, 4H), 3.84 (s, 6H), 1.49 (s, 9H). **^{13}C NMR** (75 MHz, $CDCl_3$) δ 162.98, 160.70, 158.56, 155.85, 129.69, 107.34, 105.90, 81.07, 58.12, 55.55, 28.17. **HRMS (ESI):** m/z $[M+H]^+$ calcd for $C_{17}H_{23}N_2O_6$: 351.1551, found: 351.1550.

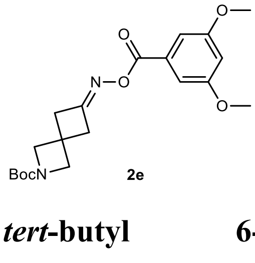

2e

***tert*-butyl 6-(((3,5-dimethoxybenzoyl)oxy)imino)-2-azaspiro[3.3]heptane-2-**

carboxylate (2e): ^{1}**H NMR** (300 MHz, $CDCl_3$) δ 7.16 (s, 2H), 6.67 (s, 1H), 4.10 - 4.07 (m, 4H), 3.83 (s, 6H), 3.33 (s, 4H), 1.45 (s, 9H). ^{13}**C NMR** (75 MHz, $CDCl_3$) δ 163.61, 163.50, 160.65, 155.92, 130.40, 107.27, 105.68, 79.83, 60.66, 55.55, 42.75, 31.70, 28.27. **HRMS (ESI):** m/z $[M+H]^+$ calcd for $C_{20}H_{27}N_2O_6$: 391.1864, found: 391.1858.

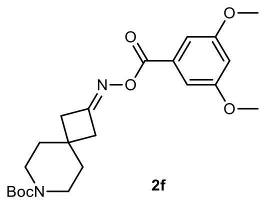

2f

***tert*-butyl 2-(((3,5-dimethoxybenzoyl)oxy)imino)-7-azaspiro[3.5]nonane-7-carboxylate (2f):** ^{1}**H NMR** (300 MHz, $CDCl_3$) δ 7.17 (s, 2H), 6.67 (s, 1H), 3.83 (s, 6H), 3.40 (s, 4H), 2.89 (s, 4H), 1.67 (s, 4H), 1.47 (s, 9H). ^{13}**C NMR** (75 MHz, $CDCl_3$) δ 165.10, 163.56, 160.58, 154.65, 130.57, 107.23, 105.51, 79.58, 55.50, 41.77, 41.73, 36.19, 33.22, 28.31. **HRMS (ESI):** m/z $[M+H]^+$ calcd for $C_{22}H_{31}N_2O_6$: 419.2177, found: 419.2183.

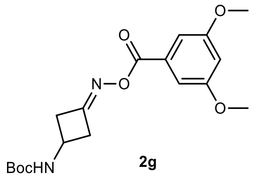

2g

***tert*-butyl (3-(((3,5-dimethoxybenzoyl)oxy)imino)cyclobutyl)carbamate (2g):** ^{1}**H NMR** (300 MHz, $CDCl_3$) δ 7.16 (s, 2H), 6.67 (s, 1H), 5.05 - 5.00 (m, 1H), 4.28 (s, 1H), 3.83 (s, 6H), 3,59 - 3.51 (m, 2H), 3.12 - 3.03 (m, 2H), 1.46 (s, 9H). ^{13}**C NMR** (75 MHz, $CDCl_3$) δ 163.68, 163.59, 160.67, 154.93, 130.49, 107.29, 105.76, 81.19, 55.58, 40.46, 40.26, 35.41. **HRMS (ESI):** m/z $[M+H]^+$ calcd for $C_{18}H_{25}N_2O_6$: 365.1707, found: 365.1704.

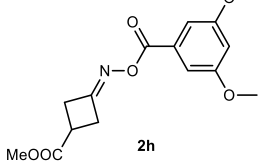

2h

methyl 3-(((3,5-dimethoxybenzoyl)oxy)imino)cyclobutane-1-carboxylate (2h): ^{1}**H NMR** (300 MHz, $CDCl_3$) δ 7.17 (s, 2H), 6.67 (s, 1H), 3.84 (s, 6H), 3.77 (s, 3H), 3.41 (d, J = 7.2 Hz, 4H), 3.30 (dd, J = 6.6 Hz, 14.4 Hz, 1H). ^{13}**C NMR** (75 MHz, $CDCl_3$) δ

173.57, 164.35, 163.45, 160.64, 130.42, 107.26, 105.72, 55.54, 52.35, 35.60, 30.82. **HRMS (ESI):** m/z $[M+H]^+$ calcd for $C_{15}H_{18}NO_6$: 308.1129, found: 308.1133.

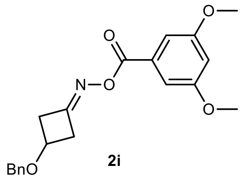

2i

3-(benzyloxy)cyclobutan-1-one *O*-(3,5-dimethoxybenzoyl) oxime (2i): ^{1}H NMR (300 MHz, $CDCl_3$) δ 7.39 -7.29 (m, 5H), 7.17 (s, 2H), 6.66 (s, 1H), 4.50 (t, J = 11.7 Hz, 2H), 4.32 - 4.23 (m, 1H), 3.82 (s, 6H), 3.44 - 3.32 (m, 2H), 3.16 - 3.05 (m, 2H). **^{13}C NMR** (75 MHz, $CDCl_3$) δ 163.54, 163.18, 160.58, 137.02, 130.53, 128.46, 127.96, 127.78, 107.21, 105.58, 71.00, 66.50, 55.49, 40.21. **HRMS (ESI):** m/z $[M+H]^+$ calcd for $C_{20}H_{22}NO_5$: 356.1492, found: 356.1497.

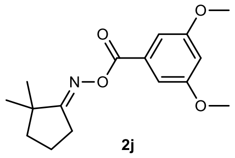

2j

(*E*)-2,2-dimethylcyclopentan-1-one *O*-(3,5-dimethoxybenzoyl) oxime (2j): ^{1}H NMR (300 MHz, $CDCl_3$) δ 7.19 (s, 2H), 6.66 (s, 1H), 3.84 (s, 6H), 2.76 (t, J = 7.2 Hz, 2H), 1.88 - 1.79 (m, 2H), 1.74 - 1.70 (m, 2H), 1.31 (s, 6H). **^{13}C NMR** (75 MHz, $CDCl_3$) δ 180.77, 163.56, 160.57, 131.26, 107.12, 105.30, 55.47, 43.20, 40.93, 28.85, 26.19, 20.58. **HRMS (ESI):** m/z $[M+H]^+$ calcd for $C_{16}H_{22}NO_4$: 292.1543, found: 292.1546.

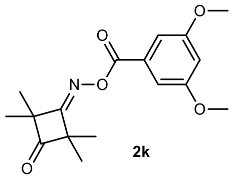

2k

3-(((3,5-dimethoxybenzoyl)oxy)imino)-2,2,4,4-tetramethylcyclobutan-1-one (2k): ^{1}H NMR (300 MHz, $CDCl_3$) δ 7.17 (s, 2H), 6.71 - 6.69 (m, 1H), 3.85 (s, 6H), 1.58 (s, 6H), 1.52 (s. 6H). **^{13}C NMR** (75 MHz, $CDCl_3$) δ 213.75, 174.05, 163.39, 160.77, 130.37, 107.17, 105.77, 65.50, 62.97, 55.55, 21.34, 20.26. **HRMS (ESI):** m/z $[M+H]^+$ calcd for $C_{17}H_{22}NO_5$: 320.1492, found: 320.1486.

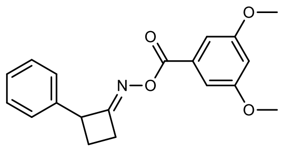

2l

(*E*)-2-phenylcyclobutan-1-one *O*-(3,5-dimethoxybenzoyl) oxime (2l): ^{1}H NMR (300 MHz, $CDCl_3$) δ 7.42 - 7.33 (m, 4H), 7.28 - 7.23 (m, 1H), 7.19 (s, 2H), 6.67 (s, 1H), 4.68 (t, *J* = 8.7 Hz, 1H), 3.83 (s, 6H), 3.29 - 3.09 (m, 2H), 2.75 - 2.56 (m, 1H), 2.33 - 2.21 (m, 1H). **^{13}C NMR** (75 MHz, $CDCl_3$) δ 170.99, 163.57, 160.50, 138.55, 130.63, 128.47, 126.98, 126.91, 107.12, 105.41, 55.40, 49.41, 29.41, 23.14. **HRMS (ESI):** m/z $[M+H]^+$ calcd for $C_{19}H_{20}NO_4$: 326.1387, found: 326.1385.

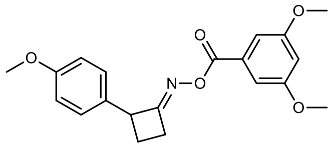

2m

(*E*)-2-(4-methoxyphenyl)cyclobutan-1-one *O*-(3,5-dimethoxybenzoyl) oxime (2m): ^{1}H NMR (300 MHz, $CDCl_3$) δ 7.53 (d, *J* = 8.4 Hz, 2H), 7.14 (s, 2H), 6.97 (d, *J* = 8.4 Hz, 2H), 6.65 (s, 1H), 3.87 - 3.80 (m, 10H), 3.41 - 3.29 (m, 1H), 3.01 - 2.83 (m, 3H). **^{13}C NMR (75 MHz, $CDCl_3$)** δ 165.51, 160.63, 160.21, 130.85, 128.41, 127.68, 126.36, 114.33, 107.53, 106.06, 93.48, 55.57, 55.33, 40.74, 25.01. **HRMS (ESI):** m/z $[M+H]^+$ calcd for $C_{20}H_{22}NO_5$: 356.1492, found: 356.1488.

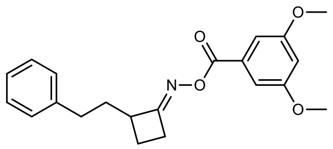

2n

(*E*)-2-phenethylcyclobutan-1-one *O*-(3,5-dimethoxybenzoyl) oxime (2n): ^{1}H NMR (300 MHz, $CDCl_3$) δ 7.31 - 7.19 (m, 5H), 7.16 (s, 2H), 6.65 (s, 1H), 4.25 (dd, *J* = 2.7 Hz, 10.2 Hz, 1H),3.82 (s, 6H), 3.03 - 2.94 (m, 1H), 2.79 - 2.69 (m, 1H), 2.29 - 2.14 (m, 1H), 2.00 - 1.80 (m, 1H), 1.25 - 1.09 (m, 4H). **^{13}C NMR (75 MHz, $CDCl_3$)** δ 165.97, 160.61, 140.69, 131.78, 128.47, 128.41, 126.12, 125.96, 107.26, 105.86, 64.41, 62.91,

55.55, 36.40, 32.75, 12.73, 12.18. **HRMS (ESI):** m/z $[M+H]^+$ calcd for $C_{21}H_{24}NO_4$: 354.1700, found: 354.1700.

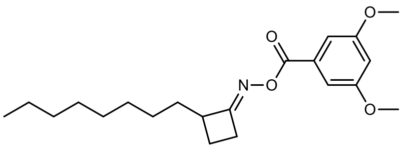

2o

(*E*)-2-octylcyclobutan-1-one *O*-(3,5-dimethoxybenzoyl) oxime (2o): ^{1}H NMR (300 MHz, $CDCl_3$) δ 7.17 (s, 2H), 6.65 (s, 1H), 4.37 (dd, *J* = 2.7 Hz, 9.6 Hz, 1H), 3.82 (s, 6H), 1.93 - 1.51 (m, 4H), 1.26 - 1.17 (m, 14H), 0.85 - 0.56 (m, 3H). **^{13}C NMR (75 MHz, $CDCl_3$)** δ. 166.38, 166.00, 160.59, 131.84, 107.21, 105.77, 64.81, 63.10, 55.48, 34.45, 31.76, 29.36, 29.15, 28.95, 26.63, 22.58, 14.03, 12.58, 11.70. **HRMS (ESI):** m/z $[M+H]^+$ calcd for $C_{21}H_{32}NO_4$: 362.2326, found: 362.2322.

1.3 General procedure of cyanoalkylation

1.3.1 Optimization of reaction conditions

Supplementary Table 1. Ligand screening.[a,b,c]

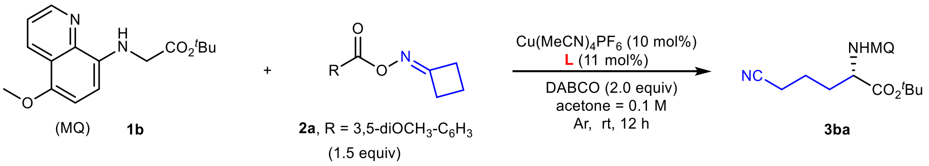

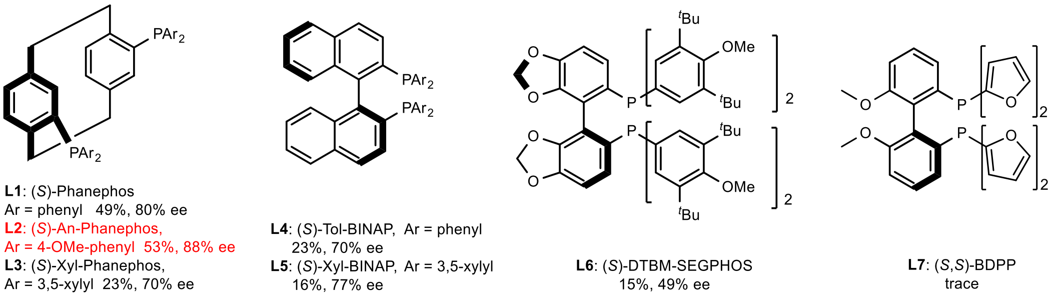

[a]0.05 mmol scale. [b]Yield was determined by ^{1}H NMR using 4-bromobenzaldehyde as an internal standard. [c]The ee was determined by HPLC on a Chiralpak IA-H column.

Supplementary Table 2. Solvent screening.

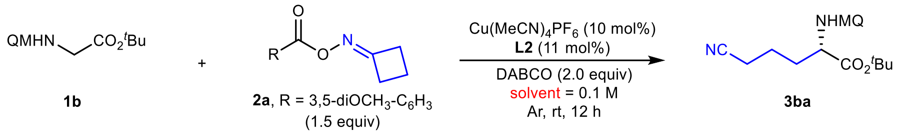

Entry[a]	solvent	Yield(%)[b]	ee(%)[c]
1	***acetone***	***53***	***88***
2	DCE	40	84
3	DMF	52	86
4	CH_3CN	19	81
5	THF	15	80
6	1,4-dioxane	10	85

[a]0.05 mmol scale. [b]Yield was determined by ^{1}H NMR using 4-bromobenzaldehyde as an internal standard. [c]The ee was determined by HPLC on a Chiralpak IA-H column.

Supplementary Table 3. Base screening.

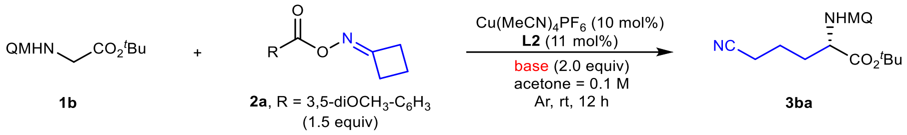

Entry[a]	base	Yield(%)[b]	ee(%)[c]
1	Et_3N	15	88
2	DBU	trace	--
3	***DABCO***	***53***	***88***
4	Cs_2CO_3	trace	--
5	K_2CO_3	10	N.D.
6	KF	trace	--

[a]0.05 mmol scale. [b]Yield was determined by ^{1}H NMR using 4-bromobenzaldehyde as an internal standard. [c]The ee was determined by HPLC on a Chiralpak IA-H column.

Supplementary Table 4. Copper salt screening.

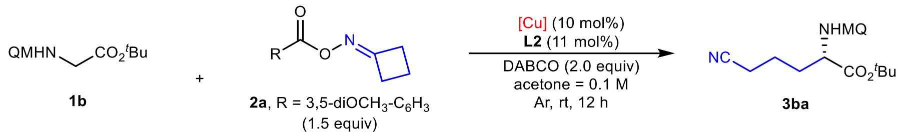

Entry[a]	Copper salt	Yield(%)[b]	ee(%)[c]
1	***$Cu(MeCN)_4PF_6$***	***53***	***88***
2	$Cu(MeCN)_4BF_4$	46	88
3	CuTc	10	60
4	$Cu(OTf)_2$	53	86
5	$Cu(OAc)_2$	30	80
6	$Cu(acac)_2$	trace	--

[a]0.05 mmol scale. [b]Yield was determined by ^{1}H NMR using 4-bromobenzaldehyde as an internal standard. [c]The ee was determined by HPLC on a Chiralpak IA-H column.

Supplementary Table 5. Oxime ester loading screening.

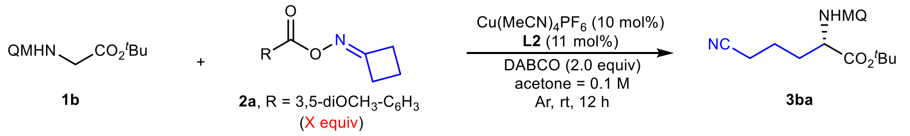

Entry[a]	X	Yield(%)[b]	ee(%)[c]
1	1.0	40	88
2	1.5	53	88
3	***2***	***76***	***88***
4	3	68	88

[a]0.05 mmol scale. [b]Yield was determined by ^{1}H NMR using 4-bromobenzaldehyde as an internal standard. [c]The ee was determined by HPLC on a Chiralpak IA-H column.

Supplementary Table 6. Other oxime esters screening.

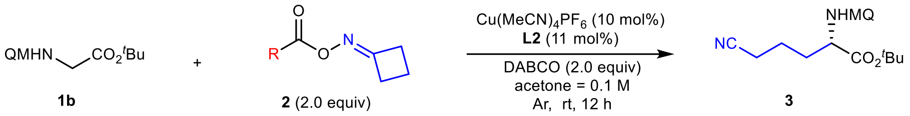

Entry[a]	R	Yield(%)[b]	ee(%)[c]
1	Ph	63	88
2	4-OCH_3-C_6H_4	56	88
3	4-CF_3-C_6H_4	61	88
4	***3,5-diOCH_3-C_6H_3***	***76***	***88***

[a]0.05 mmol scale. [b]Yield was determined by ^{1}H NMR using 4-bromobenzaldehyde as an internal standard. [c]The ee was determined by HPLC on a Chiralpak IA-H column.

Supplementary Table 7. DABCO loading screening.

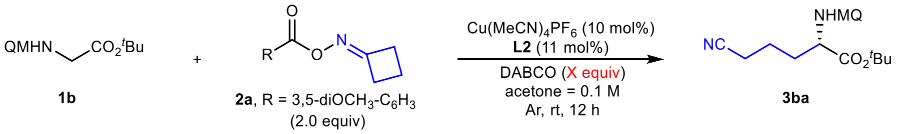

Entry[a]	X	Yield(%)[b]	ee(%)[c]
1	1.0	41	88
2	***2.0***	***76***	***88***
3	2.5	62	88

[a]0.05 mmol scale. [b] Yield was determined by ^{1}H NMR using 1,3,5-trimethoxybenzene as an internal standard. [c] The ee was determined by HPLC on a Chiralpak IA-H column.

Supplementary Table 8. Temperature screening.

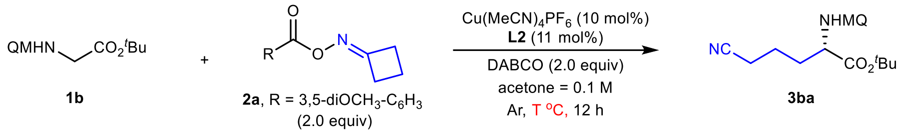

Entry[a]	T(°C)	Yield(%)[b]	ee(%)[c]
1	25	76	88
2	10	60	89
3	***0***	***50***	***92***
4	-10	30	90

[a]0.05 mmol scale. [b]Yield was determined by ^{1}H NMR using 4-bromobenzaldehyde as an internal standard. [c]The ee was determined by HPLC on a Chiralpak IA-H column.

Supplementary Table 9. Reaction time screening.

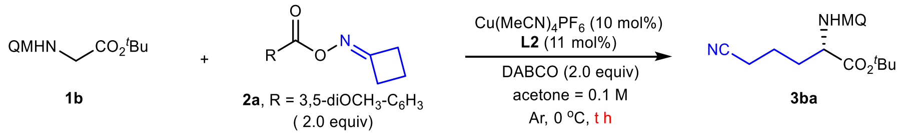

Entry[a]	t	Yield(%)[b]	ee(%)[c]
1	12	50	92
2	***18***	***67***	***92***
3	24	68	92
4	36	68	91

[a]0.1 mmol scale. [b]Yield was determined by ^{1}H NMR using 4-bromobenzaldehyde as an internal standard. [c]The ee was determined by HPLC on a Chiralpak IA-H column.

Supplementary Table 10. System concentration.

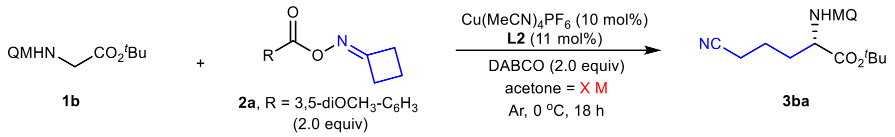

Entry[a]	X	Yield(%)[b]	ee(%)[c]
1	***0.2***	***73***	***92***
2	0.1	67	92
3	0.05	40	92
4	0.033	38	92

[a]0.1 mmol scale. [b]Yield was determined by ^{1}H NMR using 4-bromobenzaldehyde as an internal standard. [c]The ee was determined by HPLC on a Chiralpak IA-H column.

Supplementary Table 11. Copper salt and ligand loading screening.

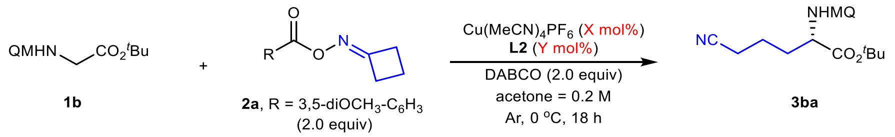

Entry[a]	X/Y	Yield(%)[b]	ee(%)[c]
1	5/6	50	92
2	10/11	73	92
3	***10/15***	***80***	***93***
4	15/17	80	92
5	20/22	78	92

[a]0.1 mmol scale. [b]Yield was determined by ^{1}H NMR using 1,3,5-trimethoxybenzene as an internal standard. [c]The ee was determined by HPLC on a Chiralpak IA-H column.

Supplementary Table 12. Control experiments under reaction conditions.

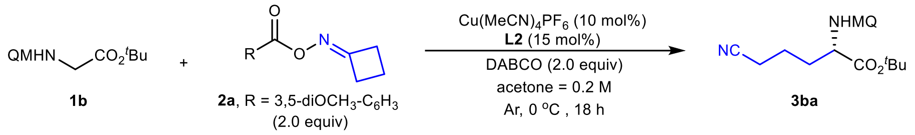

Entry[a]	Reaction conditions	Yield(%)[b]	ee(%)[c]
1	*none*	*80(75)*	*93*
2	without $Cu(MeCN)_4PF_6$	0	--
3	without DABCO	trace	--
4	without **L2**	28	0
5	White LED	80	93
6	Air	36	91

[a]0.1 mmol scale. [b]Yield was determined by ^{1}H NMR using 4-bromobenzaldehyde as an internal standard. Isolated yield in parentheses. [c]The ee was determined by HPLC on a Chiralpak IA-H column.

1.3.2 General procedure

General procedure A (standard conditions): To an oven-dried 10 mL quartz test tube with a stirring bar was added derivatives of glycine (0.1 mmol), followed by the addition of $Cu(MeCN)_4PF_6$ (0.01 mmol, 3.7 mg) and (*S*)-An-Phanephos or (*R*)-An-Phanephos (0.015 mmol, 10.5 mg). Then, the air was withdrawn and backfilled with Ar (three times). Acetone (0.25 mL) was added and the mixture was stirred at room temperature for 40 min. Subsequently, oxime esters (0.2 mmol) and DABCO (0.2 mmol, 22.4 mg) dissolved in acetone (0.25 mL) were added to the above mentioned mixed solution by syringe. Thereafter, the test tube was transferred to a low temperature device, where it was reacted for 18 h at 0 °C. Then, the reaction was quenched with water (1 mL), extracted with ethyl acetate (3 x 1.5 ml), dried over anhydrous sodium sulfate, concentrated in *vacuo*, and purified by column chromatography (hexane/ethyl acetate) to give the product (**3ba-bi, 3ca-la**).

General procedure B: To an oven-dried 10 mL quartz test tube with a stirring bar was added derivatives of peptides (0.1 mmol), followed by the addition of $Cu(MeCN)_4PF_6$

(0.01 mmol, 3.7 mg) and (*S*)-An-Phanephos (0.015 mmol, 10.5 mg). Then, the air was withdrawn and backfilled with Ar (three times). DMF (0.25 mL) was added and the mixture was stirred at room temperature for 40 min. Subsequently, oxime esters (0.2 mmol) and DABCO (0.2 mmol, 22.4 mg) dissolved in DMF (0.25 mL) were added to the above mentioned mixed solution by syringe. Thereafter, the test tube was transferred to a low temperature device, where it was reacted for 36 h at 0 °C. Then, the reaction was quenched with water (1 mL), extracted with ethyl acetate (3 x 1.5 ml), dried over anhydrous sodium sulfate, concentrated in *vacuo*, and purified by column chromatography (hexane/ethyl acetate or dichloromethane/methanol) to give the product (**3ma-oa, 3ob, 3oe** and **3of**).

1.4 Characterization of products

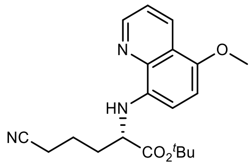

3ba

***tert*-butyl (*S*)-5-cyano-2-((5-methoxyquinolin-8-yl)amino)pentanoate (3ba)**: yellow solid. M. p. 56 - 58 ºC. 26.7 mg, 75% yield. 93% ee determined by HPLC on a Chiralpak IA-H column (hexane/EtOH = 2/1, flow rate = 0.5 mL/min, detection at 261.8 nm, t_{minor} = 11.8 min, t_{major} = 14.5 min), $[\alpha]_D^{24.8}$ 8.00 (*c* 10.00 mg/mL, DCM). **^{1}H NMR** (300 MHz, $CDCl_3$) δ 8.80 (d, *J* = 4.2 Hz, 1H), 8.50 (d, *J* = 8.4 Hz, 1H), 7.40 (q, *J* = 8.4 Hz, 1H), 6.73 (d, *J* = 8.1 Hz, 1H), 6.57 (d, *J* = 8.1 Hz, 1H), 6.22 (s, 1H), 4.16 - 4.11 (m, 1H), 3.92 (s, 3H), 2.44 (t, *J* = 7.2 Hz, 2H), 2.19 - 2.01 (m, 2H), 1.99 - 1.87 (m, 2H), 1.40 (s, 9H). **^{13}C NMR** (75 MHz, $CDCl_3$) δ 172.56, 147.95, 146.42, 139.01, 137.57, 130.77, 121.18, 120.73, 119.29, 105.24, 105.01, 81.79, 56.83, 55.83, 31.56, 27.95, 21.92, 17.07. **HRMS (ESI):** m/z $[M+H]^+$ calcd for $C_{20}H_{26}N_3O_3$: 356.1969, found: 356.1970.

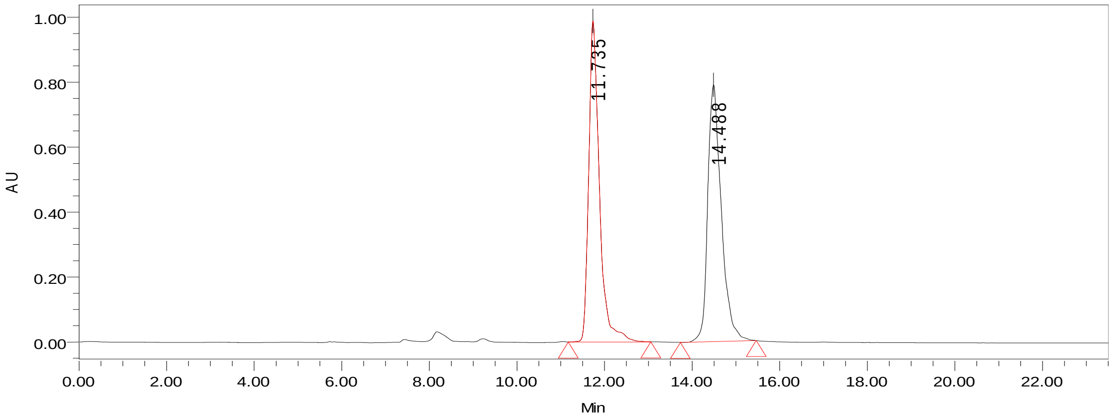

Entry	Retention time	Area	Area (%)	Height	Int type
1	11.735	16824206	49.71	988205	bb
2	14.488	17022124	50.29	790268	bb

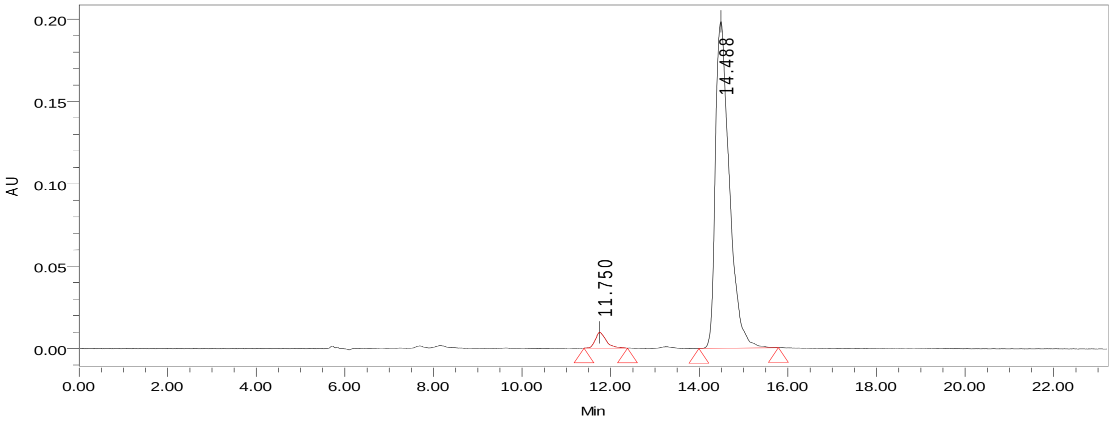

Entry	Retention time	Area	Area (%)	Height	Int type
1	11.750	161836	3.59	9612	bb
2	14.488	4349127	96.41	198468	bb

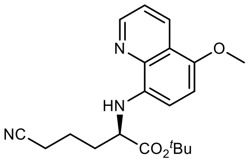

R-3ba

***tert*-butyl (*R*)-5-cyano-2-((5-methoxyquinolin-8-yl)amino)pentanoate (R-3ba):** yellow solid. M. p. 70 - 72 ºC. 24.9 mg, 70% yield. -90% ee determined by HPLC on a Chiralpak IA-H column (hexane/EtOH = 2/1, flow rate = 0.5 mL/min, detection at 261.8 nm, t_{major} = 11.7 min, t_{minor} = 14.6 min), $[\alpha]_D^{24.9}$ -1.00 (*c* 10.00 mg/mL, DCM). **^{1}H NMR** (300 MHz, $CDCl_3$) δ 8.80 (d, *J* = 4.2 Hz, 1H), 8.50 (d, *J* = 8.4 Hz, 1H), 7.40 (q, *J* = 8.4 Hz, 1H), 6.73 (d, *J* = 8.1 Hz, 1H), 6.57 (d, *J* = 8.1 Hz, 1H), 6.22 (s, 1H), 4.16 - 4.11 (m, 1H), 3.92 (s, 3H), 2.44 (t, *J* = 7.2 Hz, 2H), 2.19 - 2.01 (m, 2H), 1.99 - 1.87

(m, 2H), 1.40 (s, 9H). **^{13}C NMR** (75 MHz, $CDCl_3$) δ 172.56, 147.95, 146.42, 139.01, 137.57, 130.77, 121.18, 120.73, 119.29, 105.24, 105.01, 81.79, 56.83, 55.83, 31.56, 27.95, 21.92, 17.07. **HRMS (ESI):** m/z $[M+H]^+$ calcd for $C_{20}H_{26}N_3O_3$: 356.1969, found: 356.1971.

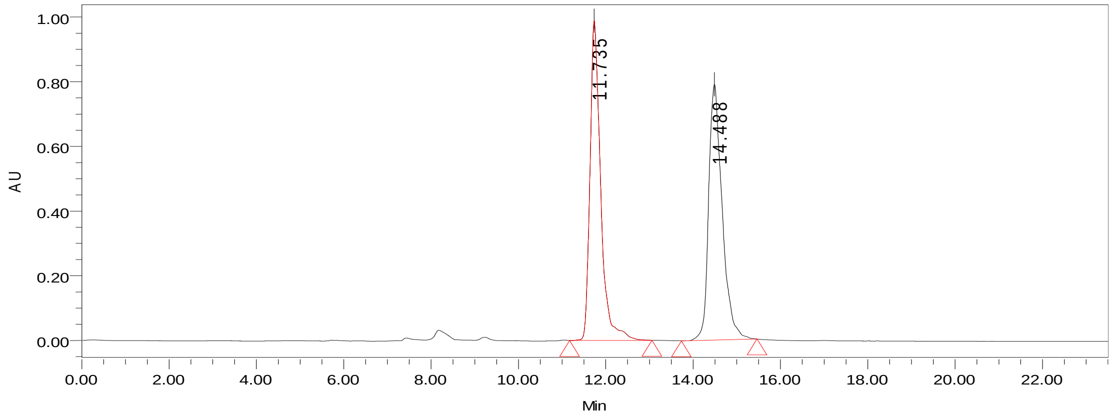

Entry	Retention time	Area	Area (%)	Height	Int type
1	11.735	16824206	49.71	988205	bb
2	14.488	17022124	50.29	790268	bb

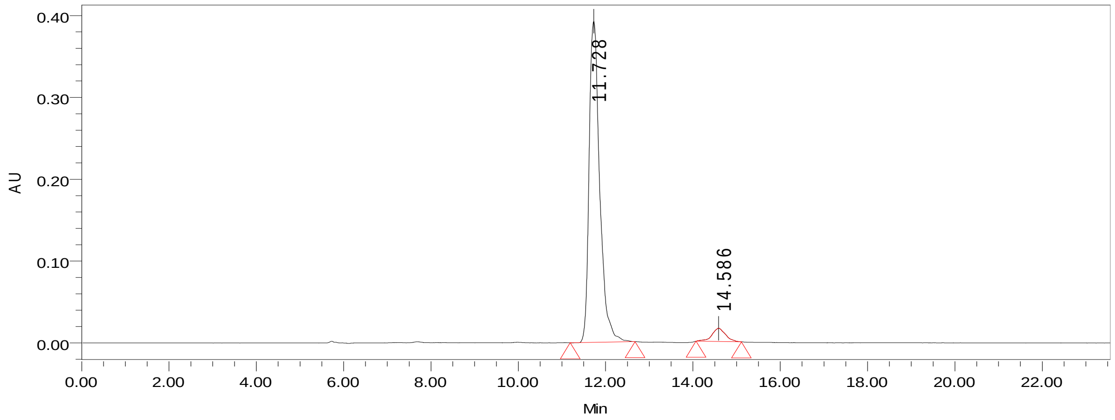

Entry	Retention time	Area	Area (%)	Height	Int type
1	11.728	6642140	95.05	392451	bb
2	14.586	345815	4.95	16218	bb

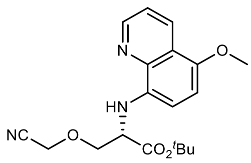

3bb

***tert*-butyl *O*-(cyanomethyl)-*N*-(5-methoxyquinolin-8-yl)-*L*-serinate (3bb):** yellow solid. M. p. 115 - 118 °C. 26.1 mg, 73% yield. 90% ee determined by HPLC on a Chiralpak IA-H column (hexane/EtOH = 2/1, flow rate = 0.5 mL/min, detection at

260.6 nm, t_{major} = 12.8 min, t_{minor} = 17.4 min), $[\alpha]_D^{25.0}$ 8.75 (*c*8.00 mg/mL, DCM). **^{1}H NMR** (300 MHz, $CDCl_3$) δ 8.80 (dd, *J* = 1.5 Hz, 4.2 Hz, 1H), 8.49 (dd, *J*= 1.5 Hz, 8.7 Hz, 1H), 7.40 (q, *J* = 4.2 Hz, 1H), 6.73 (d, *J* = 8.4 Hz, 1H), 6.58 - 6.55 (m, 1.8H) 4.46 - 4.26 (m, 3H), 4.17 - 4.12 (m, 2H), 3.93 (s, 3H), 1.46 (s, 9H). **^{13}C NMR** (75 MHz, $CDCl_3$) δ 169.90, 148.06, 146.62, 139.08, 137.25, 130.74, 121.22, 120.77, 115.60, 105.17, 104.94, 82.40, 71.77, 57.40, 56.77, 55.83, 27.96. **HRMS (ESI):** m/z $[M+H]^+$ calcd for $C_{19}H_{24}N_3O_4$: 358.1761, found: 358.1763.

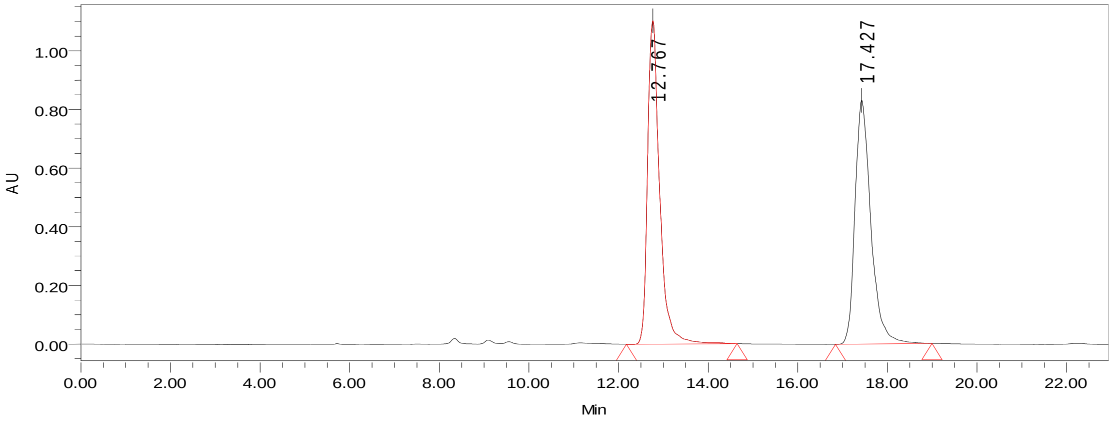

Entry	Retention time	Area	Area (%)	Height	Int type
1	12.767	20401551	50.08	1101478	bb
2	17.427	20332461	49.92	830698	bb

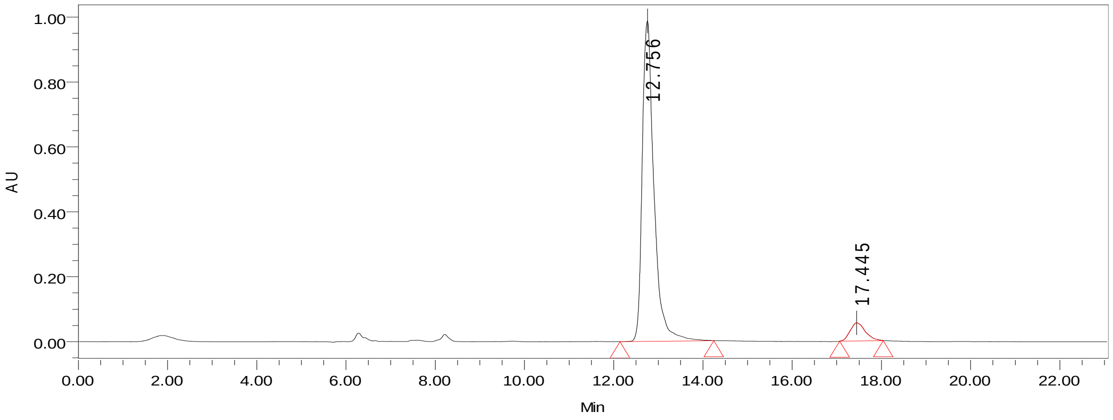

Entry	Retention time	Area	Area (%)	Height	Int type
1	12.756	18067648	94.82	987760	bb
2	17.445	987541	5.18	48965	bb

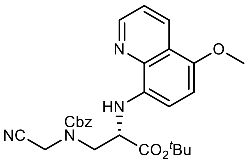

3bc

***tert*-butyl (*S*)-3-(((benzyloxy)carbonyl)(cyanomethyl)amino)-2-((5-methoxyquinolin-8-yl)amino)propanoate (3bc)**: yellow oil. 37.8 mg, 77% yield. 87% ee determined by HPLC on a Chiralpak IC-H column (hexane/EtOH = 1/2, flow rate = 0.5 mL/min, detection at 260.6 nm, t_{major} = 12.6 min, t_{minor} = 14.3 min), $[\alpha]_D^{25.1}$ -12.00 (*c* 10.00 mg/mL, DCM). **^{1}H NMR** (300 MHz, $CDCl_3$) δ 8.77 (s, 1H), 8.48 (d, *J* = 8.4 Hz, 1H), 7.44 - 7.37 (m, 6H), 6.60 - 6.41 (m, 3H), 5.26 - 5.21 (m, 2H), 4.47 - 4.30 (m, 3H), 4.04 - 3.93 (m, 1H), 3.89 (s, 3H), 3.81 - 3.74 (m, 1H), 1,40 (s, 9H). **^{13}C NMR** (75 MHz, $CDCl_3$) δ 170.59, 155.40 (154.94), 147.99, 146.62, 138.91, 137.16 (137.02), 135.51, 130.72, 128.57, 128.36, 128.29, 121.04, 120.72, 115.61 (115.56), 105.27, 104.81, 82.53, 68.56 (68.33), 57.21 (56.94), 55,70, 49.90 (49.26), 36.92 (36.71), 27.83. **HRMS (ESI):** m/z $[M+H]^+$ calcd for $C_{27}H_{31}N_4O_5$: 491.2289, found: 491.2296.

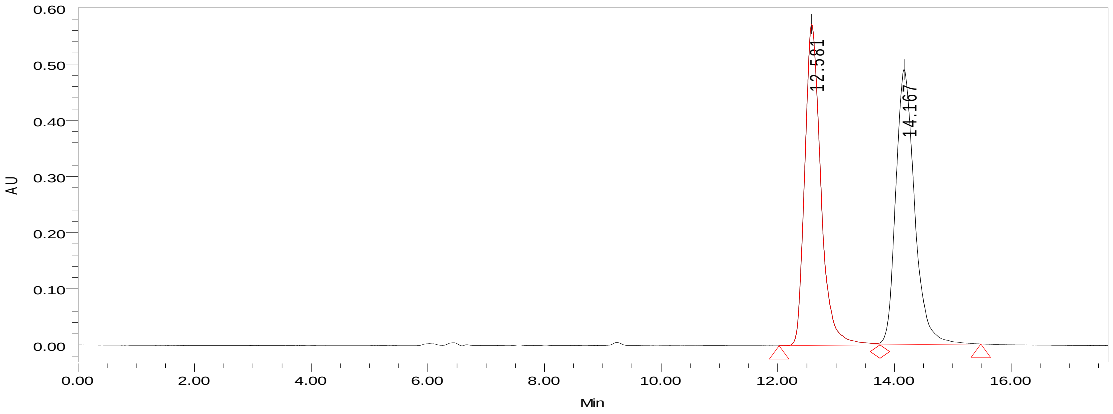

Entry	Retention time	Area	Area (%)	Height	Int type
1	12.581	11008593	49.97	572388	bv
2	14.167	11021206	50.03	489824	vb

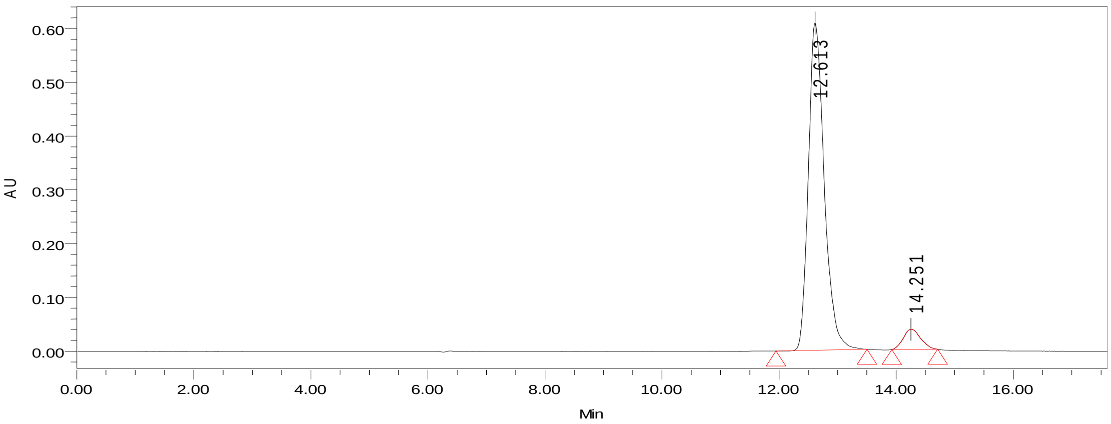

Entry	Retention time	Area	Area (%)	Height	Int type
1	12.613	11504133	93.68	608562	bb
2	14.251	776411	6.32	37400	bb

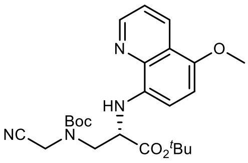

3bd

***tert*-butyl (*S*)-3-((*tert*-butoxycarbonyl)(cyanomethyl)amino)-2-((5-methoxyquinolin-8-yl)amino)propanoate (3bd):** brown oil. 36.5 mg, 80% yield. 93% ee determined by HPLC on a Chiralpak IA-H column (hexane/EtOH = 1/1, flow rate = 0.5 mL/min, detection at 260.6 nm, t_{minor} = 8.4 min, t_{major} = 8.9 min), $[\alpha]_D^{25.2}$ -12.00 (*c* 10.00 mg/mL, DCM). **^{1}H NMR** (300 MHz, $CDCl_3$) δ 8.79 (d, *J* = 3.0 Hz, 1H), 8.50 (d, *J* = 8.4 Hz, 1H), 7.40 (q, *J* = 4.2 Hz, 1H), 6.71 (d, *J* = 8.1 Hz, 1H), 6.64 - 6.57 (m, 1H), 6.45 (s, 1H), 4.44 - 4.32 (m, 3H), 3.96 - 3.87 (m, 4H), 3.74 -3.71 (m, 1H), 1.55 (d, *J* = 14.1 Hz, 9H), 1.43 (s, 9H). **^{13}C NMR** (75 MHz, $CDCl_3$) δ 170.81, 154.40 (154.15), 147.97, 146.64, 139.00, 137.48, 130.76, 121.16, 120.78, 115.93, 105.11, 104.96, 82.42 (82.18), 77.20, 57.40 (57.14), 55.83, 49.63 (49.50), 37.41 (35.95), 28.17, 27.93. **HRMS (ESI):** m/z $[M+H]^+$ calcd for $C_{24}H_{33}N_4O_5$: 457.2445, found: 457.2451.

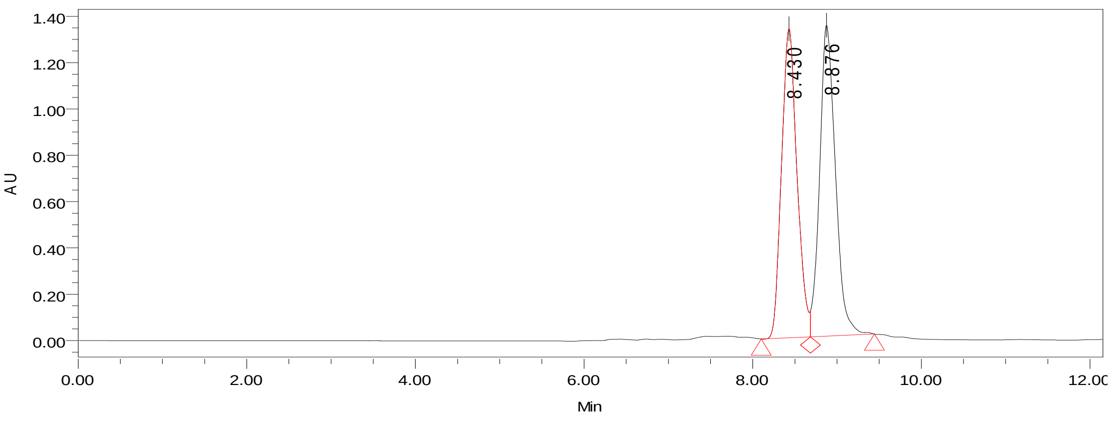

Entry	Retention time	Area	Area (%)	Height	Int type
1	8.430	17015477	49.39	1333849	bv
2	8.876	17432445	50.61	1342039	vb

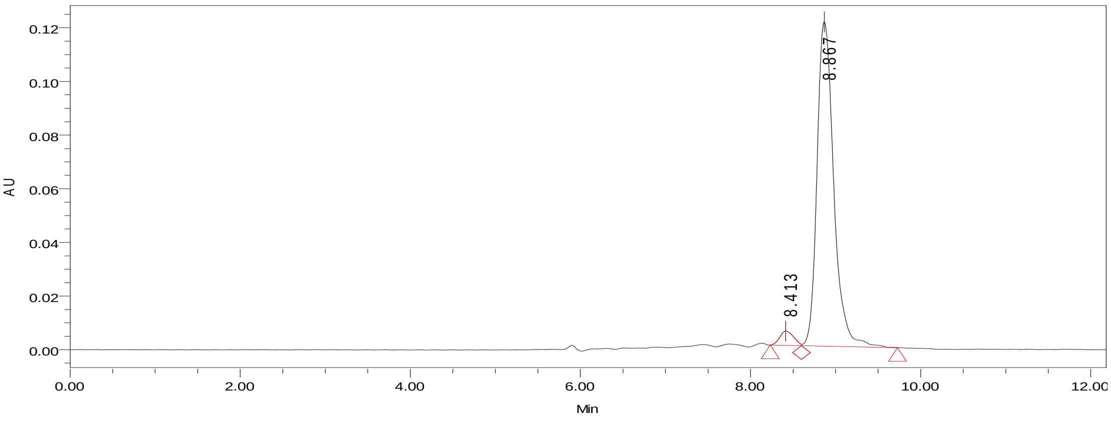

Entry	Retention time	Area	Area (%)	Height	Int type
1	8.413	60235	3.52	5313	bv
2	8.867	1649232	96.48	120840	vb

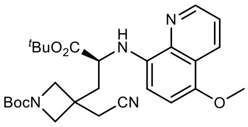

3be

***tert*-butyl (*S*)-3-(3-(*tert*-butoxy)-2-((5-methoxyquinolin-8-yl)amino)-3-oxopropyl)-3-(cyanomethyl)azetidine-1-carboxylate (3be):** yellow solid. M. p. 154 - 156 °C. 34.8 mg, 70% yield. 90% ee determined by HPLC on a Chiralpak IA-H column (hexane/EtOH = 2/1, flow rate = 0.5 mL/min, detection at 260.6 nm, t_{minor} = 10.1 min, t_{major} = 11.4 min), $[\alpha]_D^{25.3}$ -11.00 (*c* 10.00 mg/mL, DCM). **^{1}H NMR** (300 MHz, $CDCl_3$) δ 8.79 (d, *J* = 3.9 Hz, 1H), 8.50 (d, *J* = 8.4 Hz, 1H), 7.40 (dd, *J* = 4.2 Hz, 8.1 Hz ,1H), 6.72 (d, *J* = 8.4 Hz, 1H), 6.62 (d, *J* = 8.4 Hz, 1H), 6.10 (d, *J* = 10.8 Hz, 1H), 4.17-4.09

(m,1H), 3.99 - 3.90 (m, 5H), 3.79 (d, *J* = 8.7 Hz, 2H), 2.98 - 2.82 (m, 2H), 2.39 -2.37 (m, 2H), 1.44 (s, 9H), 1.32 (s, 9H). **^{13}C NMR** (75 MHz, $CDCl_3$) δ 172.36, 156.10, 148.13, 147.01, 139.25, 137.20, 130.77, 121.14, 120.80, 117.15, 106.16, 104.67, 82.21, 80.04, 77.20, 55.78, 55.14, 38.96, 34.87, 28.29, 27.81, 25.57. **HRMS (ESI):** m/z $[M+H]^+$ calcd for $C_{27}H_{37}N_4O_5$: 497.2758, found: 497.2763.

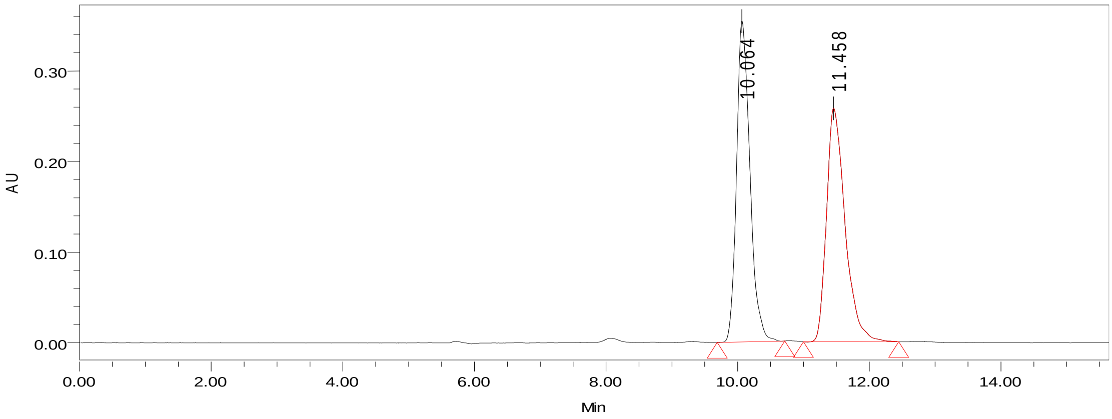

Entry	Retention time	Area	Area (%)	Height	Int type
1	10.064	5027523	49.85	354010	bb
2	11.458	5057100	50.15	257640	bb

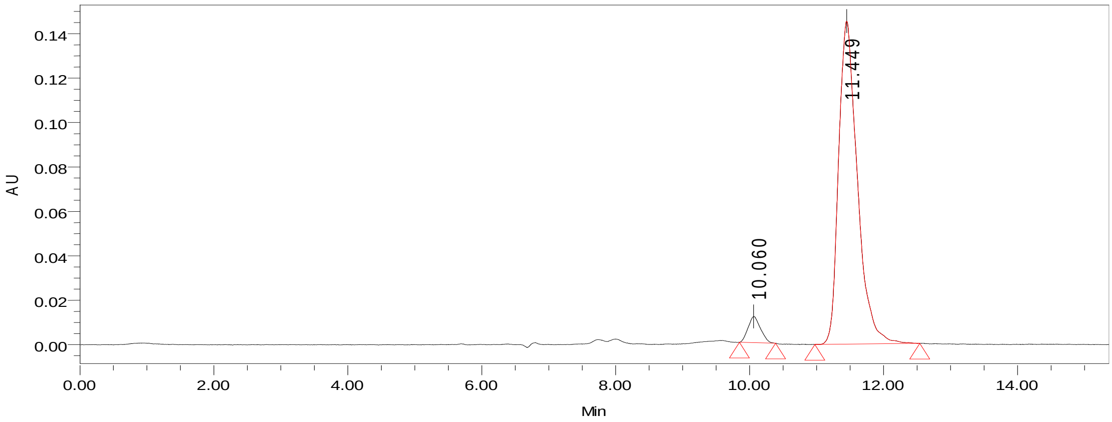

Entry	Retention time	Area	Area (%)	Height	Int type
1	10.060	152214	5.10	11824	bb
2	11.449	2832147	94.90	145360	bb

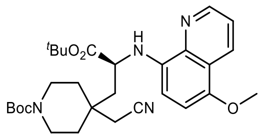

3bf

***tert*-butyl (*S*)-4-(3-(*tert*-butoxy)-2-((5-methoxyquinolin-8-yl)amino)-3-oxopropyl)-4-(cyanomethyl)piperidine-1-carboxylate (3bf):** yellow solid. M. p. 173 - 174 °C. 39.9 mg, 76% yield. 90% ee determined by HPLC on a Chiralpak IA-H column (hexane/EtOH = 2/1, flow rate = 0.5 mL/min, detection at 260.6 nm, t_{minor} = 11.0 min, t_{major} = 12.8 min), $[\alpha]_D^{25.5}$ -11.24 (*c* 8.90 mg/mL, DCM). **^{1}H NMR** (300 MHz, $CDCl_3$) δ 8.80 (d, *J* = 4.2 Hz, 1H), 8.50 (d, *J* = 8.4 Hz, 1H), 8.74 (q, *J* = 4.2 Hz, 1H), 6.72 (d, 8.4 Hz, 1H), 6.61 (d, *J* = 8.4 Hz, 1H), 6.10 (d, *J* = 11.1 Hz, 1H), 4.14 (t, *J* = 7.8 Hz, 1H), 3.92 (s, 3H), 3.47 (s, 4H), 2.75 - 2.56 (m, 2H), 2.09 - 2.02 (m, 2H), 1.75 - 1.63 (m, 4H), 1.46 (s, 9H), 1.32 (s, 9H). **^{13}C NMR** (75 MHz, $CDCl_3$) δ 173.05, 154.66, 148.10, 146.81, 139.21, 137.37, 130.72, 121.14, 120.76, 117.62, 105.91, 104.76, 81.82, 79.82, 77.20, 55.80, 54.21, 39.31. 34.34. 33.99, 28.38, 27.81, 26.60. **HRMS (ESI):** m/z $[M+H]^+$ calcd for $C_{29}H_{41}N_4O_5$: 525.3071, found: 525.3078.

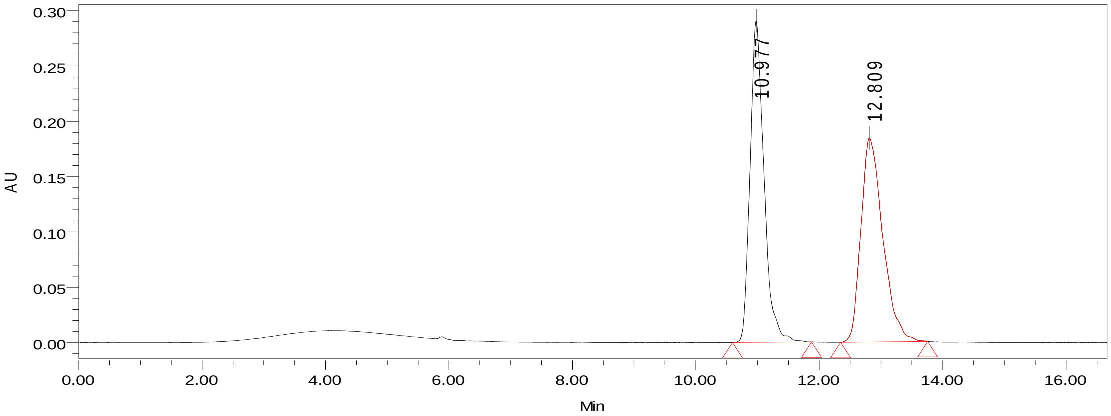

Entry	Retention time	Area	Area (%)	Height	Int type
1	10.977	4581450	50.20	290530	bb
2	12.809	4545752	49.80	184187	bb

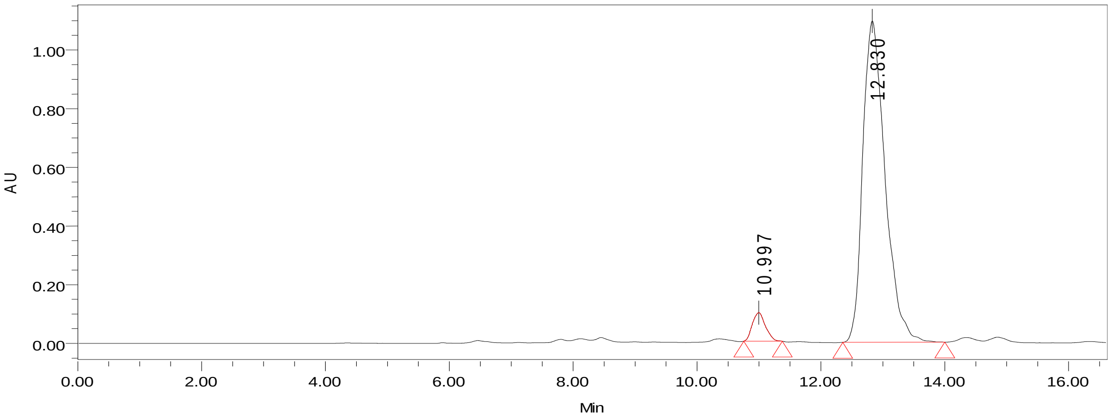

Entry	Retention time	Area	Area (%)	Height	Int type
1	10.997	1425551	4.94	97383	bb
2	12.830	27433679	95.06	1094964	bb

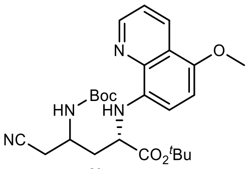

3bg

***tert*-butyl (2*S*)-4-((*tert*-butoxycarbonyl)amino)-5-cyano-2-((5-methoxyquinolin-8-yl)amino)pentanoate (3bg):** yellow oil. 33.4 mg, 71% yield. D.r. = 1:1 determined by ^{1}H NMR analysis, 94%/93% ee determined by HPLC on a Chiralpak IC-H column (hexane/EtOH = 2/1, flow rate = 0.5 mL/min, detection at 261.8 nm, First diastereoisomer: t_{minor} = 14.1 min, t_{major} = 16.3 min. Second diastereoisomer: t_{major} = 14.7 min, t_{minor} = 30.2 min), **^{1}H NMR** (300 MHz, $CDCl_3$) δ 8.20 (s, 1H), 8.50 (d, *J* = 8.4 Hz, 1H), 7.41 (q, *J* = 3.9 Hz, 1H), 6.71 (d, *J* = 8.1 Hz, 1H), 6.64 (d, *J* = 8.1 Hz, 1H), 6.18 (br, 1H), 5.31 (d, *J* = 7.2 Hz, 0.5H), 5.20 (d, *J* = 6.9 Hz, 0.5H), 4.19 (br, 2H), 3.92 (s, 3H), 2.87 - 2.63 (m, 2H), 2.32 - 2.20 (m, 1H), 2.09 - 2.02 (m, 1H), 1.43 (d, *J* = 6.3 Hz, 9H), 1.38 (d, *J* = 5.7 Hz, 9H). **^{13}C NMR** (75 MHz, $CDCl_3$) δ 172.26, 172.08, 154.90, 148.02, 147.95, 146.91, 146.77, 139.20, 137.41, 137.14, 130.76, 121.09, 120.66, 117.32, 106.49, 106.28, 104.86, 104.76, 82.13, 80.03, 55.74, 55.34, 45.41, 36.26, 35.79, 28.20, 27.85, 23.76. **HRMS (ESI):** m/z $[M+H]^+$ calcd for $C_{25}H_{35}N_4O_5$: 471.2602, found: 471.2599.

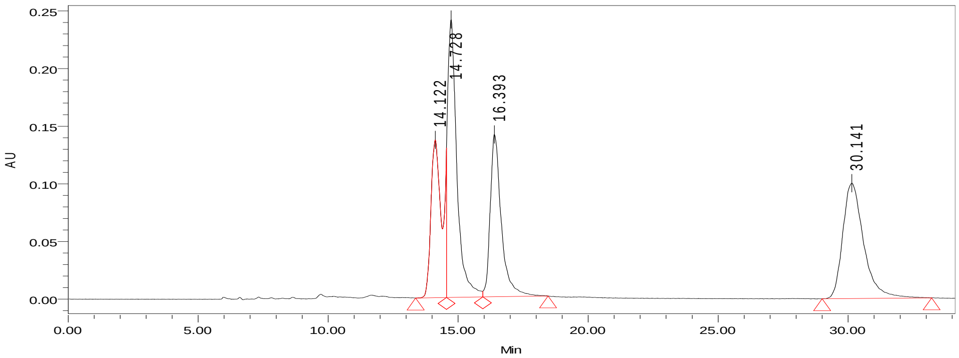

Entry	Retention time	Area	Area (%)	Height	Int type
1	14.122	3953118	20.04	136904	bv
2	14.728	5977393	30.30	241071	vv
3	16.393	4195165	21.27	140908	vb
4	30.141	5599024	28.39	100153	bb

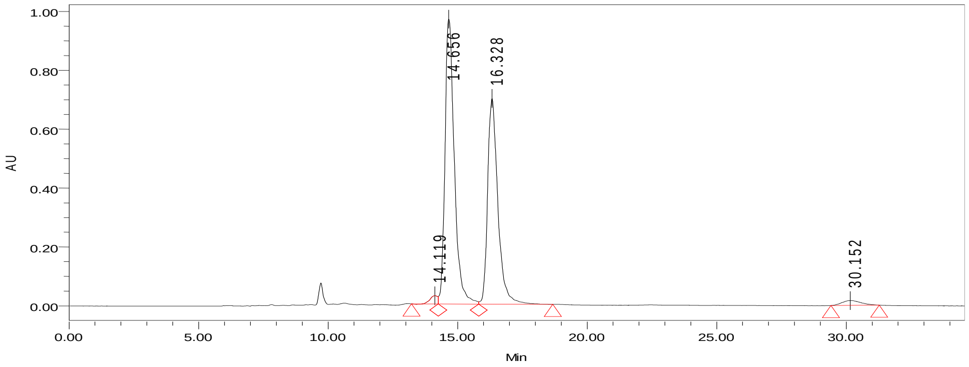

Entry	Retention time	Area	Area (%)	Height	Int type
1	14.119	595495	1.32	28337	bv
2	14.656	24363540	54.10	967366	vv
3	16.328	19246970	42.74	698720	vb
4	30.152	825333	1.83	16284	bb

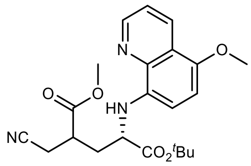

3bh

1-(*tert*-butyl) 5-methyl (2*S*)-4-(cyanomethyl)-2-((5-methoxyquinolin-8-yl)amino)pentanedioate (3bh): yellow oil. 30.6 mg, 74% yield. D.r. = 1:1 determined by ^{1}H NMR analysis, 91%/91% ee determined by HPLC on a Chiralpak IC-H column (hexane/EtOH = 2/1, flow rate = 0.5 mL/min, detection at 260.6 nm, First diastereoisomer: t_{major} = 26.6 min, t_{minor} = 46.2 min. Second diastereoisomer: t_{major} =

27.4 min, t_{minor} = 35.0 min), **^{1}H NMR** (300 MHz, $CDCl_3$) δ 8.79 (d, *J* = 1.2 Hz, 1H), 8.49 (d, *J* = 7.5 Hz, 1H), 7.39 (q, *J* = 4.2 Hz, 1H), 6.73 (d, *J* = 8.1 Hz, 1H), 6.61 (t, *J* = 7.8 Hz, 1H), 6.19 (s, 1H), 4.23 - 4.20 (m, 1H), 3.92 (s, 3H), 3.74 (s, 1.5H), 3.72 (s, 1.5H), 3.13 - 3.08 (m, 1H), 2.77 - 2.71 (m, 2H), 2.56 - 2.40 (m, 1H), 2.31 - 2.20 (m, 1H), 1.38 (s, 9H). **^{13}C NMR** (75 MHz, $CDCl_3$) δ 172.62, 172.43, 172.02, 147.99, 147.91, 146.73, 146.65, 139.08, 137.41, 137.22, 130.76, 121.12, 120.72, 117.51, 117.35, 105.76, 105.63, 104.89, 82.10, 55.77, 55.47, 55.34, 52.58, 38.79, 38.36, 33.77, 33.48, 27.88, 19.65, 19.49. **HRMS (ESI):** m/z $[M+H]^+$ calcd for $C_{22}H_{28}N_3O_5$: 414.2023, found: 414.2025.

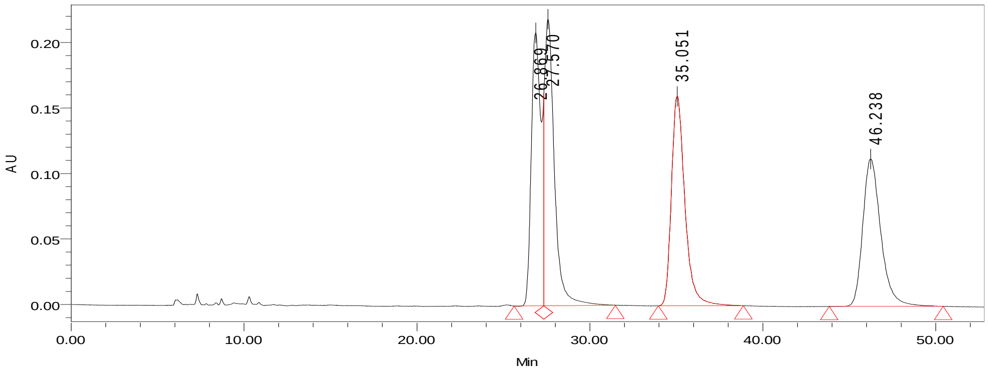

Entry	Retention time	Area	Area (%)	Height	Int type
1	26.869	8366728	24.79	208374	bv
2	27.570	8560803	25.36	218630	vb
3	35.051	8682775	25.72	159833	bb
4	46.238	8144544	24.13	112542	bb

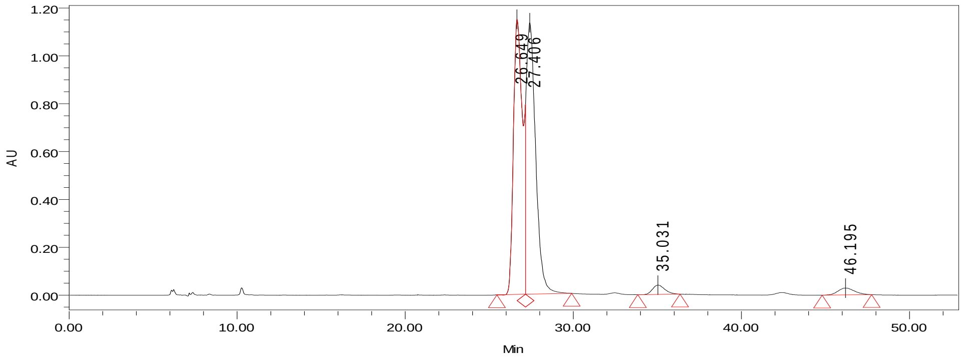

Entry	Retention time	Area	Area (%)	Height	Int type
1	26.649	43584716	47.81	1149688	bv
2	27.406	43622234	47.85	1134313	vb
3	35.031	2009329	2.20	39135	bb
4	46.195	1951757	2.14	28598	bb

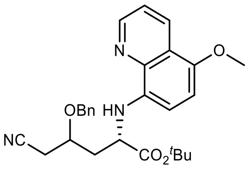

3bi

***tert*-butyl (2*S*)-4-(benzyloxy)-5-cyano-2-((5-methoxyquinolin-8-yl)amino)pentanoate (3bi):** yellow oil. 32.3 mg, 70% yield. D.r. = 1:1 determined by ^{1}H NMR analysis, first diastereoisomer (**3bi-1**): 83% ee determined by HPLC on a Chiralpak IA-H column (hexane/EtOH = 3/1, flow rate = 1.0 mL/min, detection at 261.8 nm, t_{major} = 8.9 min, t_{minor} = 12.3 min), **^{1}H NMR** (300 MHz, $CDCl_3$) δ 8.79 (d, *J* =3.9 Hz, 1H), 8.49 (d, *J* = 8.4 Hz, 1H), 7.44 - 7.29 (m, 6H), 6.72 (d, *J* = 8.4 Hz,1H), 6.61 (d, *J* = 8.4 Hz,1H), 6.40 (d, *J* = 5.7 Hz, 1H), 4.68 (dd, *J* = 11.7 Hz, 15.3 Hz, 2H), 4.27 (d, *J* = 4.8Hz ,1H), 4.04 - 3.98 (m, 1H), 3.92 (s, 3H), 2.67 - 2.52 (m, 2H), 2.41 - 2.23 (m, 2H), 1.39 (s, 9H). **^{13}C NMR** (75 MHz, $CDCl_3$) δ 172.24, 147.93, 146.38, 145.29, 139.01, 137.34, 137.29, 130.70, 128.49, 128.10, 127.99, 121.16, 120.70, 117.23, 105.25, 105.07, 81.88, 72.37, 72.13, 55.82, 54.24, 36.96, 27.93, 23.15. **HRMS (ESI):** m/z $[M+H]^+$ calcd for $C_{27}H_{32}N_3O_4$: 462.2387, found: 462.2386.

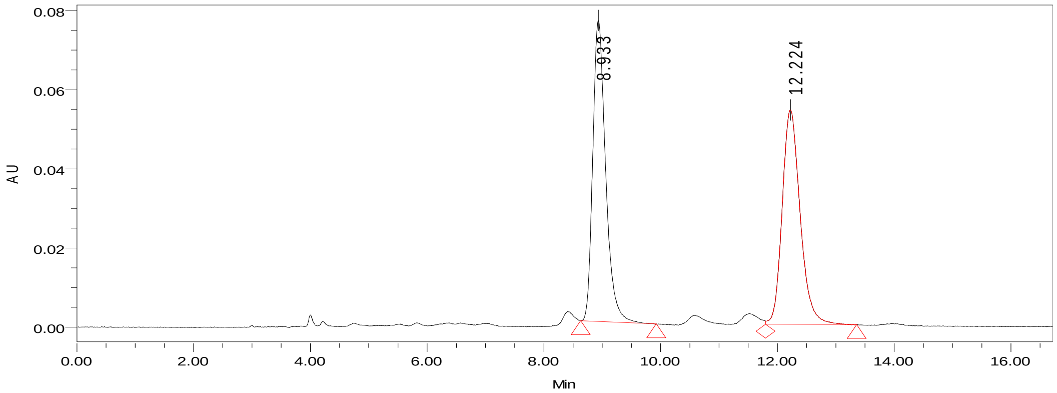

Entry	Retention time	Area	Area (%)	Height	Int type
1	8.933	1166249	50.19	76083	bb
2	12.224	1157201	49.81	54197	vb

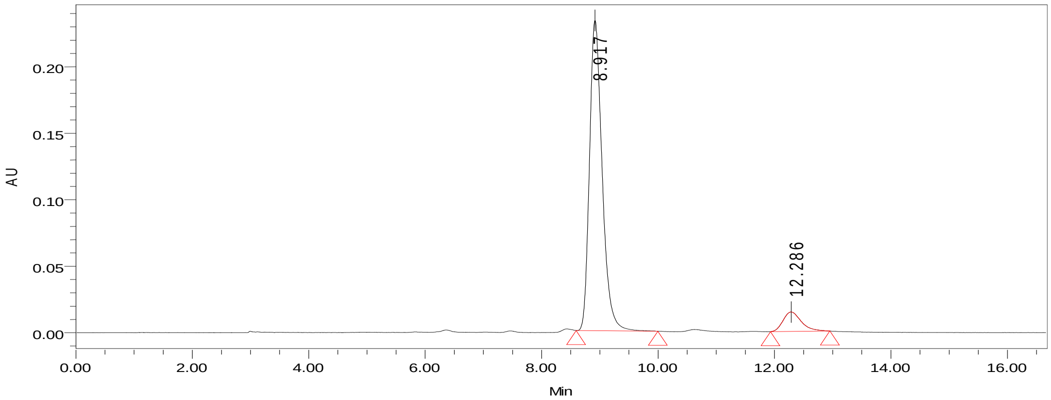

Entry	Retention time	Area	Area (%)	Height	Int type
1	8.917	3523742	91.74	233317	bb
2	12.286	317463	8.26	14631	bb

Second diastereoisomer(**3bi-2**): 94% ee determined by HPLC on a Chiralpak IA-H column (hexane/EtOH = 3/1, flow rate = 1.0 mL/min, detection at 261.8 nm, t_{minor} = 9.2 min, t_{major} = 12.0 min), **^{1}H NMR** (300 MHz, $CDCl_3$) δ 8.76 (d, *J* = 3.9 Hz, 1H), 8.51 (d, *J* = 8.4 Hz, 1H), 7.42 - 7.30 (m, 6H), 6.71 (d, *J* = 8.4 Hz, 1H), 6.59 (d, *J* = 8.1 Hz, 1H), 6.20 (s, 0.8H), 4.64 (d, *J* = 11.1 Hz, 1H), 4.54 - 4.48 (m, 1H), 4.36 (d, *J* = 8.1 Hz, 1H), 4.14 - 4.12 (m, 1H), 3.93 (s, 3H), 2.73 - 2.55 (m, 2H), 2.40 - 2.33 (m, 1H), 2.10 - 2.03 (m, 1H), 1.35 (s, 9H). **^{13}C NMR** (75 MHz, $CDCl_3$) δ 172.87, 147.86, 146.50, 137.83, 137.26, 130.83, 128.49, 128.42, 128.04, 127.92, 127.88, 121.17, 120.66, 117.20, 105.72, 105.06, 81.66, 72.63, 71.61, 55.84, 54.19, 38.06, 27.92, 23.30. **HRMS (ESI):** m/z $[M+H]^+$ calcd for $C_{27}H_{32}N_3O_4^+$: 462.2387, found: 462.2386.

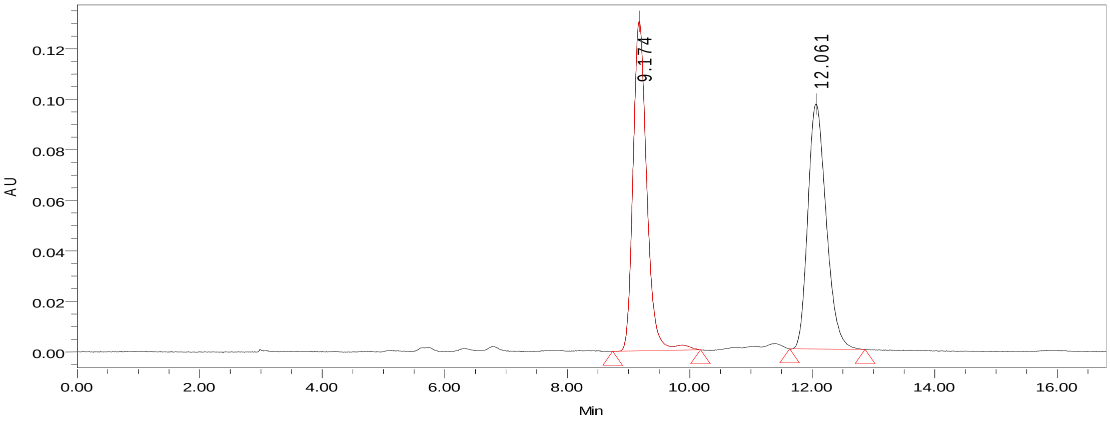

Entry	Retention time	Area	Area (%)	Height	Int type
1	9.174	2032675	50.08	130406	bb
2	12.061	2025867	49.92	97008	bb

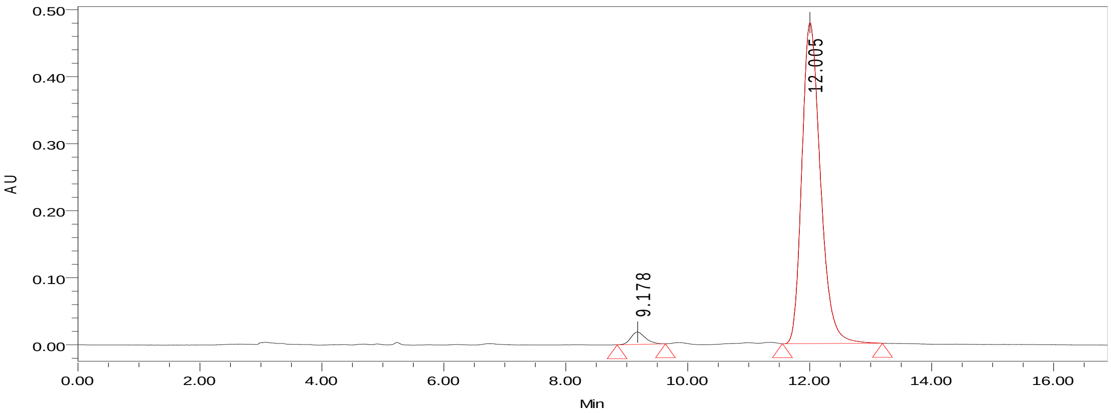

Entry	Retention time	Area	Area (%)	Height	Int type
1	9.178	302895	2.91	18388	bb
2	12.005	10123452	97.09	478806	bb

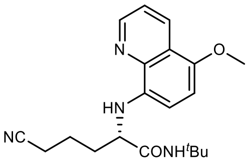

3ca

(*S*)-*N*-(*tert*-butyl)-5-cyano-2-((5-methoxyquinolin-8-yl)amino)pentanamide (3ca): yellow oil. 24.1 mg, 68% yield. 90% ee determined by HPLC on a Chiralpak IA-H column (hexane/EtOH = 2/1, flow rate = 0.5 mL/min, detection at 260.6 nm, t_{minor} = 8.1 min, t_{major} = 9.3 min), $[\alpha]_D^{25.7}$ 5.00 (*c* 10.00 mg/mL, DCM). **^{1}H NMR** (300 MHz, $CDCl_3$) δ 8.81 (d, *J* = 1.5 Hz, 1H), 8.56 (d, *J* = 8.4 Hz, 1H), 7.47 (q, *J* = 4.2 Hz, 1H), 6.87 (s, 1H), 6.74 (d, *J* = 8.1 Hz, 1H), 6.55 (d, *J* = 8.1 Hz, 1H), 6.01 (s, 1H), 3.94 (s, 3H), 3.62 (t, *J* = 6.0 Hz, 1H), 2.45 (t, *J* = 6.9 Hz, 2H), 2.19 - 1.88 (m, 4H), 1.31 (s, 9H). **^{13}C NMR** (75 MHz, $CDCl_3$) δ 172.23, 148.13, 147.24, 138.72, 137.24, 131.22, 120.99, 120.91, 119.26, 106.83, 104.92, 60.63, 55.79, 50.75, 32.65, 28.54, 22.51, 17.03. **HRMS (ESI):** m/z $[M+H]^+$ calcd for $C_{20}H_{27}N_4O_2$: 355.2129, found: 355.2129.

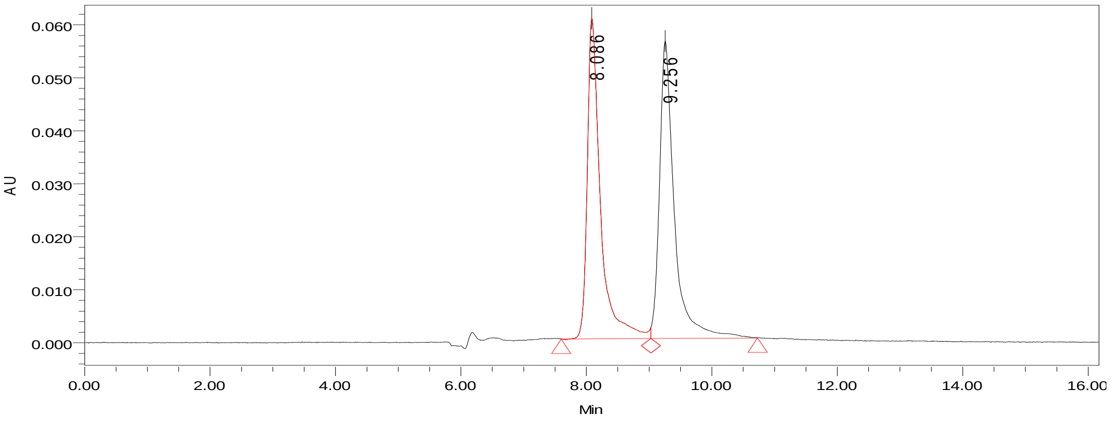

Entry	Retention time	Area	Area (%)	Height	Int type
1	8.086	888809	49.16	60460	bv
2	9.256	919240	50.84	56095	vb

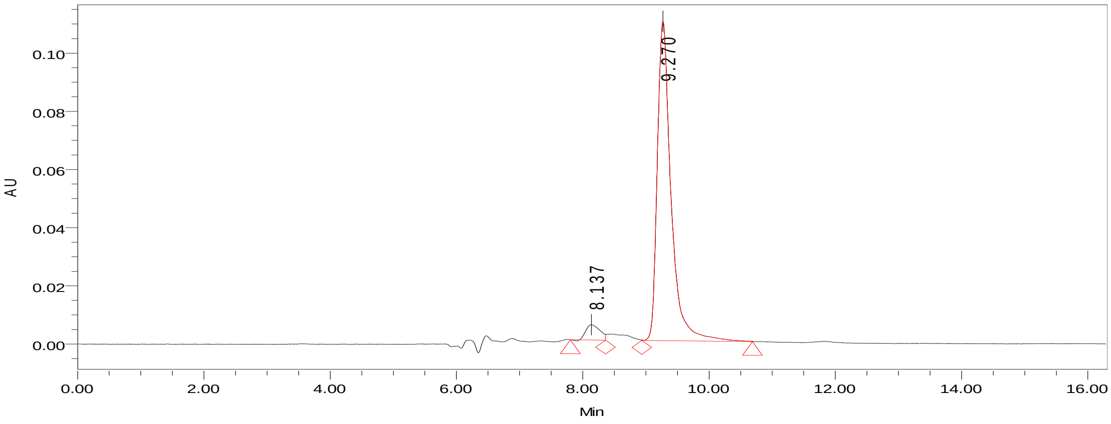

Entry	Retention time	Area	Area (%)	Height	Int type
1	8.137	83244	4.78	5251	bv
2	9.270	1659923	95.22	109727	vb

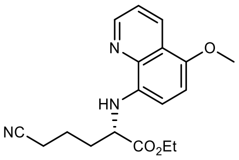

3da

ethyl (*S*)-5-cyano-2-((5-methoxyquinolin-8-yl)amino)pentanoate (3da): yellow solid. M. p. 58 - 60 °C. 25.2 mg, 77% yield. 89% ee determined by HPLC on a Chiralpak IA-H column (hexane/EtOH = 2/1, flow rate = 0.5 mL/min, detection at 261.8 nm, t_{minor} = 18.1 min, t_{major} = 27.2 min), $[\alpha]_D^{25.8}$ 28.00 (*c* 10.00 mg/mL, DCM). **^{1}H NMR** (300 MHz, $CDCl_3$) δ 8.79 (d, *J* = 3.9 Hz, 1H), 8.50 (d, *J* = 8.4 Hz, 1H), 7.42 - 7.38 (m, 1H), 6.72 (d, *J* = 7.8 Hz, 1H), 6.56 (d, *J* = 7.8 Hz, 1H), 6.20 (s, 1H), 4.22 - 4.15 (m, 3H),

3.92 (s, 3H), 2.43 (t, J = 6.6 Hz, 2H), 2.18 - 2.06 (m, 2H), 1.93 - 1.89 (m, 2H), 1.22 (t, J = 7.2 Hz, 3H). **^{13}C NMR** (75 MHz, $CDCl_3$) δ 173.35, 147.94, 146.51, 138.90, 137.30, 130.80, 121.17, 120.75, 119.18, 105.15, 104.95, 61.19, 56.20, 55.78, 31.59, 21.96, 16.97, 14.71. **HRMS (ESI):** m/z $[M+H]^+$ calcd for $C_{18}H_{22}N_3O_3$: 328.1656, found: 328.1656.

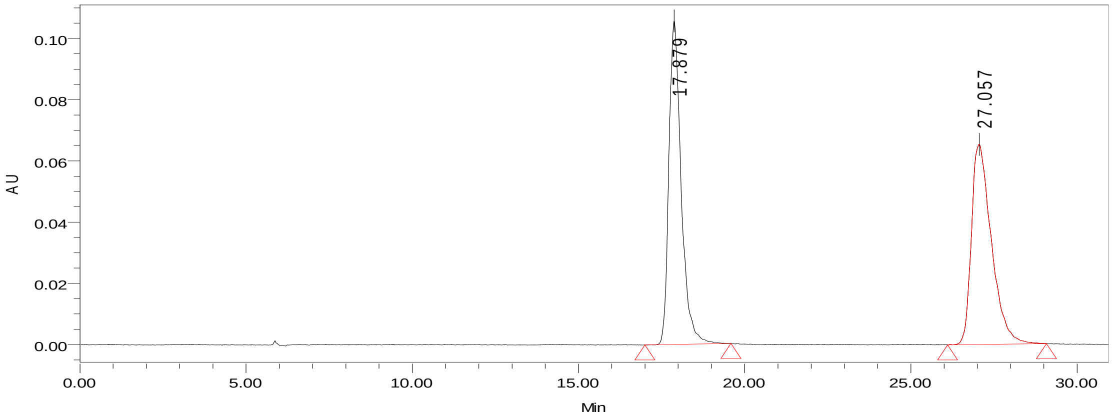

Entry	Retention time	Area	Area (%)	Height	Int type
1	17.879	2715315	49.86	105638	bb
2	27.057	2730442	50.14	65423	bb

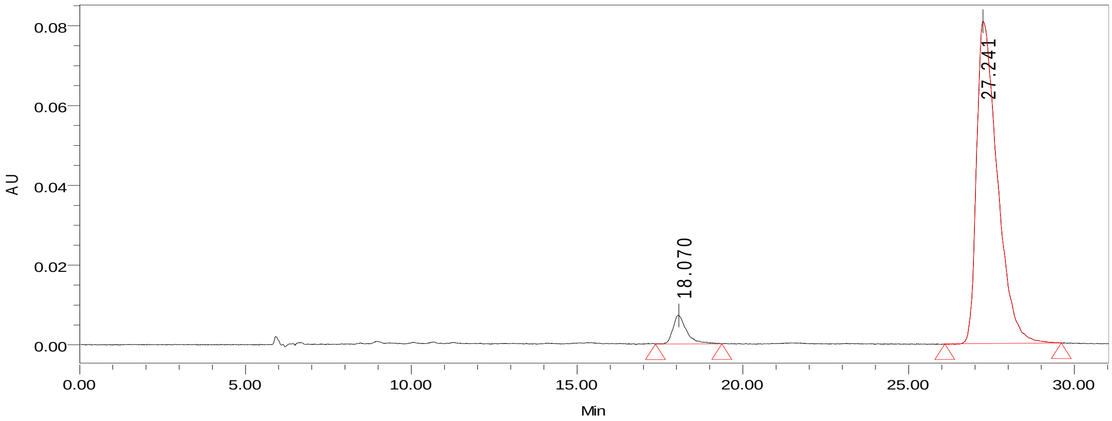

Entry	Retention time	Area	Area (%)	Height	Int type
1	18.070	206383	5.52	7158	bb
2	27.241	3531426	94.48	80802	bb

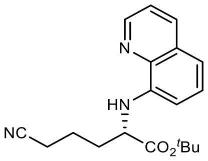

3ea

***tert*-butyl (*S*)-5-cyano-2-(quinolin-8-ylamino)pentanoate (3ea):** white solid. M. p.

95 - 97 °C. 21.2 mg, 65% yield. 89% ee determined by HPLC on a Chiralpak IA-H column (hexane/EtOH = 2/1, flow rate = 0.5 mL/min, detection at 257.1 nm, t_{major} = 10.3 min, t_{minor} = 11.2 min), $[\alpha]_D^{25.9}$ 10.00 (*c* 10.00 mg/mL, DCM). **^{1}H NMR** (300 MHz, $CDCl_3$) δ 8.75 (dd, *J* = 1.5 Hz, 4.2 Hz, 1H), 8.05 (dd, *J* = 1.5 Hz, 8.4 Hz, 1H), 7.39 - 7.32 (m, 2H), 7.10 (d, *J* = 7.5 Hz, 1H), 6.65 (d, *J* = 7.8 Hz, 2H), 4.20 (dd, *J* = 6.3 Hz, 14.4 Hz, 1H), 2.44 (t, *J* = 0.9 Hz, 2H), 2.42 - 2.01 (m, 2H), 1.94 - 1.85 (m, 2H), 1.43 (s, 9H). **^{13}C NMR** (75 MHz, $CDCl_3$) δ 172.04, 147.26, 143.41, 138.37, 135.89, 128.63, 127.34, 121.50, 119.17, 115.21, 105.39, 82.03, 56.15, 31.40, 27.96, 21.80, 17.04. **HRMS (ESI):** m/z $[M+H]^+$ calcd for $C_{19}H_{24}N_3O_2$: 326.1863, found: 326.1862.

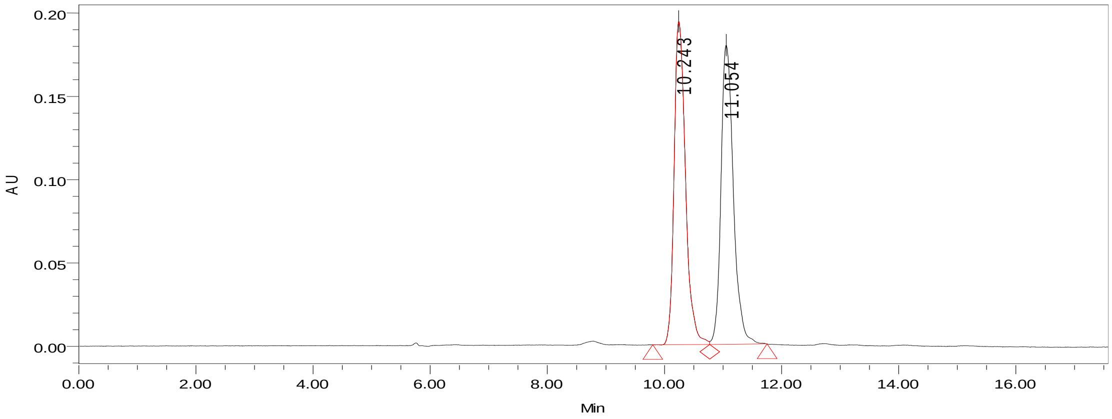

Entry	Retention time	Area	Area (%)	Height	Int type
1	10.243	2620839	49.81	194001	bv
2	11.054	2641148	50.19	179512	vb

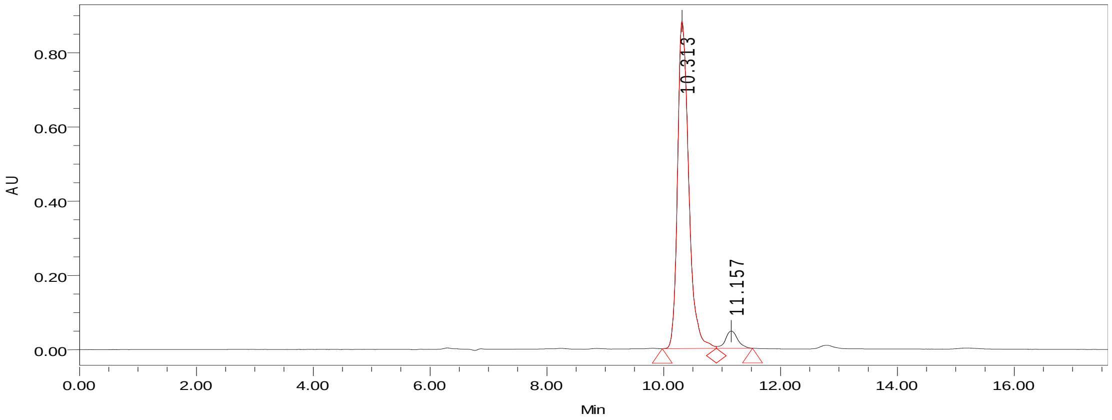

Entry	Retention time	Area	Area (%)	Height	Int type
1	10.313	11751977	94.57	882379	bv
2	11.157	674697	5.43	46282	vb

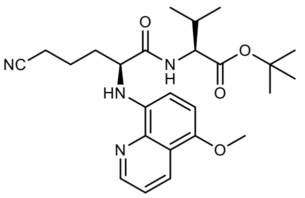

3fa

***tert*-butyl ((*S*)-5-cyano-2-((5-methoxyquinolin-8-yl)amino)pentanoyl)-*L*-valinate (3fa):** Yellow oil, 31.8mg, 70% yield, d.r. > 20:1. $[\alpha]_D^{26.0}$ -1.00 (*c* 10.00 mg/mL, DCM). **^{1}H NMR** (300 MHz, $CDCl_3$) δ 8.81 (d, *J* = 3.9 Hz, 1H), 8.54 (d, *J* = 8.4 Hz, 1H), 7.47 - 7.42 (m, 2H), 6.72 (d, *J* = 8.4 Hz, 1H), 6.58 (d, *J* = 8.4 Hz, 1H), 6.09 (s, 1H), 4.40 (dd, *J* = 5.4 Hz, 9.0 Hz, 1H), 3.92 (s, 3H), 3.79 - 3.76 (m, 1H), 2.44 (t, *J* = 7.2 Hz, 2H), 2.28 - 2.18 (m, 1H), 2.12 - 2.00 (m, 2H), 1.97 - 1.85 (m, 2H), 1.23 (s, 9H), 0.89 (dd, *J* = 6.6 Hz, 15.9 Hz, 6H). **^{13}C NMR** (75 MHz, $CDCl_3$) δ 172.99, 169.95, 148.07, 147.27, 138.69, 137.12, 131.13, 120.97, 120.81, 119.12, 107.15, 104.95, 81.58, 60.13, 57.58, 55.76, 32.66, 31.39, 27.64, 22.54, 18.97, 17.75, 16.95. **HRMS (ESI):** m/z $[M+H]^+$ calcd for $C_{25}H_{35}N_4O_4$: 455.2653, found: 455.2655.

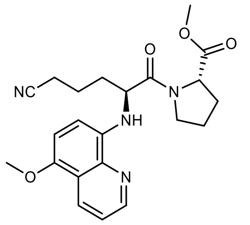

3ga

methyl ((*S*)-5-cyano-2-((5-methoxyquinolin-8-yl)amino)pentanoyl)-*L*-prolinate (3ga): Yellow solid, M. p. 130 - 132 ºC. 31.2 mg, 76% yield, d.r. > 20:1. $[\alpha]_D^{26.1}$ -16.67 (*c* 9.00 mg/mL, DCM). **^{1}H NMR** (300 MHz, $CDCl_3$) δ 8.78 (d, *J* = 3.0 Hz, 1H), 8.48 (d, *J* = 8.4 Hz, 1H), 7.40 - 7.38 (m, 1H), 6.73 (d, *J* = 8.1 Hz, 1H), 6.56 (d, *J* = 8.4 Hz, 1H), 6.45 (s, 0.8H), 4.54 (t, *J* = 6.0 Hz, 1H), 4.43 (t, *J* = 6.0 Hz, 1H), 3.92 (s, 3H), 3.79 - 3.73 (m, 5H), 2.44 - 2.40 (m, 2H), 2.20 - 1.90 (m, 8H). **^{13}C NMR** (75 MHz, $CDCl_3$) δ 172.47, 171.14, 148.00, 146.34, 139.06, 137.45, 130.70, 121.30, 120.78, 119.55, 105.06, 104.75, 59.05, 55.85, 54.68, 52.21, 46.88, 30.41, 28.76, 25.10, 21.26, 17.08. **HRMS (ESI):** m/z $[M+H]^+$ calcd for $C_{22}H_{27}N_4O_4$: 411.2027, found: 411.2026.

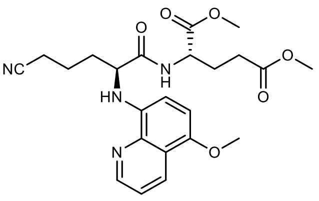

3ha

dimethyl ((*S*)-5-cyano-2-((5-methoxyquinolin-8-yl)amino)pentanoyl)-*L*-glutamate (3ha): Yellow solid, M. p. 101 - 103 ºC. 31.0 mg, 68% yield, d.r. > 20:1. $[\alpha]_D^{26.2}$ -1.00 (*c* 10.00 mg/mL, DCM). **^{1}H NMR** (300 MHz, $CDCl_3$) δ 8.81 (d, *J* = 3.9 Hz, 1H), 8.54 (d, *J* = 8.4 Hz, 1H), 7.57 (d, *J* = 8.1 Hz, 1H), 7.44 (q, *J* = 4.2 Hz, 1H), 6.75 (d, *J* = 8.1 Hz ,1H), 6.61 (d, *J* = 8.4 Hz, 1H), 6.09 (s, 1H), 4.60 (dd, *J* = 8.1 Hz, 13.2 Hz, 1H), 3.93 (s, 3H) ,3.83 - 3.77 (m, 1H), 3.64 (s, 3H), 3.61 (s, 3H), 2.45 - 2.33 (m, 4H), 2.27 - 2.18 (m, 2H), 2.04 - 1.88 (m, 4H). **^{13}C NMR** (75 MHz, $CDCl_3$) δ 173.49, 172.75, 171.50, 148.09, 147.26, 138.70, 137.01, 131.04, 120.87, 120.80, 119.08, 107.13, 104.74, 59.85, 55.68, 52.25, 51.70, 51.35, 32.43, 29.96, 26.74, 22.44, 16.87. **HRMS (ESI):** m/z $[M+H]^+$ calcd for $C_{23}H_{29}N_4O_6$: 457.2082, found: 457.2081.

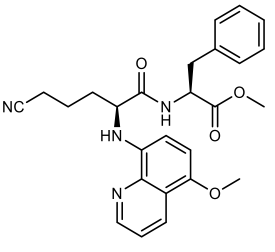

3ia

methyl ((*S*)-5-cyano-2-((5-methoxyquinolin-8-yl)amino)pentanoyl)-*L*-phenylalaninate (3ia): Yellow solid, M. p. 130 - 132 ºC. 32.2 mg, 70% yield, d.r. > 20:1. $[\alpha]_D^{26.2}$ -11.00 (*c* 10.50 mg/mL, DCM). **^{1}H NMR** (300 MHz, $CDCl_3$) δ 8.80 (d, *J* = 4.4 Hz, 1H), 8.54 (d, *J* = 8.4 Hz, 1H), 7.46 (q, *J* = 4.2 Hz, 1H), 7.35 (d, *J* = 8.4 Hz, 1H), 7.23 - 7.19 (m, 3H), 7.10 (d, *J* = 6.9 Hz, 2H), 6.71 (d, *J* = 8.4 Hz, 1H), 6.55 (d, *J* = 8.1 Hz, 1H), 5.96 (s, 1H), 4.93 (q, *J* = 8.4 Hz, 1H), 3.93 (s, 3H), 3.75 -3.68 (m, 1H), 3.63 (s, 3H), 3.23 (dd, *J* = 5.1 Hz, 13.8 Hz, 1H), 2.92 (dd, *J* = 8.4 Hz, 13.8 Hz, 1H), 2.33 - 2.28 (m, 2H), 2.00 - 1.88 (m, 2H), 1.71 - 1.64 (m, 2H). **^{13}C NMR** (75 MHz, $CDCl_3$) δ 172.91, 171.51, 148.07, 147.31, 138.70, 136.94, 135.94, 131.15, 129.05, 128.51, 127.00, 120.93, 120.82, 119.09, 107.32, 104.82, 59.79, 55.75, 52.64, 55.22,

37.79, 32.27, 22.00, 16.93. **HRMS (ESI):** m/z $[M+H]^+$ calcd for $C_{26}H_{29}N_4O_4$: 461.2183, found: 461.2185.

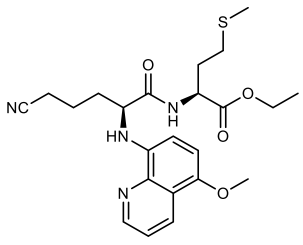

3ja

ethyl ((*S*)-5-cyano-2-((5-methoxyquinolin-8-yl)amino)pentanoyl)-*L*-methioninate (3ja): Yellow solid, M. p. 105 - 107 ºC. 32.1 mg, 70% yield, d.r. > 20:1. $[\alpha]_D^{26.4}$ -3.33 (*c* 10.00 mg/mL, DCM). **^{1}H NMR** (300 MHz, $CDCl_3$) δ 8.81 (s, 1H), 8.55 (d, *J* = 8.4 Hz, 1H), 7.57 (d, *J* = 7.8 Hz, 1H), 7.47 - 7.44 (m, 1H), 6.75 (d, *J* = 7.8 Hz, 1H), 6.62 (d, *J* = 8.4 Hz, 1H), 6.09 (s, 1H), 4.68 (dd, *J* = 6.3 Hz, 13.5 Hz, 1H), 4.09 (dd, *J* = 6.6 Hz, 13.5 Hz, 2H), 3.93 (s, 3H), 3.80 (s, 1H), 2.46 (dd, *J* = 8.1 Hz, 26.1 Hz, 4H), 2.24 - 1.92 (m, 9H), 1.14 (t, *J* = 7.2Hz, 3H). **^{13}C NMR** (75 MHz, $CDCl_3$) δ 173.33, 171.20, 148.17, 147.40, 138.76, 137.07, 131.22, 121.01, 120.91, 119.13, 107.24, 104.86, 61.43, 59.99, 55.79, 51.43, 32.54, 31.38, 30.08, 22.58, 17.01, 15.45, 13.99. **HRMS (ESI):** m/z $[M+H]^+$ calcd for $C_{23}H_{31}N_4O_4$: 459.2061, found: 459.2063.

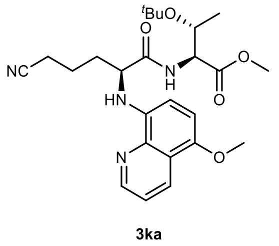

3ka

methyl *O*-(*tert*-butyl)-*N*-((*S*)-5-cyano-2-((5-methoxyquinolin-8-yl)amino)pentanoyl)-*L*-threoninate (3ka): Yellow oil, 29.7 mg, 63% yield, d.r. > 20:1. $[\alpha]_D^{26.5}$ 7.00 (*c* 10.00 mg/mL, DCM). **^{1}H NMR** (300 MHz, $CDCl_3$) δ 8.80 (d, *J* = 1.8 Hz, 1H), 8.54 (d, *J* = 8.4 Hz, 1H), 7.59 (d, *J* = 8.7 Hz, 1H), 7.43 (dd, *J* = 3.9 Hz, 8.1 Hz, 1H), 6.75 (dd, *J* = 8.1 Hz, 19.5 Hz, 2H), 6.12 (s, 1H), 4.46 (d, *J* = 9.0 Hz, 1H), 4.18 - 4.11 (m, 1H), 3.94 (s, 3H), 3.86 (s, 1H), 3.62 (s, 3H), 2.44 (t, *J* = 6.9 Hz, 2H), 2.27 -

2.22 (m, 1H), 2.11 - 1.95 (m, 3H), 1.14 (d, J = 5.7 Hz, 3H), 0.92 (s, 9H). **^{13}C NMR** (75 MHz, $CDCl_3$) δ 173.81, 170.89, 148.01, 147.11, 138.83, 137.30, 131.02, 120.92, 120.72, 119.19, 107.40, 105.04, 73.89, 67.08, 59.95, 57.81, 55.81, 52.00, 32.58, 28.09, 22.58, 21.12, 17.01. **HRMS (ESI):** m/z $[M+H]^+$ calcd for $C_{25}H_{35}N_4O_5$: 471.2602, found: 471.2605.

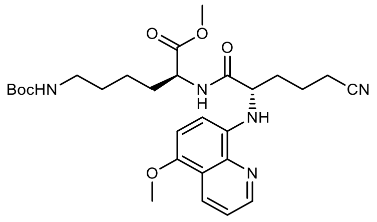

3la

methyl N^6-(*tert*-butoxycarbonyl)-N^2-((*S*)-5-cyano-2-((5-methoxyquinolin-8-yl)amino)pentanoyl)-*L*-lysinate (3la): Yellow solid, M. p. 79 - 81 °C. 39.5 mg, 73% yield, d.r. > 20:1. $[\alpha]_D^{26.5}$ -1.00 (*c* 10.00 mg/mL, DCM). **^{1}H NMR** (300 MHz, $CDCl_3$) δ 8.81 (s, 1H), 8.55 (d, J = 8.1 Hz, 1H), 7.48 - 7.41 (m, 2H), 6.76 (d, J = 8.1 Hz, 1H), 6.63 (d, J = 8.4 Hz, 1H), 6.07 (s, 1H), 4.61 - 4.53 (m, 2H), 3.94 (s, 3H), 3.79 (s, 1H), 3.63 (s, 3H), 3.11 - 3.03 (m, 2H), 2.44 (t, J = 6.9 Hz, 2H), 2.23 - 2.21 (m, 1H), 2.08 - 1.59 (m, 7H), 1.43 (s, 9H), 1.33 - 1.30 (m, 2H). **^{13}C NMR** (75 MHz, $CDCl_3$) δ 173.36, 172.17, 155.93, 148.20, 147.44, 138.83, 137.13, 131.20, 120.98, 120.90, 119.17, 107.45, 104.83, 79.09, 60.12, 55.78, 52.21, 51.84, 40.18, 32.59, 31.71, 29.51, 28.38, 22.73, 22.56, 16.99. **HRMS (ESI):** m/z $[M+H]^+$ calcd for $C_{28}H_{40}N_5O_6$: 542.2973, found: 542.2974.

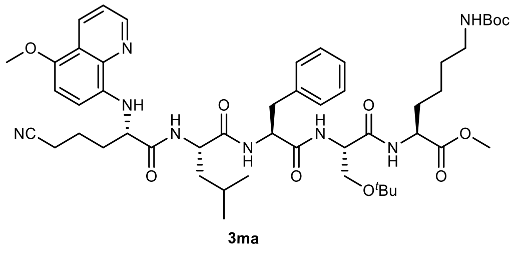

3ma

methyl N^6-(*tert*-butoxycarbonyl)-N^2-(*O*-(*tert*-butyl)-*N*-((*S*)-5-cyano-2-((5-methoxyquinolin-8-yl)amino)pentanoyl)-*L*-leucyl-*L*-phenylalanyl-*L*-seryl)-*L*-

lysinate (3ma): Yellow solid, M. p. 173 - 175 °C. 59.5 mg, 63% yield, d.r. > 20:1. $[\alpha]_D^{26.6}$ 1.00 (*c* 10.00 mg/mL, DCM). **^{1}H NMR** (300 MHz, $CDCl_3$) δ 8.82 (s, 1H), 8.56 (d, *J* = 7.8 Hz, 1H), 7.49 - 7.45 (m, 1H), 7.21 - 7.15 (m, 5H), 7.03 - 6.94 (m, 2H), 6.79 - 6.71 (m, 2H), 6.63 - 6.60 (m, 1H), 6.44 (d, *J* = 8.1 Hz, 1H), 6.07 (s, 0.8H), 4.66 - 4.62 (m, 1H), 4.57 - 4.51 (m, 2H), 4.45 - 4.37 (m, 2H), 3.91 (s, 3H), 3.79 - 3.72 (m, 5H), 3.40 - 3.35 (m, 1H), 3.13 - 3.08 (m, 3H), 2.77 - 2.70 (m, 1H), 2.44 (t, *J* = 6.3 Hz, 2H), 2.24 - 2.19 (m, 2H), 2.02 - 1.90 (m, 6H), 1.68 - 1.64 (m, 2H), 1.48 - 1.43 (m, 12H), 1.19 (s, 9H), 0.86 - 0.81 (m, 6H). **^{13}C NMR** (75 MHz, $CDCl_3$) δ 173.64, 172.27, 171.92, 170.36, 169.61, 155.91, 148.25, 147.48, 138.70, 136.87, 136.42, 131.30, 128.95, 128.71, 128.48, 126.85, 121.19, 121.08, 119.02, 106.34, 105.05, 78.97, 74.04, 61.06, 59.52, 55.77, 54.79, 53.19, 52.22, 51.99, 51.53, 40.20, 39.73, 32.23, 31.85, 28.36, 27.32, 24.64, 22.86, 22.61, 22.47, 21.44, 16.91, 14.05. **HRMS (ESI):** m/z $[M+H]^+$ calcd for $C_{50}H_{73}N_8O_{10}$: 945.5444, found: 945.5448.

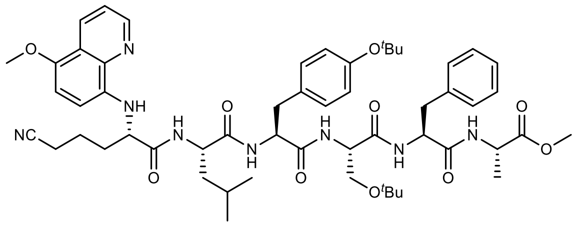

3na

methyl *N*-((*S*)-3-(4-(*tert*-butoxy)phenyl)-2-((*S*)-2-((*S*)-5-cyano-2-((5-methoxyquinolin-8-yl)amino)pentanamido)-4-methylpentanamido)propanoyl)-*O*-(*tert*-butyl)-*L*-seryl-*L*-phenylalanyl-*L*-alaninate (3na): Yellow solid, M. p. 259 - 261 °C. 60.4 mg, 60% yield, d.r. > 20:1. $[\alpha]_D^{26.6}$ -10.00 (*c* 10.00 mg/mL, DCM). **^{1}H NMR** (300 MHz, $CDCl_3$) δ 8.81 (s, 1H), 8.54 (d, *J* = 8.4 Hz, 1H), 7.46 (dd, *J* = 3.0 Hz, 7.5 Hz, 1H), 7.24 - 7.18 (m, 7H), 7.07 - 6.91 (m, 2H), 6.82 - 6.70 (m, 6H), 6.45 (d, *J* = 8.1 Hz, 1H), 6.07 (s, 1H), 4.79 (drs, 1H), 4.52 - 4.34 (m, 4H), 3.88 (s, 3H), 3.81 - 3.75 (m, 1H), 3.70 - 3.66 (m, 4H), 3.36 (t, *J* = 8.1 Hz, 1H), 3.14 - 3.00 (m, 3H), 2.69 - 2.62 (m, 1H), 2.44 (t, *J* = 6.0 Hz, 2H), 2.19 - 2.16 (m, 1H), 2.05 - 1.88 (m, 4H), 1.52 - 1.50 (m, 2H), 1.36 (d, *J* = 6.9 Hz, 3H), 1.27 (s, 9H), 1.08 (s, 9H), 0.86 - 0.81 (m, 6H). **^{13}C NMR** (75 MHz, $CDCl_3$) δ 173.80, 172.84, 172.27, 171.05, 170.39, 169.67, 154.25, 148.18,

147.38, 138.72, 136.94, 136.80, 131.33, 131.01, 129.49, 129.27, 128.35, 126.62, 124.12, 121.20, 121.03, 119.13, 106.47, 105.06, 78.27, 74.12, 59.35, 55.69, 54.86, 53.88, 53.81, 52.29, 51.51, 48.07, 32.15, 31.87, 29.64, 28.76, 27.23, 24.69, 22.90, 22.63, 22.52, 21.49, 18.02, 16.89, 14.07. **HRMS (ESI):** m/z $[M+H]^+$ calcd for $C_{55}H_{75}N_8O_{10}$: 1007.5601, found: 1007.5580.

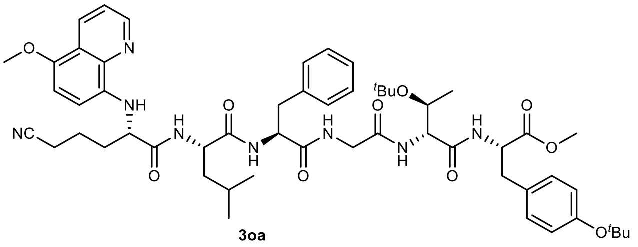

3oa

methyl (2*S*,5*R*,11*S*,14*S*,17*S*)-11-benzyl-2-(4-(*tert*-butoxy)benzyl)-5-((*S*)-1-(*tert*-butoxy)ethyl)-20-cyano-14-isobutyl-17-((5-methoxyquinolin-8-yl)amino)-4,7,10,13,16-pentaoxo-3,6,9,12,15-pentaazaicosanoate (3oa): Yellow solid, M. p. 133 - 135 ºC. 70.5 mg, 70% yield, d.r. > 20:1. $[\alpha]_D^{26.6}$ -24.00 (*c* 10.00 mg/mL, DCM). **^{1}H NMR** (300 MHz, $CDCl_3$) δ 8.83 - 8.81 (m, 1H), 8.56 (d, *J* = 8.4 Hz, 1H), 7.60 (d, *J* = 7.5 Hz, 1H), 7.47 (q, *J* = 4.2 Hz, 1H), 7.23 (d, *J* = 7.8 Hz, 1H), 7.10 - 7.05 (m, 6H), 6.98 - 6.95 (m, 1H), 6.89 - 6.78 (m, 5H), 6.68 (d, *J* = 7.5 Hz, 1H), 6.58 (d, *J* = 8.1 Hz, 1H), 6.07 (d, *J* = 3.3 Hz, 1H), 4.84 (q, *J* = 6.6 Hz, 1H), 4.61 (q, *J* = 6.9 Hz, 1H), 4.45 - 4.38 (m, 2H), 4.11 - 4.08(m, 2H), 3.90 (s, 3H), 3.86-3.84 (m, 2H), 3,65 (s, 3H), 3.10 (dd, *J* = 5.4 Hz, 13.8 Hz, 2H), 2.98 (dd, *J* = 7.8 Hz, 14.1 Hz, 1H), 2.63 (dd, *J* = 8.7 Hz, 13.5 Hz, 1H), 2.43 (t, *J* = 6.9 Hz, 2H), 2.20 - 1.88 (m, 4H), 1.46 - 1.43 (m, 3H), 1.30 (s, 9H), 1.23 (s, 9H), 0.80 - 0.77 (m, 9H). **^{13}C NMR** (75 MHz, $CDCl_3$) δ 173.68, 171.71, 171.66, 170.88, 168.81, 168.01, 154.28, 148.23, 147.40, 138.68, 136.88, 136.57, 131.26, 130.67, 129.61, 128.86, 128.34, 126.67, 124.19, 121.16, 121.05, 119.08, 106.40, 105.11, 78.31, 75.46, 65.94, 59.29, 57.30, 55.72, 54.20, 53.74, 52.14, 51.73, 42.79, 40.29, 37.54, 32.19, 28.70, 28.02, 24.60, 22.82, 22.49, 21.49, 16.88, 16.78. **HRMS (ESI):** m/z $[M+H]^+$ calcd for $C_{55}H_{75}N_8O_{10}$: 1007.5601, found: 1007.5597.

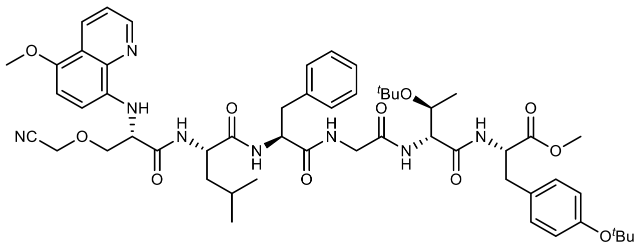

3ob

methyl (4*S*,7*S*,10*S*,16*R*,19*S*)-10-benzyl-19-(4-(*tert*-butoxy)benzyl)-16-((*S*)-1-(*tert*-butoxy)ethyl)-1-cyano-7-isobutyl-4-((5-methoxyquinolin-8-yl)amino)-5,8,11,14,17-pentaoxo-2-oxa-6,9,12,15,18-pentaazaicosan-20-oate (3ob): Yellow solid, M. p. 129 - 131 ºC. 65.6 mg, 65% yield, d.r. > 20:1. $[\alpha]_D^{26.7}$ -13.00 (*c* 10.00 mg/mL, DCM). **^{1}H NMR** (300 MHz, $CDCl_3$) δ 8.86 - 8.85 (m, 1H), 8.57 (d, *J* = 8.4 Hz, 1H), 7.57 (d, *J* = 7.2 Hz, 1H), 7.48 (dd, *J* = 3.6 Hz, 8.1 Hz, 1H), 7.18 (d, *J* = 7.2 Hz, 1H), 7.10 - 7.05 (m, 5H), 6.96 (d, *J* = 5.4 Hz, 1H), 6.89 - 6.81 (m, 6H), 6.63 (d, *J* = 8.4 Hz, 1H), 6.50 (d, *J* = 7.8 Hz, 1H), 6.43 (s, 1H), 4.81 (q, *J* = 6.3 Hz, 1H), 4.58 (q, *J* = 7.2 Hz, 1H), 4.36 - 4.33 (m, 4H), 4.11 - 4.01 (m, 5H), 3.93 (s, 3H), 3.89 - 3.82 (m, 1H), 3.66 (s, 3H), 3.16 - 3.08 (m, 2H), 3,01 - 2.94 (m, 1H), 2.64 (dd, *J* = 9.6 Hz, 13.8 Hz, 1H), 2.02 (s, 1H), 1.46 - 1.43 (m, 2H), 1.30 (s, 9H), 1.24 (s, 9H), 0.82 - 0.78 (m, 9H). **^{13}C NMR** (75 MHz, $CDCl_3$) δ 171.69, 171.67, 171.50, 170.91, 168.84, 168.07, 154.31, 148.37, 147.58, 138.89, 136.77, 136.60, 131.23, 130.69, 129.64, 128.91, 128.38, 126.71, 124.22, 121.23, 121.09, 115.30, 106.61, 105.07, 78.35, 75.52, 71.51, 65.91, 59.57, 57.33, 56.56, 55.77, 54.23, 53.76, 52.17, 52.10, 42.80, 40.11, 37.57, 28.73, 28.04, 24.51, 22.86, 21,37, 16.76. **HRMS (ESI):** m/z $[M+H]^+$ calcd for $C_{54}H_{73}N_8O_{11}$: 1009.5393, found: 1009.5386.

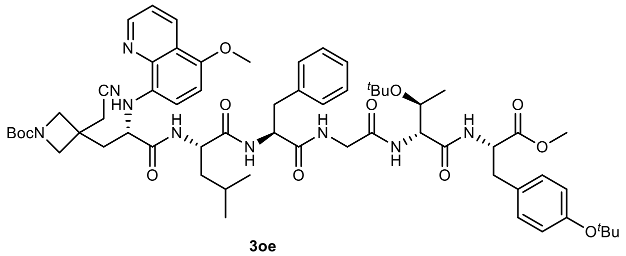

3oe

***tert*-butyl 3-((4*S*,7*R*,13*S*,16*S*,19*S*)-13-benzyl-4-(4-(*tert*-butoxy)benzyl)-7-((*S*)-1-(*tert*-butoxy)ethyl)-16-isobutyl-19-((5-methoxyquinolin-8-yl)amino)-**

3,6,9,12,15,18-hexaoxo-2-oxa-5,8,11,14,17-pentaazaicosan-20-yl)-3-(cyanomethyl)azetidine-1-carboxylate (3oe): Yellow solid, M. p. 122 - 124 ºC. 78.1 mg, 68% yield, d.r. > 20:1. $[\alpha]_D^{26.7}$ -7.00 (*c* 10.00 mg/mL, DCM). **^{1}H NMR** (300 MHz, $CDCl_3$) δ 8.82 (s, 1H), 8.57 (d, *J* = 8.1 Hz, 1H), 7.58 (d, *J* = 7.5 Hz, 1H), 7.50 -7.46 (m, 1H), 7.19 (d, *J* = 7.5 Hz, 1H), 7.10 - 7.08 (m, 5H), 6.91 - 6.88 (m, 3H), 6.79 - 6.76 (m, 4H), 6.58 (d, *J* = 7.8 Hz, 1H), 6.39 (d, *J* = 6.9 Hz, 1H), 6.04 (s, 1H), 4.84 (dd, *J* = 6.6 Hz, 7.2 Hz, 1H), 4.55 (dd, *J* = 6.6 Hz, 7.5 Hz, 1H), 4.36 - 4.30 (m, 2H), 4.08 - 3.99 (m, 2H), 3.90 - 3.66 (m, 14H), 3.15 - 2.95 (m, 4H), 2.80 (d, *J* = 17.1 Hz, 1H), 2.61 - 2.46 (m, 2H), 2.39 - 2.32 (m, 1H), 2.04 (s, 1H), 1,41 (s, 9H), 1.31 (s, 9H), 1.26 (s, 9H), 0.82 - 0.78 (m, 9H). **^{13}C NMR** (75 MHz, $CDCl_3$) δ 173.61, 171.76, 171.71, 171.54, 170.82, 168.84, 167.99, 155.94, 154.35, 148.47, 147.77, 138.69, 136.55, 136.14, 131.42, 130.70, 129.66, 128.84, 128.42, 126.77, 124.25, 121.27, 117.04, 106.65, 105.05, 80.02, 78.38, 75.52, 65.95, 58.33, 57.33, 56.62, 55.76, 54.23, 53.78, 52.21, 51.92, 42.74, 40.19, 37.55, 37.44, 34.69, 28.75, 28.25, 28.04, 25.53, 24.85, 22.86, 21.53, 18.39, 16.82. **HRMS (ESI):** m/z $[M+H]^+$ calcd for $C_{62}H_{86}N_9O_{12}$: 1148.6390, found: 1148.6375.

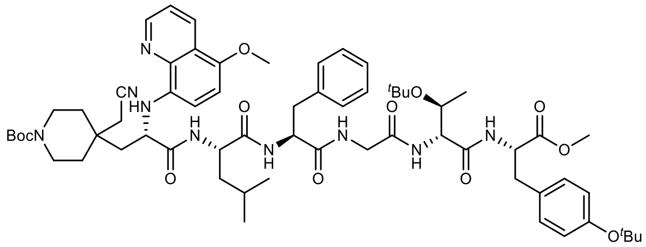

3of

***tert*-butyl 4-((4*S*,7*R*,13*S*,16*S*,19*S*)-13-benzyl-4-(4-(*tert*-butoxy)benzyl)-7-((*S*)-1-(*tert*-butoxy)ethyl)-16-isobutyl-19-((5-methoxyquinolin-8-yl)amino)-3,6,9,12,15,18-hexaoxo-2-oxa-5,8,11,14,17-pentaazaicosan-20-yl)-4-(cyanomethyl)piperidine-1-carboxylate (3of):** Yellow solid, M. p. 121 - 123 ºC. 76.5mg, 65% yield, d.r. > 20:1. $[\alpha]_D^{26.3}$ -2.00 (*c* 10.00 mg/mL, DCM). **^{1}H NMR** (300 MHz, $CDCl_3$) δ 8.84 (d, *J* = 3,6 Hz, 1H), 8.58 (d, *J* = 8.4 Hz, 1H), 7.58 (d, *J* = 7.5 Hz, 1H), 7.49 (q, *J* = 4.2 Hz, 1H), 7.15 - 7.07 (m, 6H), 6.89 (d, *J* = 7.8 Hz, 3H), 6.82 - 6.76 (m, 4H), 6.59 (d, *J* = 8.1 Hz, 1H), 6.31 (d, *J* = 7.8 Hz, 1H), 6.09 (s, 1H) , 4.85 (dd, *J* = 4.6 Hz, 12.9 Hz, 1H), 4.54 (dd, *J* = 8.1 Hz, 14.7 Hz, 1H), 4.35 - 4.31 (m, 2H), 4.12 -

3.79 (m, 8H), 3.66 (s, 3H), 3.52 - 3.35 (m, 4H), 3.13 - 3.09 (m, 2H), 2.99 (dd, J = 7.8 Hz, 14.1 Hz, 1H), 2.60 - 2.43 (m, 3H), 2.33 (d, J = 12.0 Hz, 1H), 2.05 - 1.97 (m, 2H), 1.63 - 1.59 (m, 4H), 1.43 - 1.39 (m, 10H), 1.31 (s, 9H), 1.23 (s, 9H), 0.83 - 0.76 (m, 9H). **^{13}C NMR** (75 MHz, $CDCl_3$) δ 174.53, 171.71, 171.62, 170.87, 168.80, 168.01, 154.60, 154.36, 148.48, 147.60, 136.58, 136.19, 131.41, 130.69, 129.66, 128.81, 128.41, 126.73, 124.25, 121.30, 117.43, 106.30, 105.16, 79.82, 78.38, 77.21, 75.53, 65.91, 57.33, 56.03, 55.78, 54.22, 53.77, 52.21, 52.11, 42.79, 40.68, 40.04, 37.58, 37.36, 34.44, 28.75, 28.34, 28.06, 26.27, 24.59, 22.80, 21.56, 16.79. **HRMS (ESI):** m/z $[M+H]^+$ calcd for $C_{64}H_{90}N_9O_{12}$: 1176.6703, found: 1176.6692.

2 Supplementary Discussion

2.1 Synthetic applications

2.1.1 Deprotection of 3ba

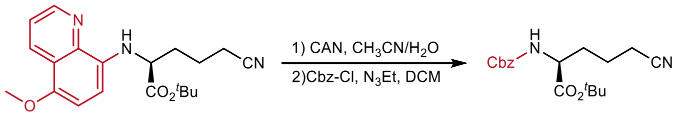

3ba, 93% ee

4, 75% yield, 90% ee

According to previous reports,[6] a mixture of **3ba** (0.1 mmol, 35.5 mg) and CAN $Ce(NH_4)_2(NO_3)_6$, 0.6 mmol, 328.9 mg) in 5:2 solution of H_2O/CH_3CN (1.0 mL) was stirred at 0 °C for 2 h. The mixture was modulated to alkalescence with saturated aqueous sodium carbonate. Then, the mixture was extracted by DCM for three times, washed with brine, dried over Na_2SO_4, and concentrated in vacuo. The residue was dissolved in 2 mL DCM. Benzyloxycarbonyl chloride (0.125 mmol, 0.018 mL) was added. The resulting solution was cooled to 0 °C and TEA (0.125 mmol, 0.017 mL) was then added dropwise. After 5 min, the ice bath was removed and the mixture was allowed to stir for 2 h at room temperature. 10 mL DCM was added and the mixture was washed with H_2O (10 mL) and brine (10 mL). The resulting solution was dried over Na_2SO_4 and evaporated in vacuo. The product **4** was purified by silica gel column chromatography using hexane-EtOAc as eluents. ***tert*-butyl (*S*)-2-**

(((benzyloxy)carbonyl)amino)-5-cyanopentanoate (4): brown oil. 24.9 mg, 75% yield. 90% ee determined by HPLC on a Chiralpak IA-H column (hexane/EtOH = 1/2, flow rate = 0.5 mL/min, detection at 211.8 nm, t_{minor} = 9.0 min, t_{major} = 11.2 min), $[\alpha]_D^{26.8}$ 6.00 (*c* 5.00 mg/mL, DCM). **^{1}H NMR** (300 MHz, $CDCl_3$) δ 7.35 (s, 5H), 5.44 (d, *J* = 7.5 Hz, 1H), 5.10 (s, 2H), 4.29 - 4.25 (m, 1H), 2.39 (t, *J* = 6.3 Hz, 2H), 2.02 - 1.95 (m, 1H), 1.78 - 1.76 (m, 3H), 1.47 (s, 9H). **^{13}C NMR** (75 MHz, $CDCl_3$) δ 170.74, 155.84, 136.07, 128.48, 128.17, 128.06, 119.07, 82.68, 66.95, 53.39, 31.88, 27.88, 21.27, 16.72. **HRMS (ESI):** m/z $[M+Na]^+$ calcd for $C_{18}H_{24}N_2NaO_4$: 355.1628, found: 355.1629.

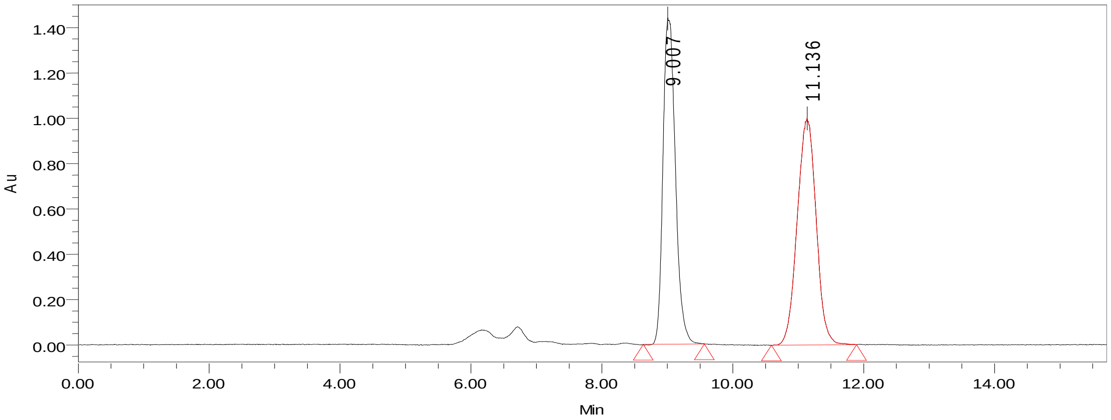

Entry	Retention time	Area	Area (%)	Height	Int type
1	9.007	19006291	48.69	1438886	bb
2	11.136	20028987	51.31	1000465	bb

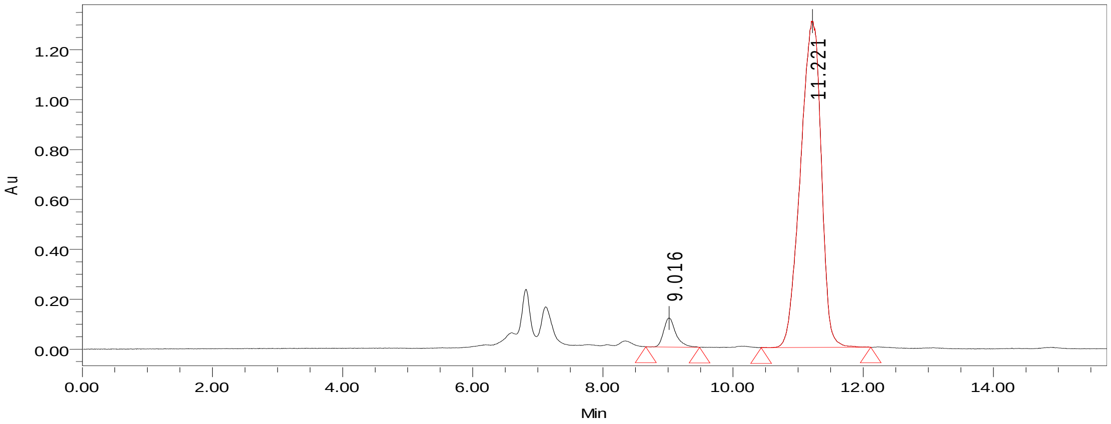

Entry	Retention time	Area	Area (%)	Height	Int type
1	9.016	1520935	4.99	117186	bb
2	11.221	28984885	95.01	1308245	bb

2.1.2 Conversion of cyano to amino

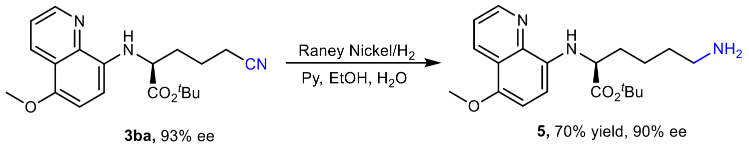

Wet Raney-Ni (0.15 g) was washed with milliQ water (15×1.0 mL) and suspended with **3ba** (35.5 mg, 0.1 mmol, 1 equiv.) in Py/EtOH/H_2O (1:1:1, 3.0 mL). The suspension was hydrogenated in a Parr apparatus (50 psi, 50 °C, 0.5 h). The mixture was filtered through dicalite and rinsed with water. After evaporation of the solvent, the residue was redissolved in EtOAc (10.0 mL) and washed with 1 M HCl (3 ×5.0 mL). The organic layer was dried ($MgSO_4$), filtered, and the solvents were evaporated. The product **5** was purified by silica gel column chromatography using hexane-EtOAc as eluents. ***tert*-butyl (5-methoxyquinolin-8-yl)-*L*-lysinate (5):** yellow oil. 25.2 mg, 70% yield. 90% ee determined by HPLC on a Chiralpak IA-H column (hexane/EtOH = 2/1, flow rate = 0.5 mL/min, detection at 261.8 nm, t_{minor} = 8.2 min, t_{major} = 9.2 min), $[\alpha]_D^{26.8}$ 2.00 (*c* 5.00 mg/mL, DCM). **^{1}H NMR** (300 MHz, $CDCl_3$) δ 8.79 (d, *J* = 3.9 Hz, 1H), 8.49 (d, *J* = 8.7 Hz, 1H), 7.39 (q, *J* = 4.2 Hz, 1H), 6.73 (d, *J* = 8.1 Hz, 1H) ,6.57 (d, *J* = 8.4 Hz, 1H), 4.09 (t, *J* = 6.6 Hz, 1H), 3.92 (s, 3H), 3.67 (t, *J* = 5.7 Hz, 2H), 2.04 - 1.89 (m, 2H), 1.68 - 1.60 (m, 6H), 1.38 (s, 9H). **^{13}C NMR** (75 MHz, $CDCl_3$) δ 173.44, 147.80, 146.17, 139.03, 138.02, 130.75, 121.19, 120.62, 105.27, 105.20, 81.19, 62.66, 57.60, 55.88, 32.63, 32.47, 27.99, 22.07.

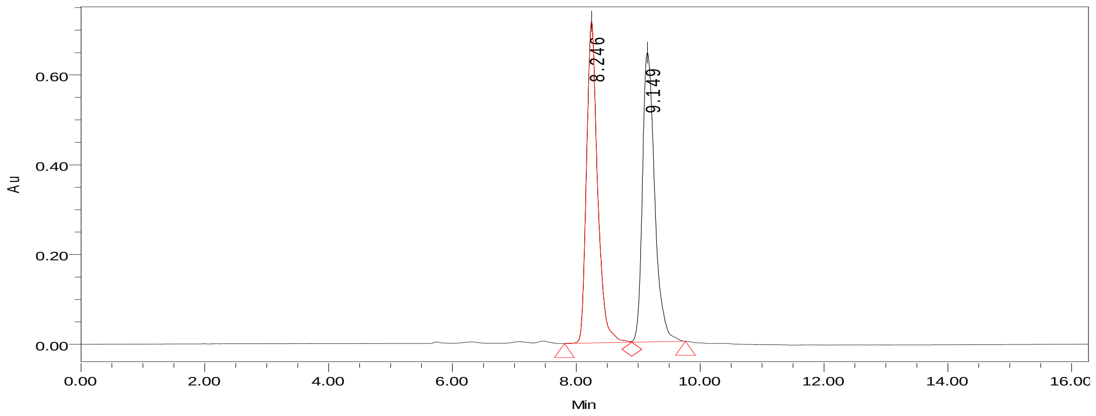

Entry	Retention time	Area	Area (%)	Height	Int type
1	8.246	2234547	49.78	178860	bv
2	9.148	2254423	50.22	160798	vb

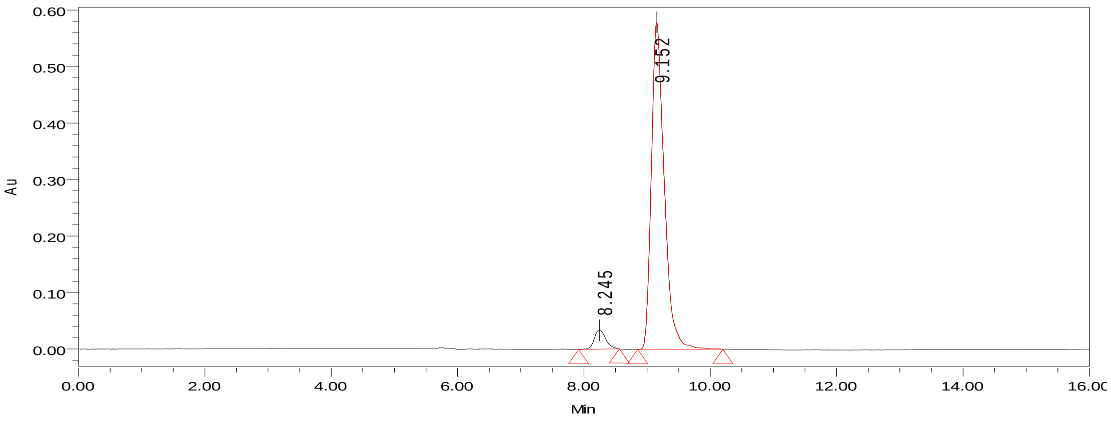

Entry	Retention time	Area	Area (%)	Height	Int type
1	8.246	410299	4.99	32120	bb
2	9.152	7817334	95.01	553549	bb

2.1.3 Conversion of cyano to amide

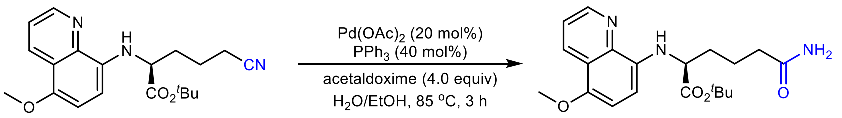

3ba, 93% ee **6**, 85% yield, 92% ee

According to previous reports,[4] a mixture of **3ba** (0.1 mmol, 35.5 mg), acetaldoxime (0.4 mmol, 24 μL), $Pd(OAc)_2$ (0.02 mmol, 4.5 mg), and PPh_3 (0.04 mmol, 10.5 mg) in aqueous EtOH ($EtOH/H_2O$ = 4/1, 1.5 mL) was heated to 85 °C in a sealed tube, the reaction was stirred for 3 h under argon atmosphere. The reaction mixture was filtered through a Celite pad and washed with EtOH/DCM. After removal of solvent and column chromatographic purification process (DCM/MeOH), get product **6**. ***tert*-butyl (*S*)-6-amino-2-((5-methoxyquinolin-8-yl)amino)-6-oxohexanoate (6):** yellow oil. 31.7 mg, 85% yield. 92% ee determined by HPLC on a Chiralpak IA-H column (hexane/EtOH = 1/4, flow rate = 0.5 mL/min, detection at 261.8 nm, t_{minor} = 7.8 min, t_{major} = 8.6 min), $[\alpha]_D^{26.8}$ -11.00 (*c* 10.00 mg/mL, DCM). **^{1}H NMR** (300 MHz, $CDCl_3$) δ 8.79 - 8.77 (m, 1H), 8.49 (d, *J* = 8.4 Hz, 1H), 7.39 (q, *J* = 3.9 Hz, 1H), 6.73 (d, *J* = 8.4 Hz, 1H), 6.57 (d, *J* = 8.4 Hz, 1H), 6.16 (d, *J* = 7.5 Hz, 1H), 5.67 (s, 1H), 5.47 (s,

1H), 4.10 - 4.09 (m, 1H), 3.92 (s, 3H), 2.32 (t, J = 6.9 Hz, 2H), 2.05 - 1.89 (m, 4H), 1.39 (s, 9H). **^{13}C NMR** (75 MHz, $CDCl_3$) δ 174.79, 173.16, 147.84, 146.26, 138.99, 137.81, 130.79, 121.19, 120.66, 105.26, 105.20, 81.43, 57.41, 55.87, 35.37, 32.10, 27.97, 22.03. **HRMS (ESI):** m/z $[M+H]^+$ calcd for $C_{20}H_{28}N_3O_4$: 374.2074, found: 374.2076.

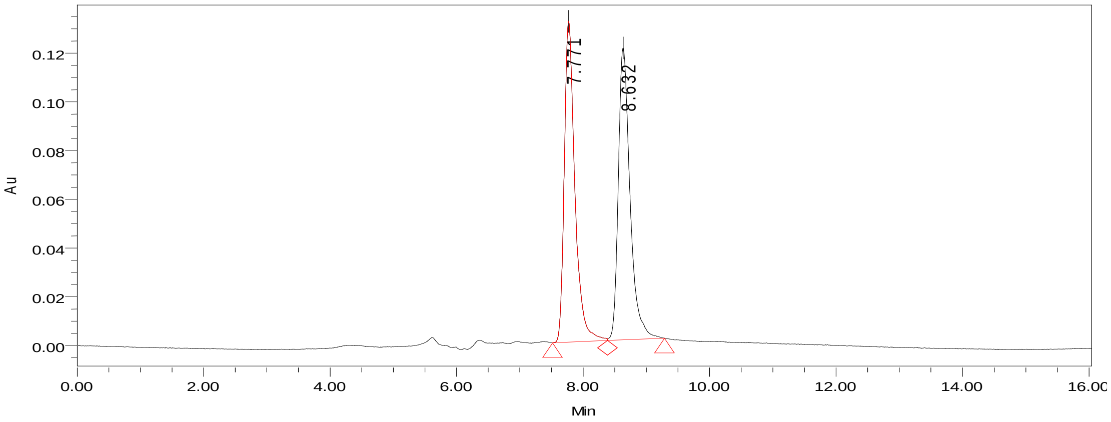

Entry	Retention time	Area	Area (%)	Height	Int type
1	7.771	1542491	49.92	131647	bv
2	8.632	1547210	50.08	119820	vb

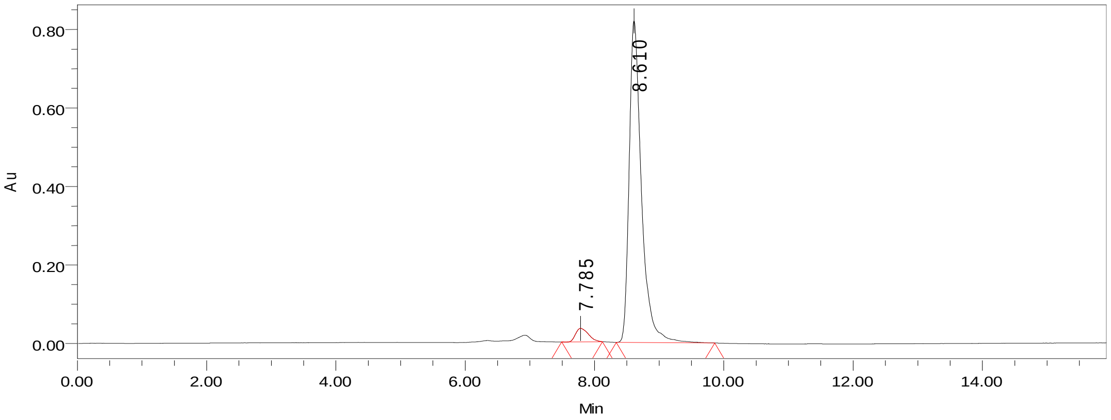

Entry	Retention time	Area	Area (%)	Height	Int type
1	7.785	471035	4.16	34601	bb
2	8.610	10850156	95.84	818938	bb

2.2 The mechanistic studies

2.2.1 Radical trapping experiments

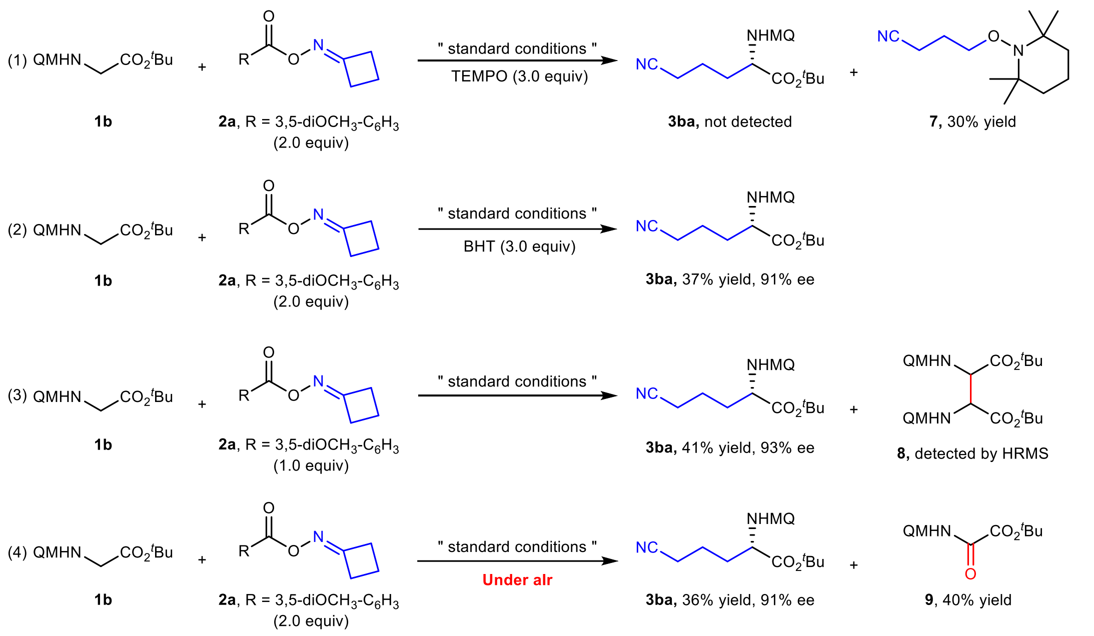

Supplementary Fig. 3. Radical trapping experiments.

The reaction between **1b** and **2a** was set up according to the procedures of standard conditions, while in the presence of TEMPO ((2,2,6,6-tetramethylpiperidin-1-yl)oxyl) (0.3 mmol, 46.9 mg). The desired product **3ba** was not detected by TLC, whereas **7** was obtained in 30% yield (Supplementary Fig. 3, equation 1). **4-((2,2,6,6-tetramethylpiperidin-1-yl)oxy)butanenitrile (7):** Yellow oil, 6.7 mg, 30% yield. **^{1}H NMR** (300 MHz, $CDCl_3$) δ 3.84 (t, J = 5.7 Hz, 2H), 2.49 (t, J = 7.2 Hz, 2H), 1.91 - 1.87 (m, 2H), 1.48 - 1.43 (m, 5H), 1.32 (d, J = 11.4 Hz, 1H), 1.15 (s, 6H), 1.09 (s, 6H). **^{13}C NMR** (75 MHz, $CDCl_3$) δ 119.26, 73.16, 59.36, 39.13, 32.64, 24.67, 19.62, 16.60, 14.04. **HRMS (ESI):** m/z $[M+ Na]^+$ calcd for $C_{13}H_{25}N_2NaO$: 247.1781, found: 247.1768. On the other hand, in the presence of BHT (butylated hydroxytoluene, 0.3 mmol, 60.1 mg), the desired product **3ba** was obtained in 37% yield and 91% ee (Supplementary Fig. 3, equation 2). These results indicated that cyanoalkyl radical was formed in the reaction process.

When the feeding amount of **2a** is 1.0 equivalent (Supplementary Fig. 3, equation 3),

the HRMS analysis of the original reaction mixture indicated the formation of glycinate homo-coupling product **8** (Supplementary Fig. 4). **HRMS (ESI):** m/z $[M+H]^+$calcd for $C_{32}H_{39}N_4O_6$: 575.2864, found: 575.2835.

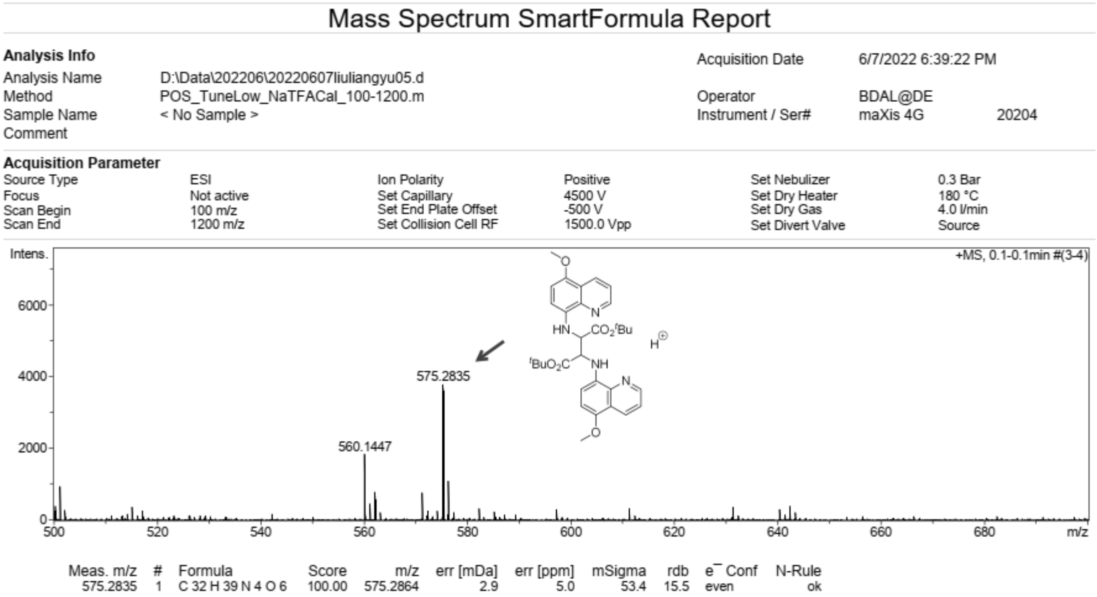

Supplementary Fig. 4. HRMS analysis spectrum of glycine homologous coupling product **8**.

Meanwhile, when the reactions of **1b** and **2a** were carried out under air atmosphere, 40% of oxidized glycine derivatives **9** was formed (Supplementary Fig. 3, equation 4). All these results indicated that glycinate radical were involved in catalytic cycle. ***tert*-butyl 2-((5-methoxyquinolin-8-yl)amino)-2-oxoacetate (9):** Yellow oil, 12.1 mg, 40% yield. **^{1}H NMR** (300 MHz, $CDCl_3$) δ 11.18 (s, 1H), 8.89 - 8.87 (m, 1H), 8.72 (d, J = 8.4 Hz, 1H), 8.58 (d, J = 8.4 Hz, 1H), 7.46 (q, J = 4.2 Hz, 1H), 6.87 (d, J = 8.4 Hz, 1H), 4.01 (s, 3H), 1.65 (s, 9H). **^{13}C NMR** (75 MHz, $CDCl_3$) δ 159.85, 154.66, 151.34, 149.24, 139.47, 131.15, 126.59, 120.96, 120.43, 117.35, 104.08, 84.58, 55.74, 27.76. **HRMS (ESI):** m/z $[M+ H]^+$ calcd for $C_{16}H_{19}N_2O_4$: 303.1339, found: 303.1337.

2.2.2 Control experiments

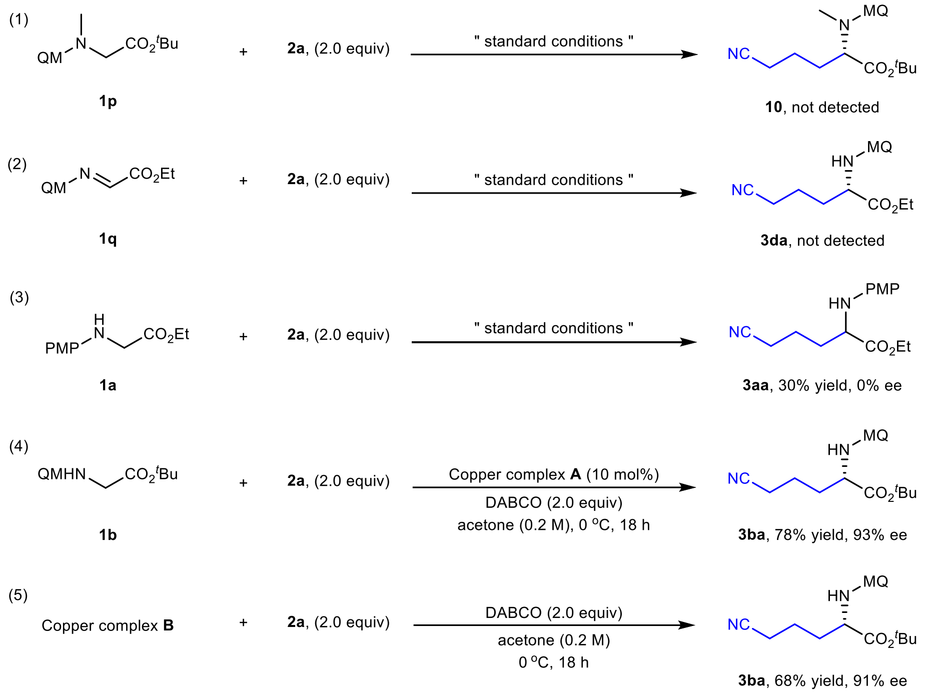

Supplementary Fig. 5. Control experiments.

Notes: Firstly, the *N*-CH_3-substituted glycinate **(1p)** did not give any cyanoalkylation product with quantitive recycling of the starting material, the results indicated that a single free hydrogen atom on *N* atom is crucial for this reaction (Supplementary Fig. 5, equation 1). Secondly, imine **(1q)** failed to give the cyanoalkylation product, revealing the Cu-mediated SET pathway likely involved rather than the two electron oxidation process (Supplementary Fig. 5, equation 2). Meanwhile, in equation 3, ethyl (4-methoxyphenyl) glycinate (**1a**) provided racemic product under standard conditions (30% yield, 0% ee). **ethyl 5-cyano-2-((4-methoxyphenyl)amino)pentanoate (3aa):** yellow solid, M. p. 150 - 152 °C. 8.3 mg, 30% yield. 0% ee determined by HPLC on a Chiralpak IA-H column (hexane/EtOH = 4/1, flow rate = 0.5 mL/min, detection at 241.5 nm, t_1 = 33.9 min, t_2 = 36.8 min), **^{1}H NMR** (300 MHz, $CDCl_3$) 6.77 (d, *J* = 7.8 Hz, 2H), 6.60 (d, *J* = 7.8 Hz, 2H), 4.18 (q, *J* = 6.9 Hz, 2H), δ 3.98 - 3.89 (m, 2H), 3.73 (s, 3H), 2.40 (t, *J* = 6.0 Hz, 2H), 2.00 - 1.96 (m, 1H), 1.91 - 1.80 (m, 3H), 1.24 (t, *J* = 6.9 Hz, 3H), **^{13}C NMR** (75 MHz, $CDCl_3$) δ 173.59, 152.90, 140.52, 119.13, 115.26, 114.82, 61.25, 57.13,

55.60, 31.78, 21.80, 16.91, 14.14. **HRMS (ESI):** m/z $[M+H]^+$calcd for $C_{15}H_{21}N_2O_3$: 277.1547, found: 277.1544.

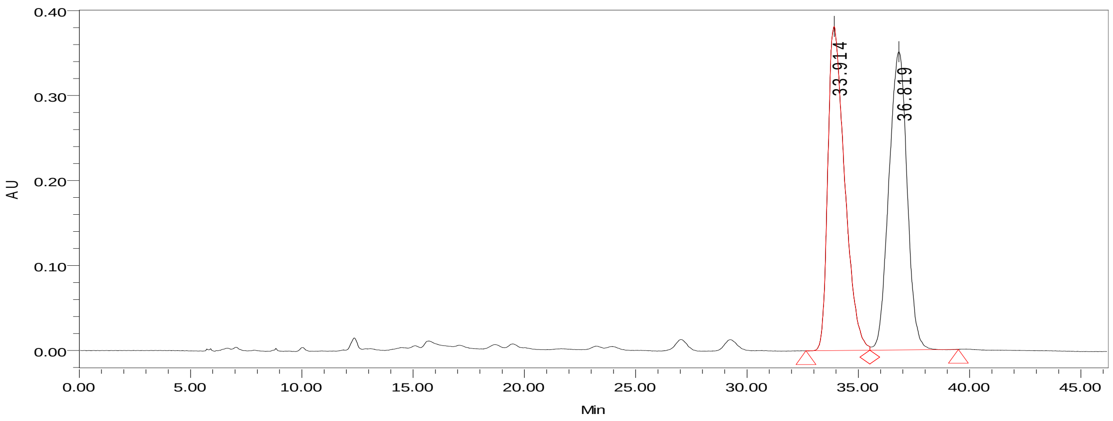

Entry	Retention time	Area	Area (%)	Height	Int type
1	33.914	20030151	50.09	381343	bv
2	36.819	19956439	49.91	350857	vb

In equation 4, the isolated Cu complex **A** was used as the catalyst, the corresponding C-C coupling products were obtained in 78% yield and 93% ee. In equation 5, the isolated Cu complex **B** (1 equiv) was directly used as the substrate without any other catalyst in the system, the coupling product was obtained in 68% yield and 91% ee. The results from equations 3-5 indicated that complex **B** might possibly form in the reaction and act as the active catalytic species.

2.2.3 Characteristics of copper complex

Note: The synthesis of the complex of $Cu(CH_3CN)_4PF_6$, **1b** and (*S*)-An-Phanephos. The complex was prepared according to the reported method.[7]

2.2.3.1 General procedure for the synthesis of copper complex **A**

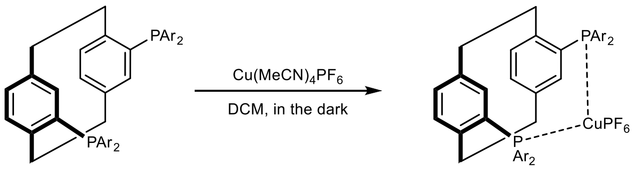

L2: (*S*)-An-Phanephos
Ar = 4-OMe-phenyl

copper complex A

In glovebox, to an schlenk tubes with stirring bar charged $Cu(MeCN)_4PF_6$ (0.1 mmol,

37.3 mg) and (*S*)-An-Phanephos (0.1 mmol, 69.7 mg), respectively, followed by the addition of DCM (2 mL). The mixture was stirred at room temperature for 2 hours in the dark. When reaction completed, the reaction mixture was concentrated to a fifth of the original volume and *n*-pentane was added dropwise to precipitate the white solid. The white solid was washed with *n*-pentane and dried to obtain copper complex **A**. **^{1}H NMR** (300 MHz, $CDCl_3$) δ 7.58 - 7.55 (m, 4H), 7.30 - 7.27 (m, 4H), 7.00 - 6.94 (m, 10H), 6.62 - 6.55 (m, 4H), 3.85 (s, 6H), 3.81 (s, 6H), 2.68 - 2.62 (m, 6H), 2.30 - 2.27 (m, 2H). **^{13}C NMP** (75 MHz, $CDCl_3$) 162.02, 161.03, 142.93, 139.21, 136.12, 135.97, 135.84, 135.81, 135.50, 134.91, 134.79, 134.66, 125.90, 123.53, 123.29, 123.03, 120.14, 114.80, 114.67, 114.58, 55.26, 53.43, 35.16, 33.17. **^{31}P NMR** (121.5 MHz, $CDCl_3$) δ 0.67, -138.38, -144.24, -150.11. **MS (ESI):** m/z $[M]^+$ calcd for $C_{44}H_{42}CuO_4P_2$: 759.1849, found: 759.1799.

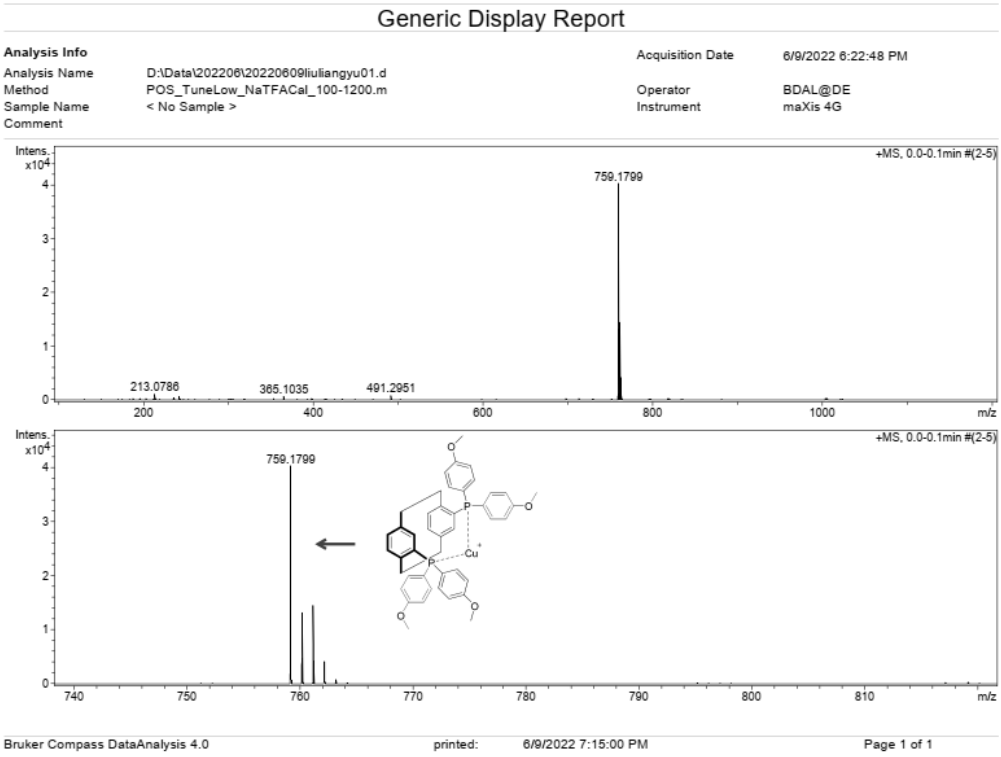

Supplementary Fig. 6. MS analysis of copper complex **A.**

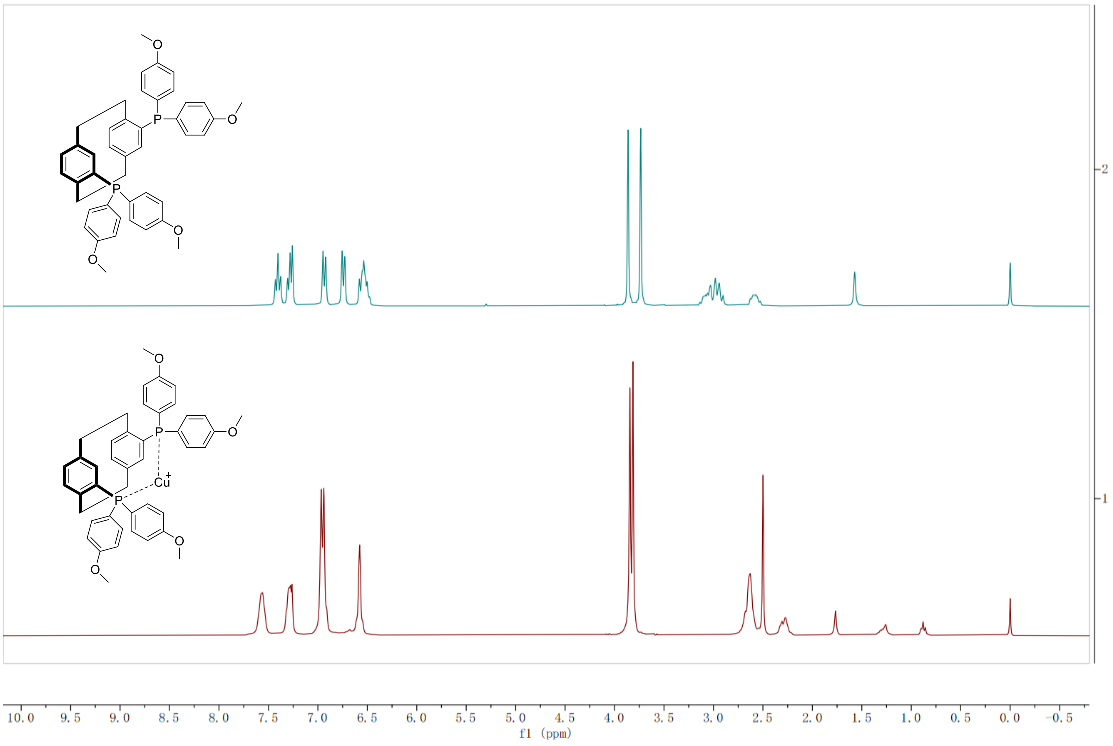

Supplementary Fig. 7. ^{1}H NMR analysis of copper complex **A** and (*S*)-An-Phanephos**.**

2.2.3.2 General procedure for the synthesis of copper complex **B**

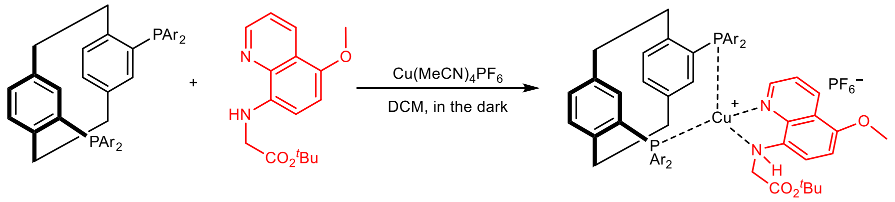

L2: (*S*)-An-Phanephos
Ar = 4-OMe-phenyl

1b

copper complex B

In glovebox, to an schlenk tubes with stirring bar charged $Cu(MeCN)_4PF_6$ (0.1 mmol, 37.3 mg) and 5-methoxyquinolinyl-8-glycinate ester **1b** (0.1 mmol, 28.8 mg), respectively, followed by the addition of DCM (2 mL). The mixture was stirred at room temperature for 10 min in the dark. Subsequently, (*S*)-An-Phanephos (0.1 mmol, 69.7 mg) dissolved in DCM (2 mL) were added to above mentioned mixed solution by syringe, and the mixture was stirred for another 2 hours in the dark. When the reaction completed, the reaction mixture was concentrated to a fifth of the original volume and *n*-pentane was added dropwise to precipitate the yellow solid. The yellow solid was washed with *n*-pentane and dried to obtain copper complex **B**. **^{1}H NMR** (300 MHz,

$CDCl_3$). δ 8.86 (s, 1H), 8.75 (d, *J* = 7.8 Hz, 1H), 7.68 (brs, 1H), 7.42 (brs, 5H), 7.26 - 7.23 (m, 2H), 7.00 (d, *J* = 7.8 Hz, 1H), 6.87 (d, *J* = 6.3 Hz, 7H), 6.72 (d, *J* = 7.2 Hz, 5H), 6.59 (brs, 4H), 5.22 (brs, 1H), 4.05 (s, 3H), 3.83 (s, 6H), 3.77 (brs, 8H), 2.70 (brs, 6H), 2.34 (brs, 2H), 1.38 (s ,9H). **^{13}C NMR** (75 MHz, $CDCl_3$) 170.12, 161.89, 160.90, 160.66, 150.40, 142.87, 139.16, 139.08, 138.99, 136.45, 136.30, 136.14, 135.83, 135.68, 135.57, 135.45, 134.57, 133.91, 133.84, 132.84, 132.80, 126.15, 125.92, 124.22, 123.99, 122.00, 121.63, 114.71, 114.39, 105.44, 83.25, 56.13, 55.28, 55.18, 35.00, 33.44, 27.66. **^{31}P NMR** (121.5 MHz, $CDCl_3$) δ 0.17, -138.35, -144.21, -150.08. **MS(ESI)**: m/z $[M]^+$ calcd for $C_{60}H_{62}CuN_2O_7P_2$: 1047.3323, found: 1047.3196.

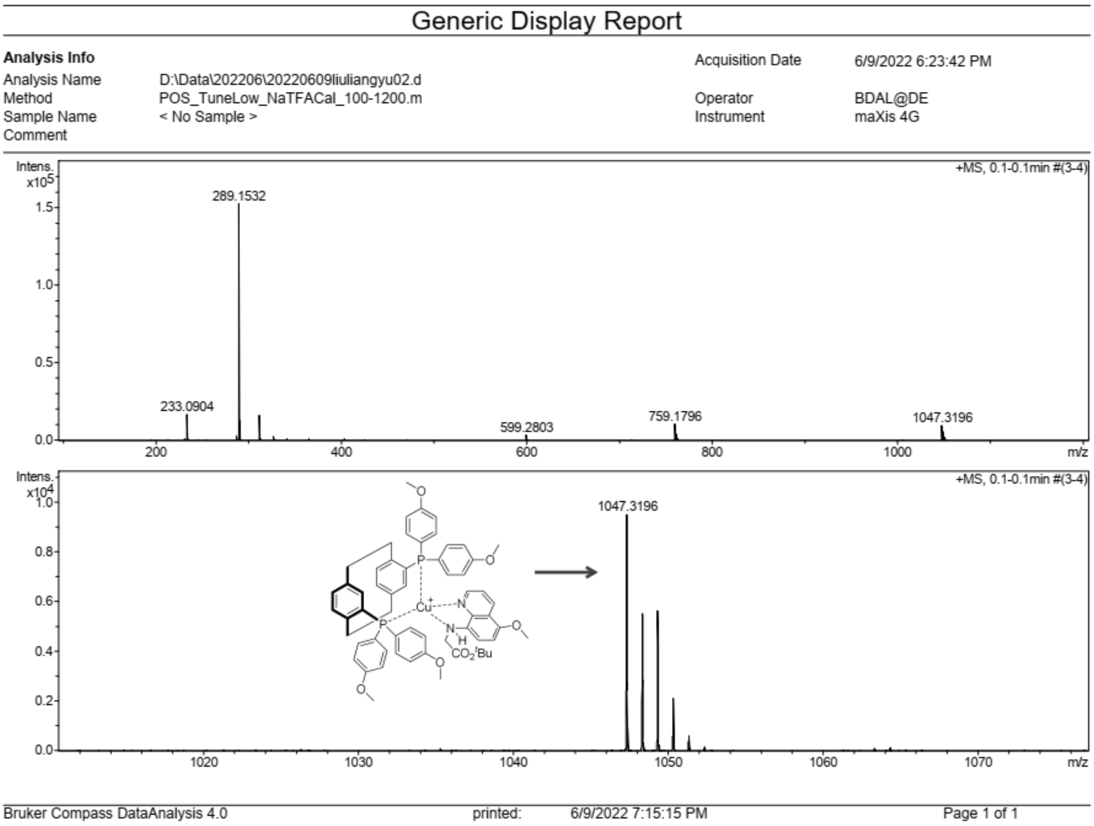

Supplementary Fig. 8. MS analysis of copper complex **B.**

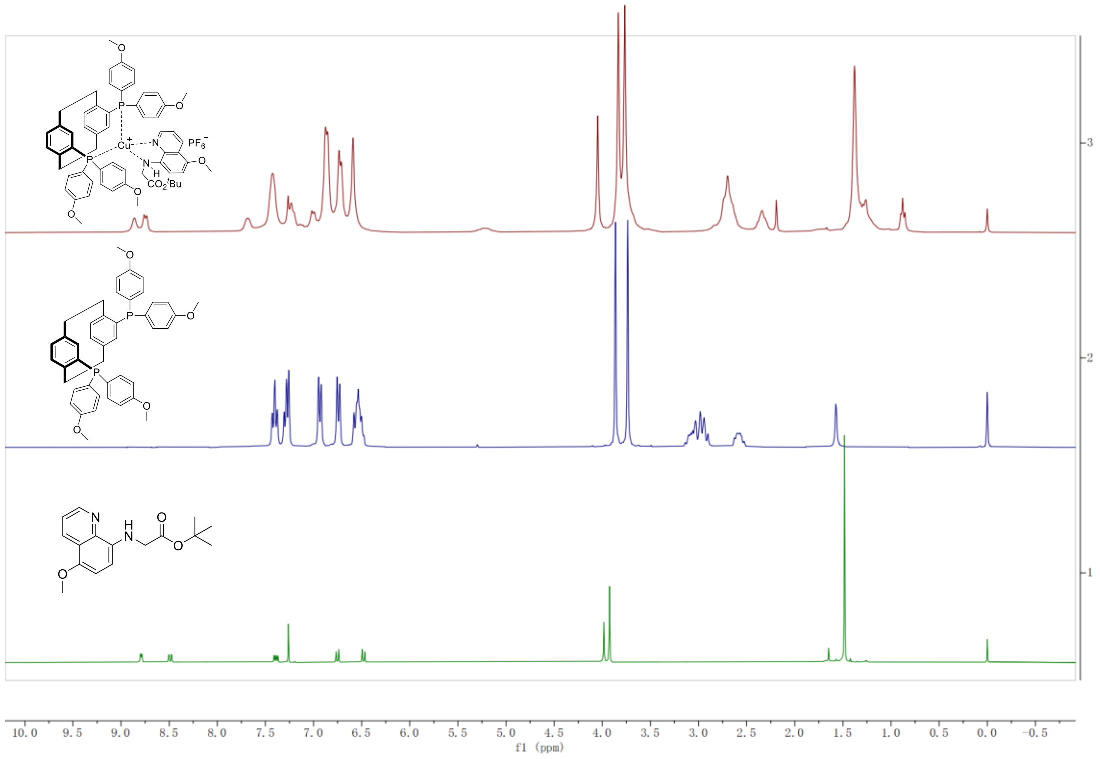

Supplementary Fig. 9. ^{1}H NMR analysis of copper complex **B**, (*S*)-An-Phanephos and **1b.**

2.2.4 Cyclic Voltammetry experiments

Cyclic Voltammetry was performed on a CH Instruments Electrochemical Workstation model CHI760E. A solution of the sample in MeCN (0.001 M) was tested with 0.1 M Bu_4NPF_6 as the supporting electrolyte, using a glassy carbon as the working electrode, a Pt as the counter electrode, and a saturated calomel electrode reference electrode. Scan rate = 0.05 V/s, 2 sweep segments, asample interval of 0.001 V.

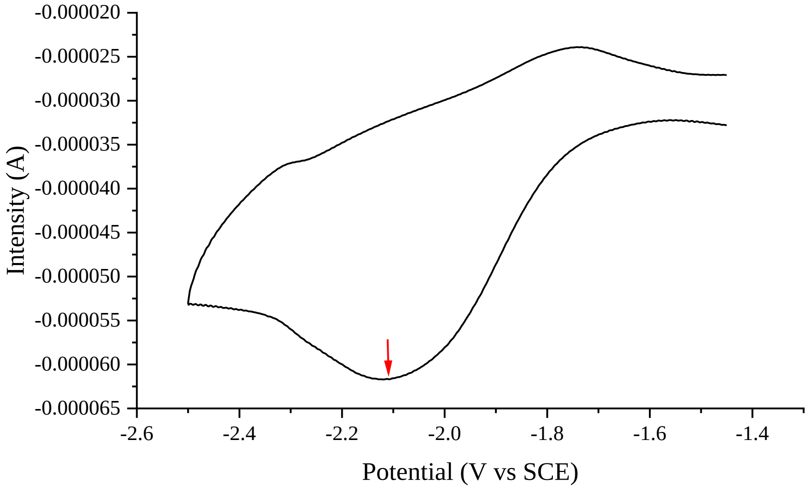

Supplementary Fig. 10. Cyclic Voltammetry of **2a**.

$E_{redox}^{0/-1}$ (**2a**) = -2.11 V (vs SCE)

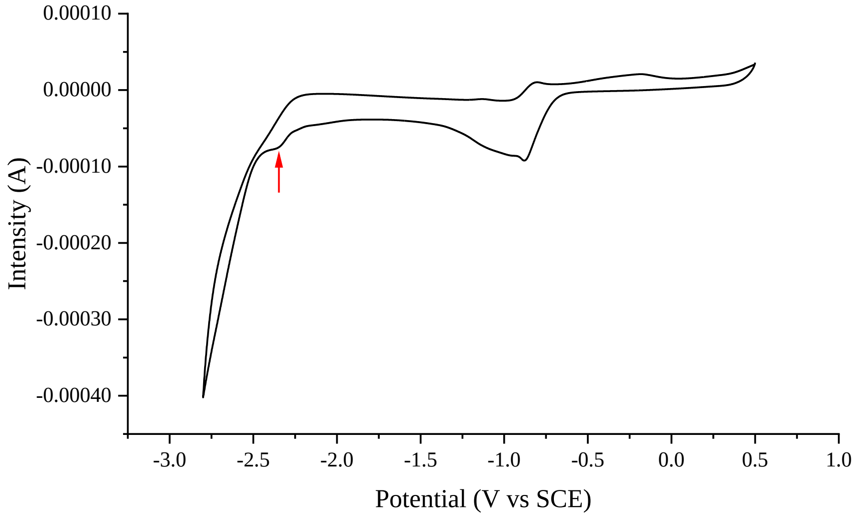

Supplementary Fig. 11. Cyclic Voltammetry of copper complex **B**.

$E_{P/2}^{red}(Cu^{II}/Cu^{I})$ = -2.38 V (vs SCE)

2.3 X-ray crystallography

2.3.1 Experimental

Single crystals of $C_{20}H_{25}N_3O_3$ were **3ba**. A suitable crystal was selected and **3ba** on a ROD, Synergy Custom system, HyPix diffractometer. The crystal was kept at 150.00(10) K during data collection. Using Olex2,[8] the structure was solved with the ShelXT[9] structure solution program using Intrinsic Phasing and refined with the ShelXL[10] refinement package using Least Squares minimisation.

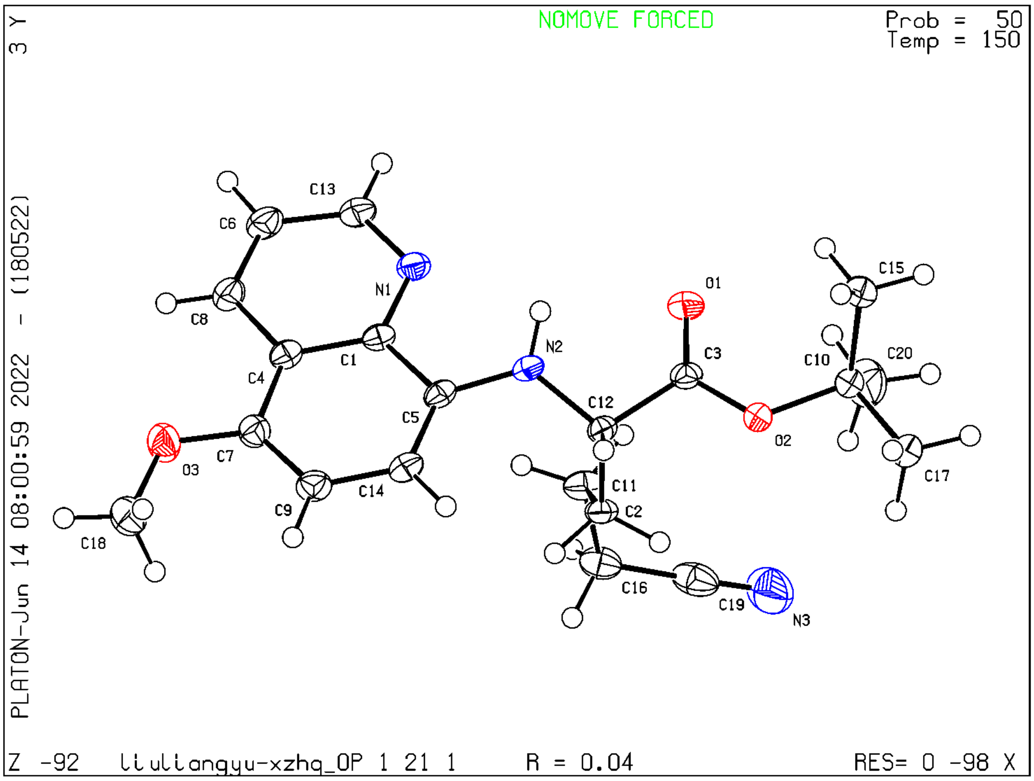

Supplementary Fig. 12. X-ray crystallography of **3ba**.

2.3.2 Crystal structure determination of 3ba

Crystal Data for $C_{20}H_{25}N_3O_3$ (M = 355.43 g/mol): monoclinic, space group $P2_1$ (no. 4), a = 6.22470(10) Å, b = 10.66480(10) Å, c = 14.37920(10) Å, β = 95.8220(10) °, V = 949.642(19) Å^3, Z = 2, T = 150.00(10) K, μ(Cu Kα) = 0.683 mm^{-1}, $Dcalc$ = 1.243 g/cm^3, 11775 reflections measured (6.178° ≤ 2Θ ≤ 153.76°), 3790 unique (R_{int} = 0.0366,

R_{sigma} = 0.0273) which were used in all calculations. The final R_1 was 0.0351 (I > 2σ(I)) and wR_2 was 0.0920 (all data).

Supplementary Table 13. Crystal data and structure refinement for CCDC 2178934

Compound	**3ba**
Empirical formula	$C_{20}H_{25}N_3O_3$
CCDC Number	2178934
Formula weight	355.43
Temperature/K	150.00(10)
Crystal system	monoclinic
Space group	$P2_1$
a/Å	6.22470(10)
b/Å	10.66480(10)
c/Å	14.37920(10)
α/°	90
β/°	95.8220(10)
γ/°	90
Volume/Å^3	949.642(19)
Z	2
ρ_{calc}g/cm^3	1.243
μ/mm^{-1}	0.683
F(000)	380.0
Crystal size/mm^3	0.09 × 0.07 × 0.05
Radiation	Cu Kα (λ = 1.54184)
2Θ range for data collection/°	6.178 to 153.76
Index ranges	-7 ≤ h ≤ 5, -13 ≤ k ≤ 12, -18 ≤ l ≤ 17
Reflections collected	11775
Independent reflections	3790 [R_{int} = 0.0366, R_{sigma} = 0.0273]
Data/restraints/parameters	3790/1/239

Goodness-of-fit on F^2	1.051
Final R indexes [I>=2σ (I)]	R_1 = 0.0351, wR_2 = 0.0916
Final R indexes [all data]	R_1 = 0.0356, wR_2 = 0.0920
Largest diff. peak/hole / e $Å^{-3}$	0.27/-0.34
Flack parameter	0.04(7)

2.4 NMR spectra

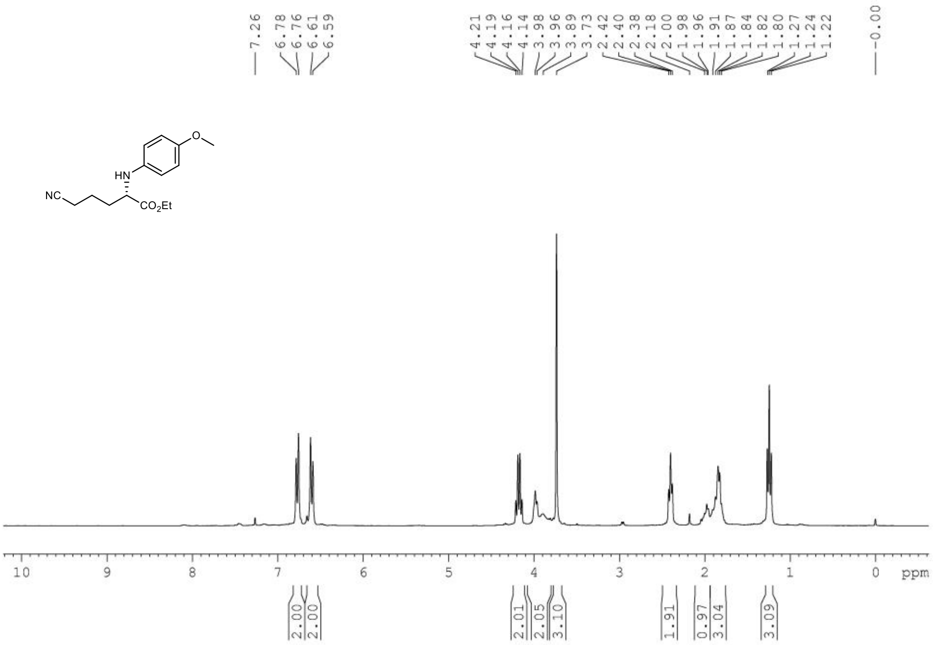

Supplementary Fig. 13. ^{1}H NMR of compound **3aa** (300 MHz, $CDCl_3$)

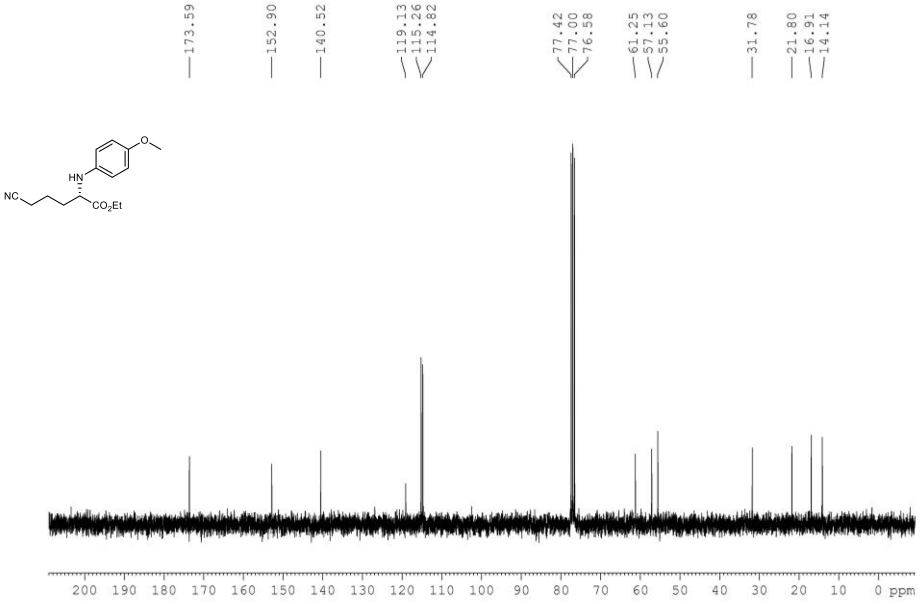

Supplementary Fig. 14. ^{13}C NMR of compound **3aa** (75 MHz, $CDCl_3$)

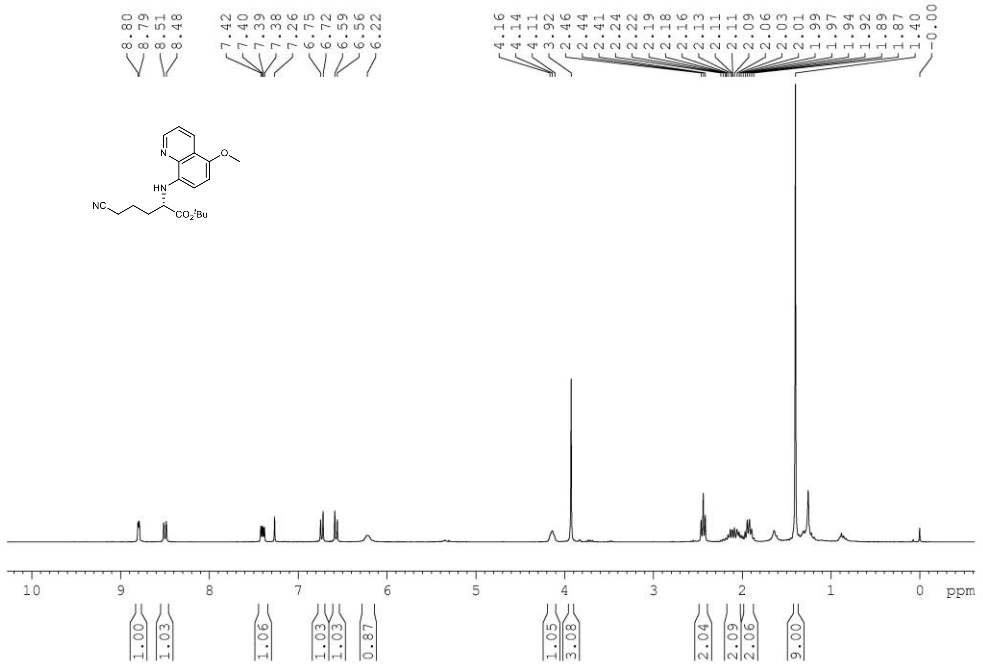

Supplementary Fig. 15. ^{1}H NMR of compound **3ba** (300 MHz, $CDCl_3$)

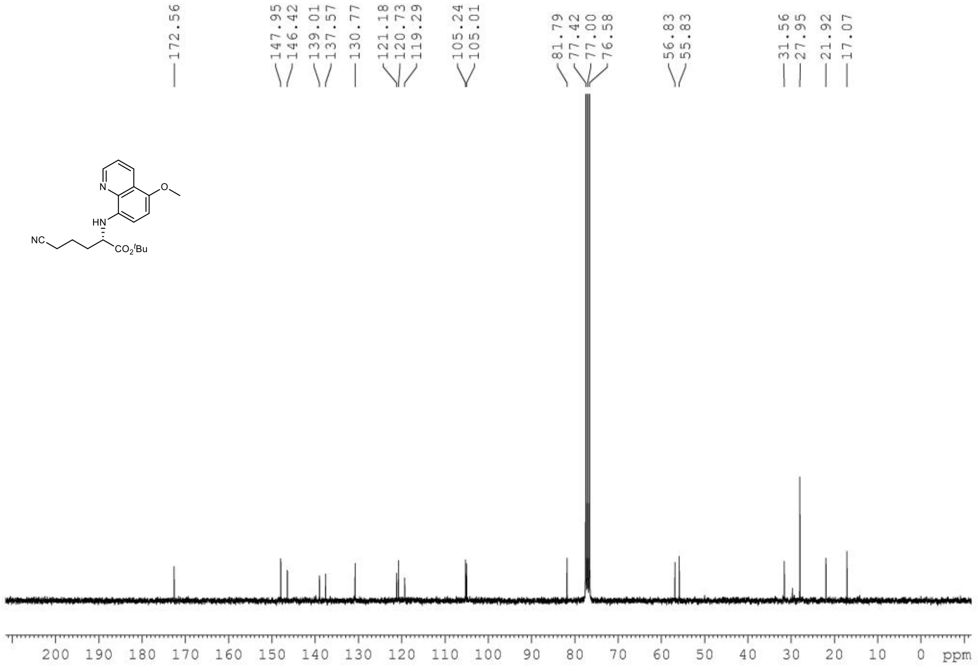

Supplementary Fig. 16. ^{13}C NMR of compound **3ba** (75 MHz, $CDCl_3$)

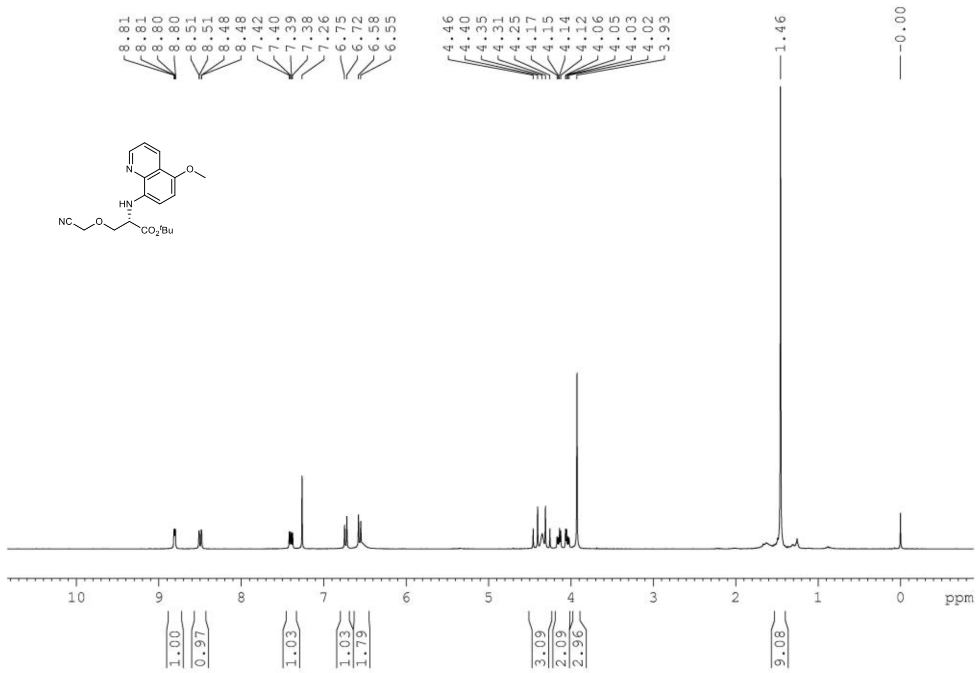

Supplementary Fig. 17. ^{1}H NMR of compound **3bb** (300 MHz, $CDCl_3$)

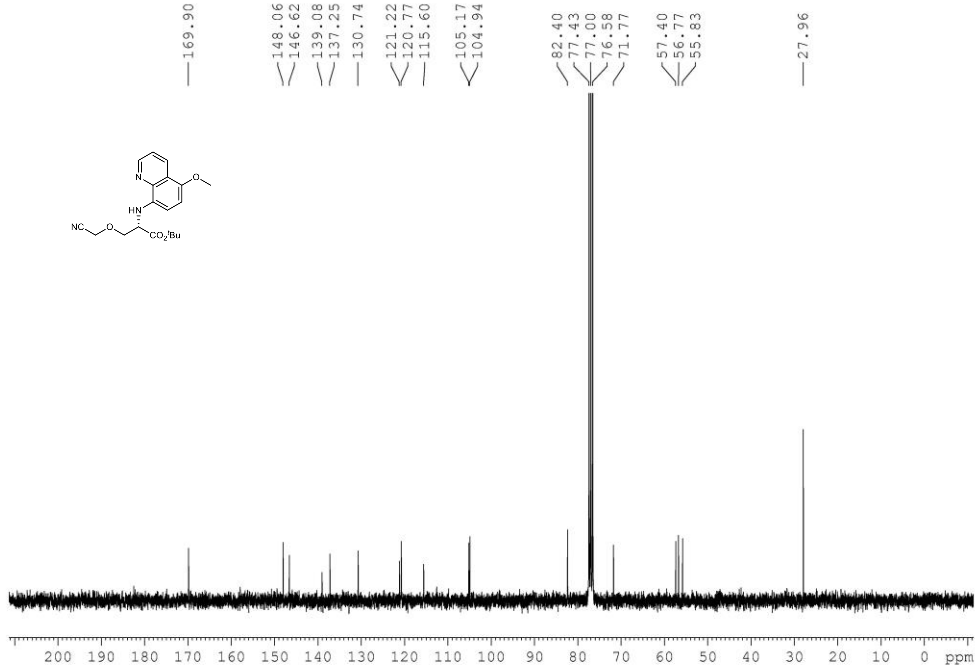

Supplementary Fig. 18. ^{13}C NMR of compound **3bb** (75 MHz, $CDCl_3$)

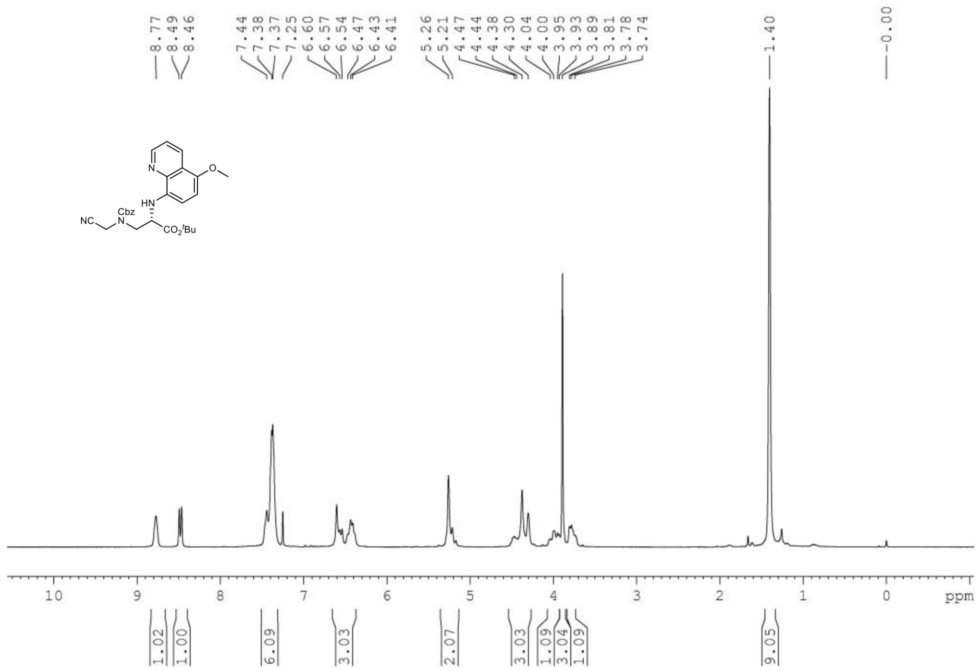

Supplementary Fig. 19. ^{1}H NMR of compound **3bc** (300 MHz, $CDCl_3$)

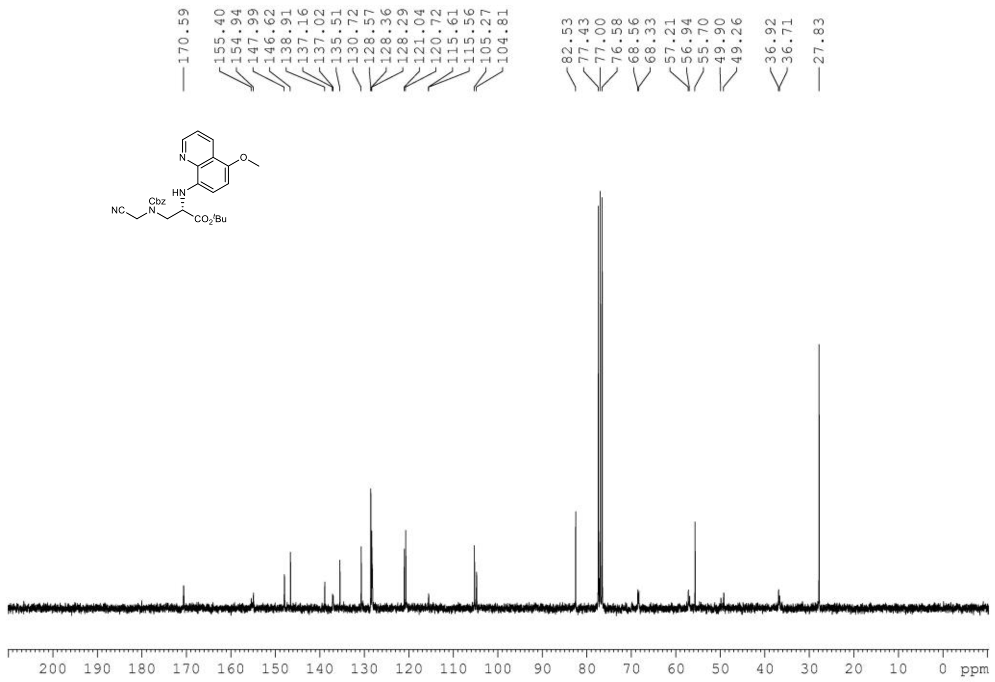

Supplementary Fig. 20. ^{13}C NMR of compound **3bc** (75 MHz, $CDCl_3$)

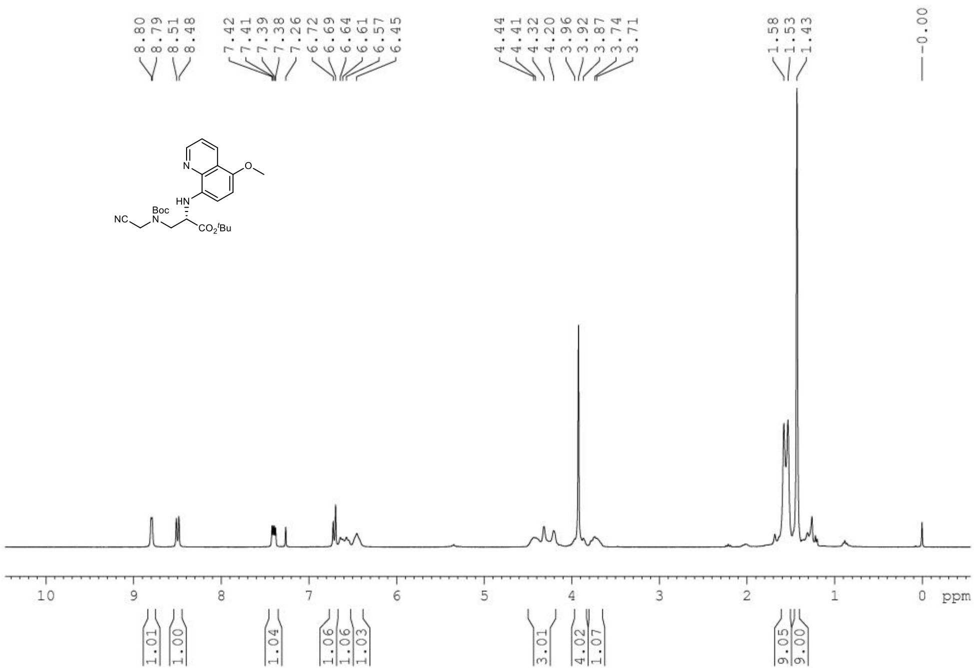

Supplementary Fig. 21. ^{1}H NMR of compound **3bd** (300 MHz, $CDCl_3$)

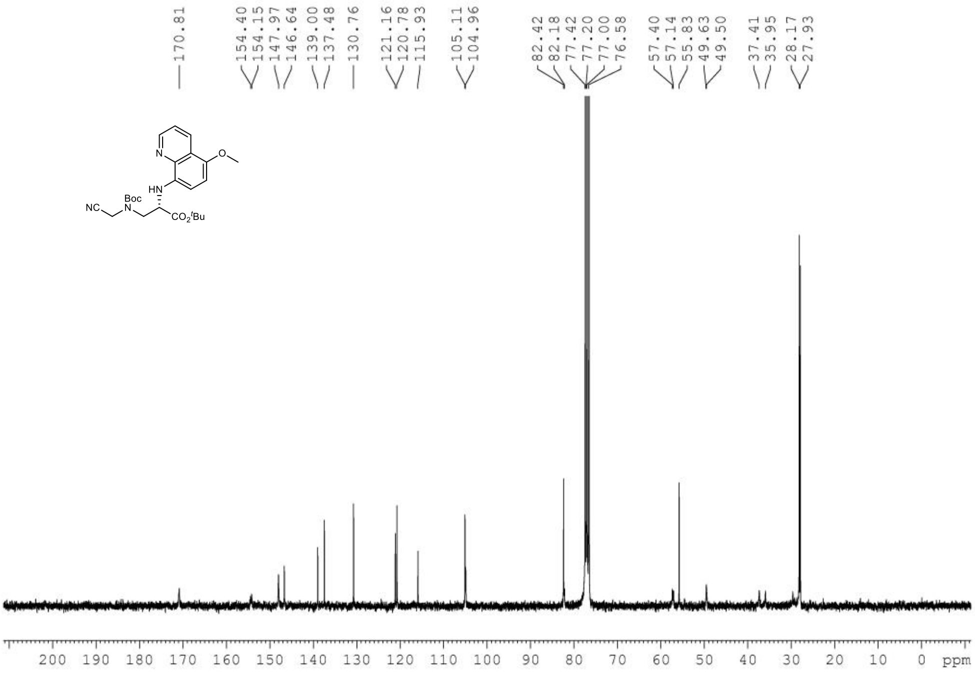

Supplementary Fig. 22. ^{13}C NMR of compound **3bd** (75 MHz, $CDCl_3$)

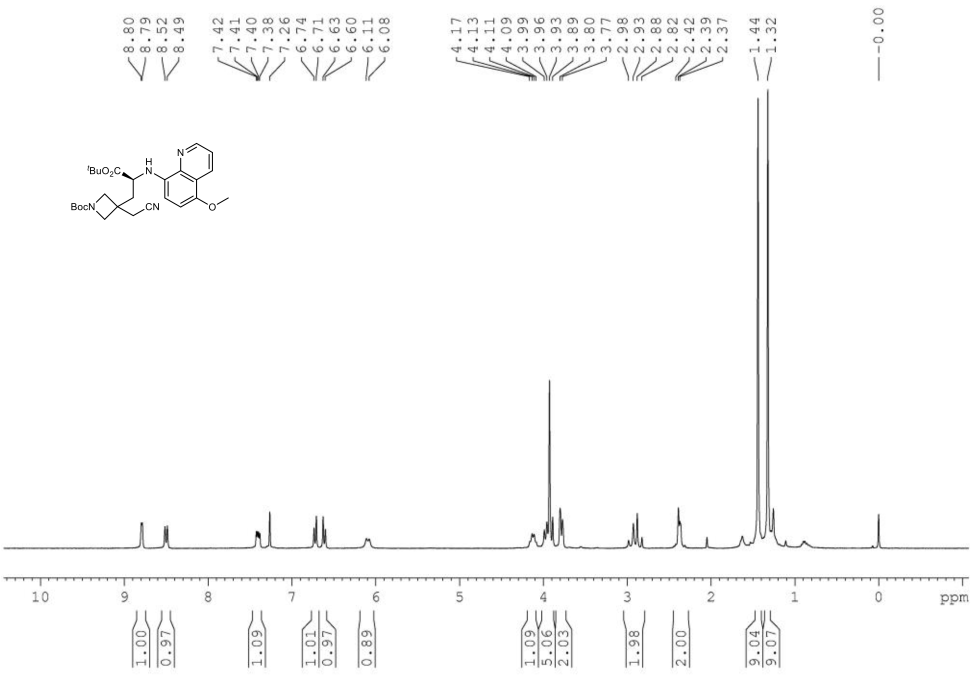

Supplementary Fig. 23. ^{1}H NMR of compound **3be** (300 MHz, $CDCl_3$)

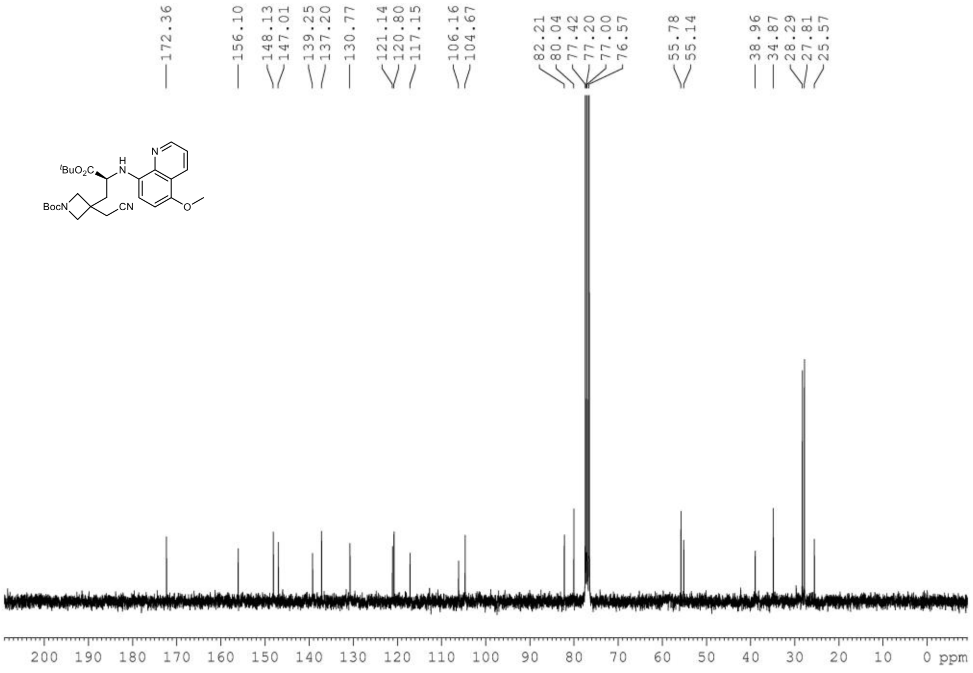

Supplementary Fig. 24. ^{13}C NMR of compound **3be** (75 MHz, $CDCl_3$)

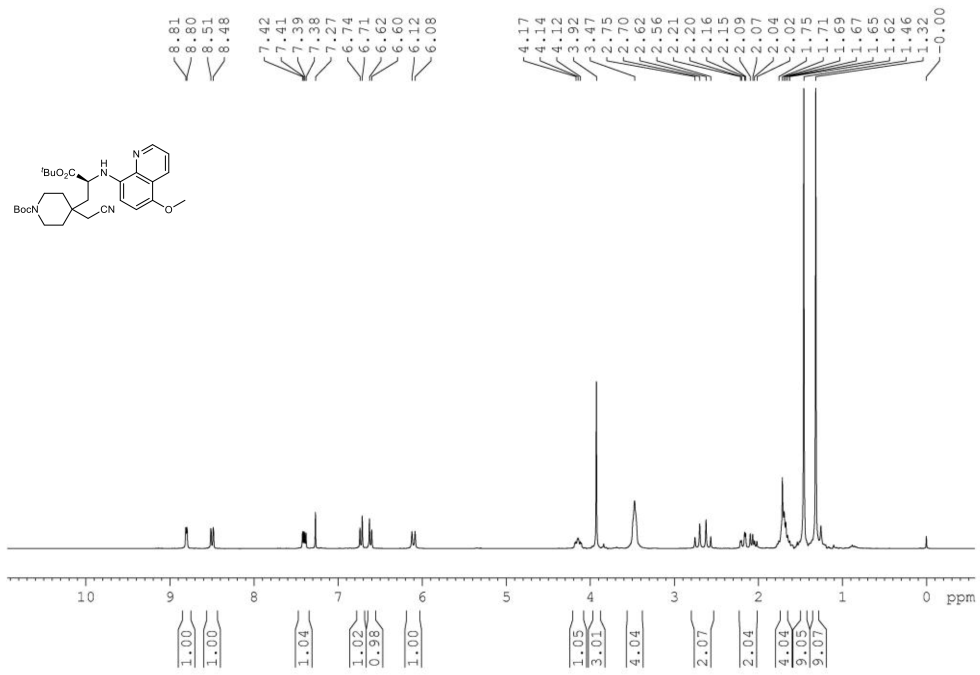

Supplementary Fig. 25. ^{1}H NMR of compound **3bf** (300 MHz, $CDCl_3$)

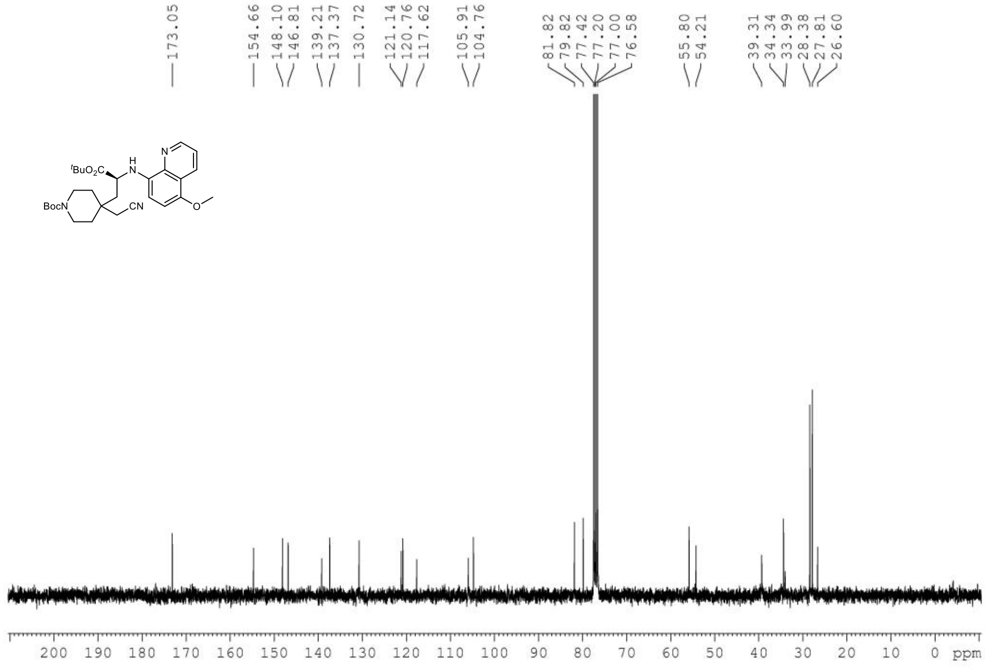

Supplementary Fig. 26. ^{13}C NMR of compound **3bf** (75 MHz, $CDCl_3$)

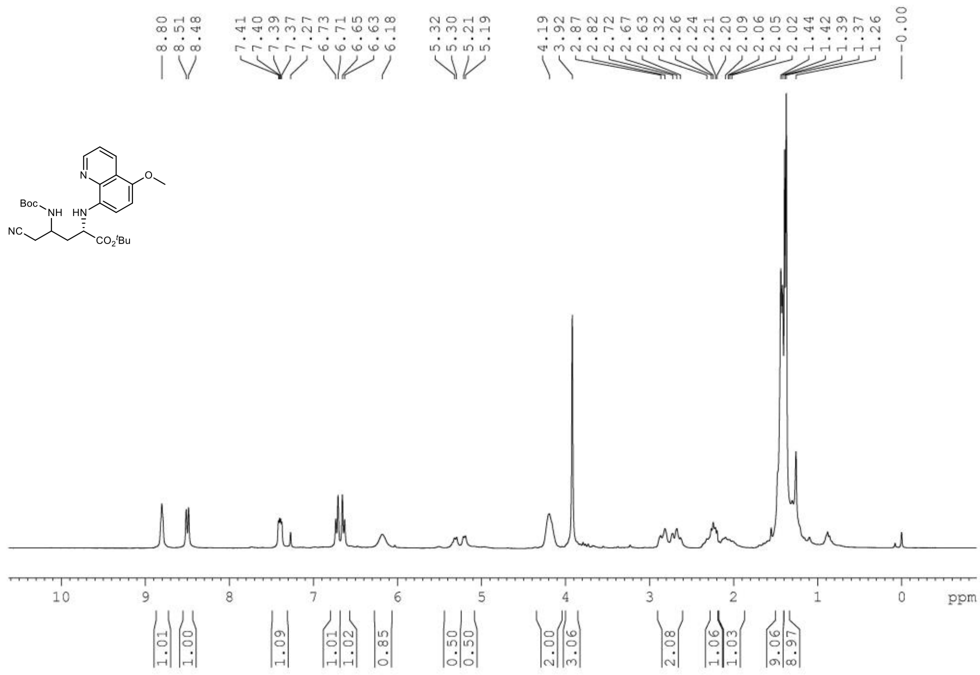

Supplementary Fig. 27. ^{1}H NMR of compound **3bg** (300 MHz, $CDCl_3$)

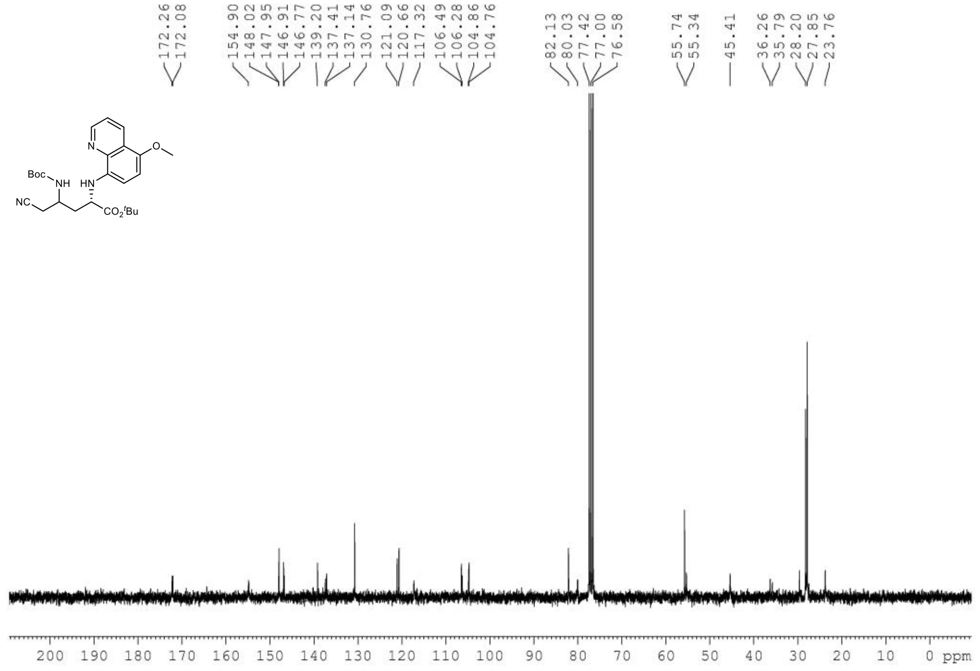

Supplementary Fig. 28. ^{13}C NMR of compound **3bg** (75 MHz, $CDCl_3$)

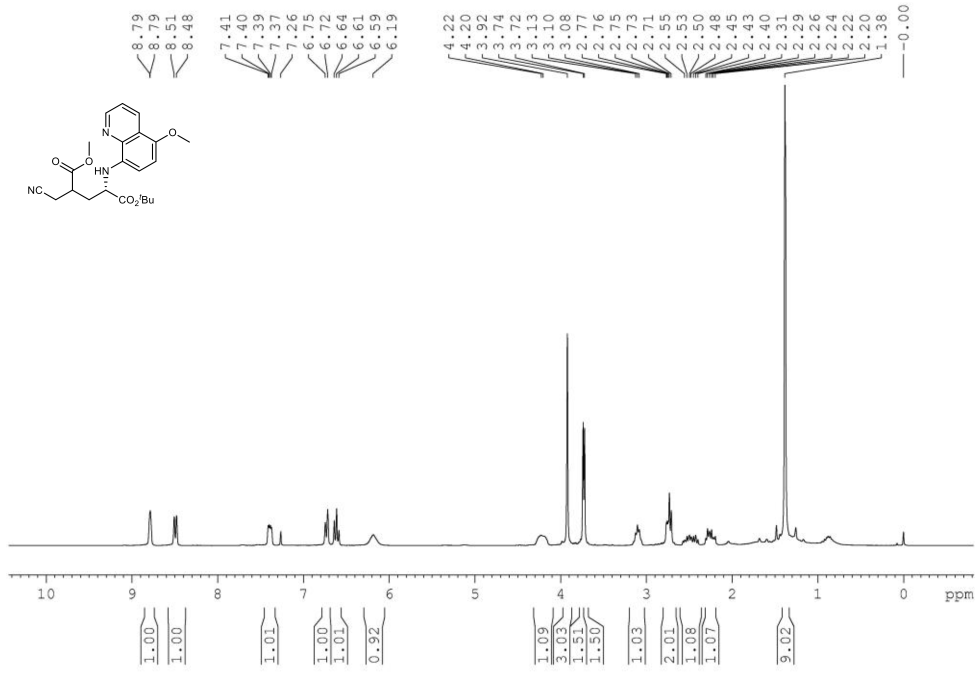

Supplementary Fig. 29. ^{1}H NMR of compound **3bh** (300 MHz, $CDCl_3$)

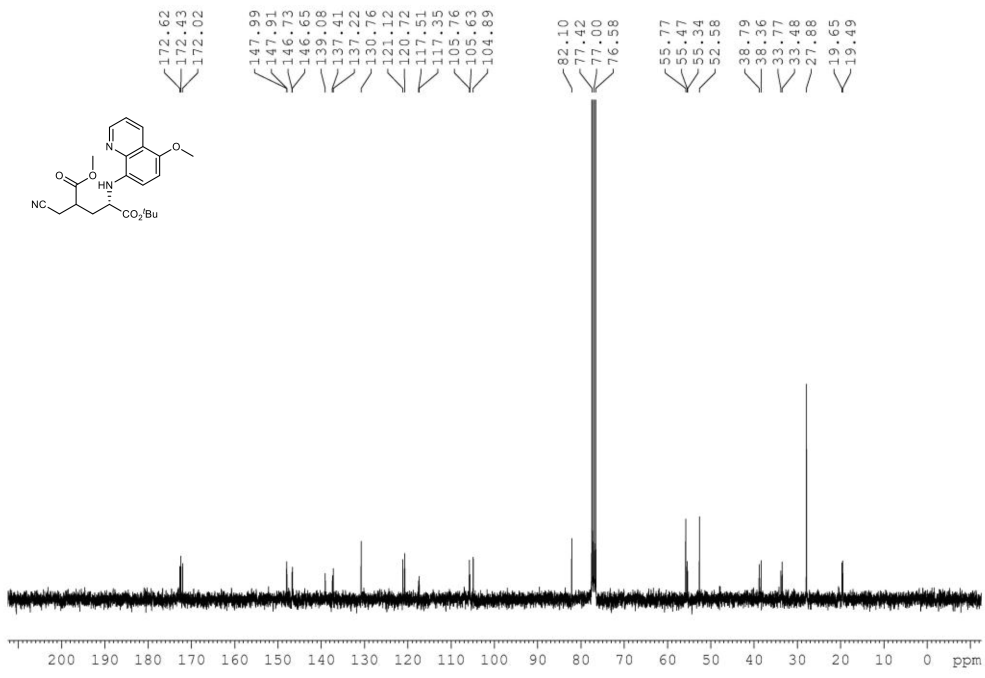

Supplementary Fig. 30. ^{13}C NMR of compound **3bh** (75 MHz, $CDCl_3$)

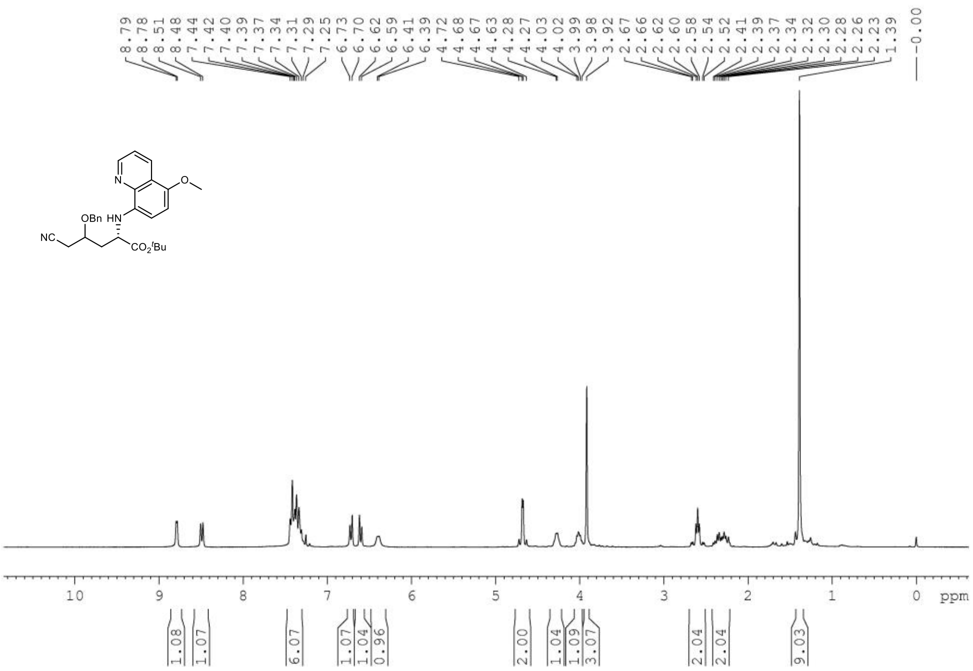

Supplementary Fig. 31. ^{1}H NMR of compound **3bi-1** (300 MHz, $CDCl_3$)

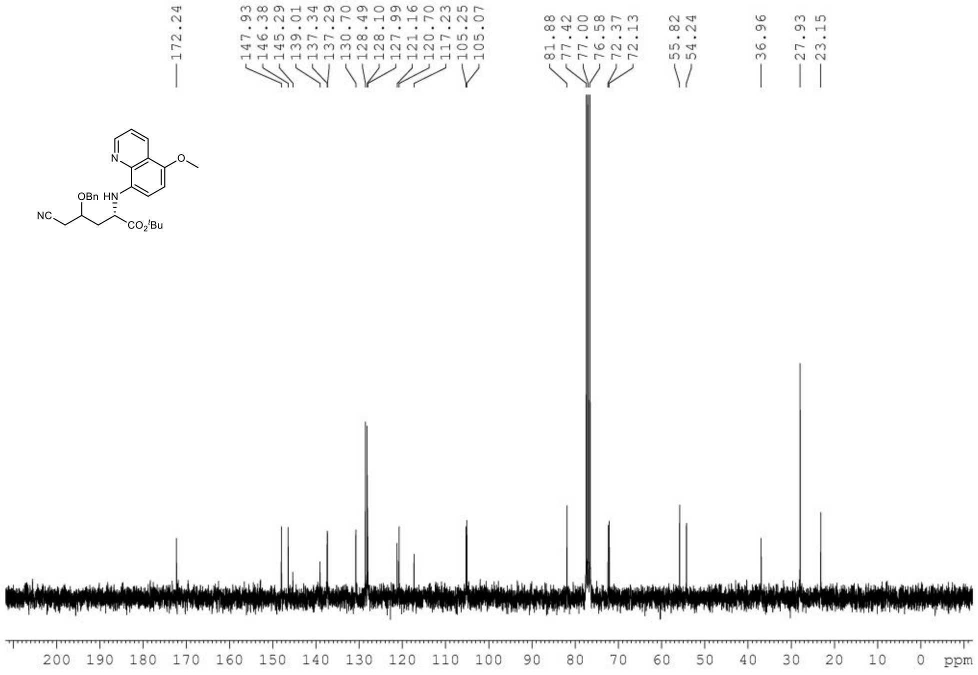

Supplementary Fig. 32. ^{13}C NMR of compound **3bi-1** (75 MHz, $CDCl_3$)

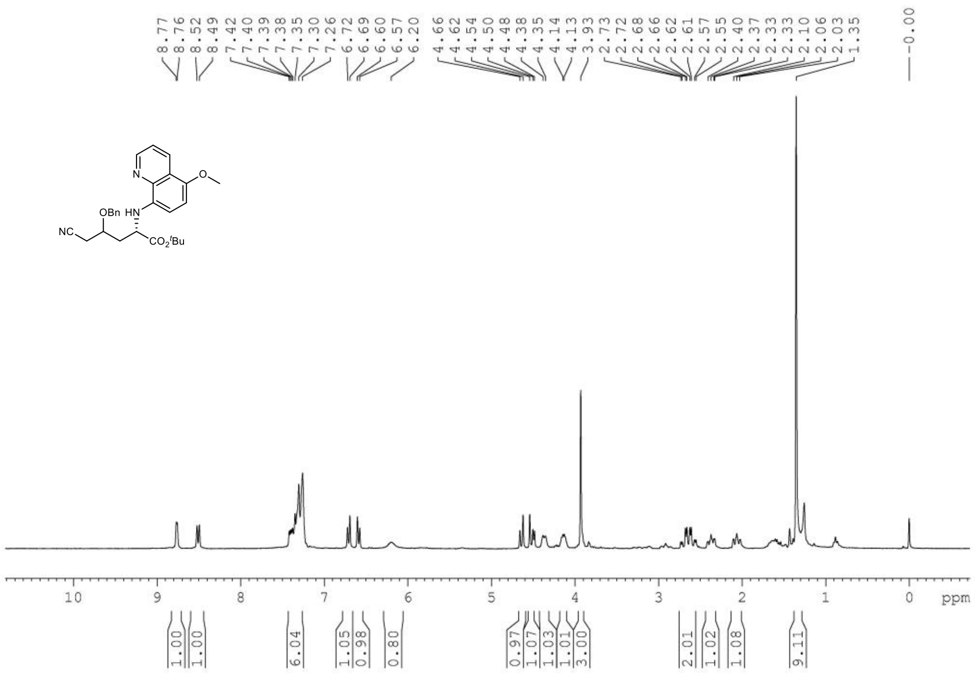

Supplementary Fig. 33. ^{1}H NMR of compound **3bi-2** (300 MHz, $CDCl_3$)

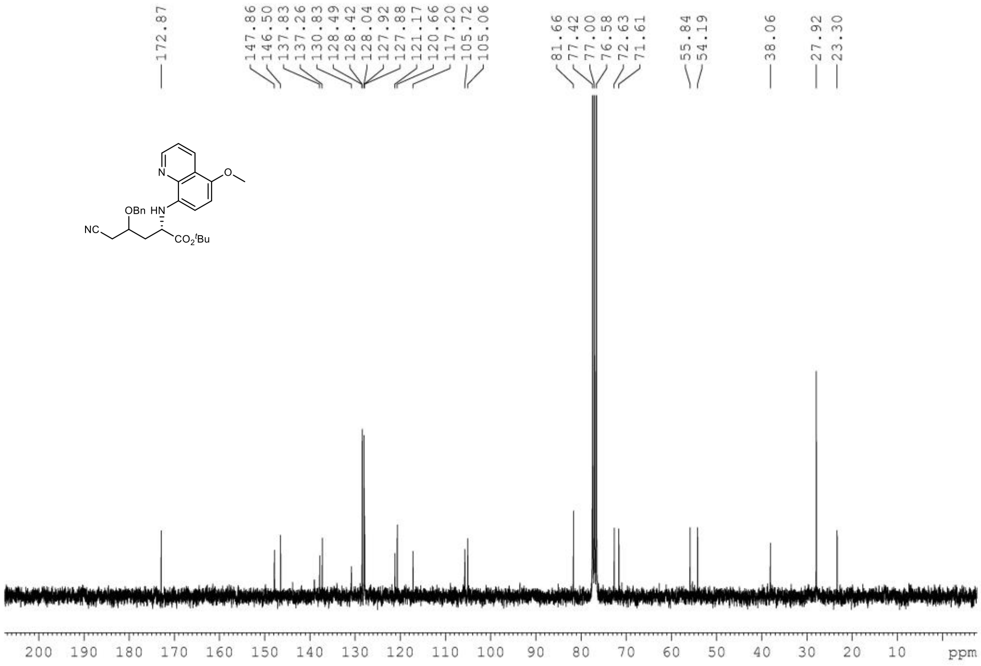

Supplementary Fig. 34. ^{13}C NMR of compound **3bi-2** (75 MHz, $CDCl_3$)

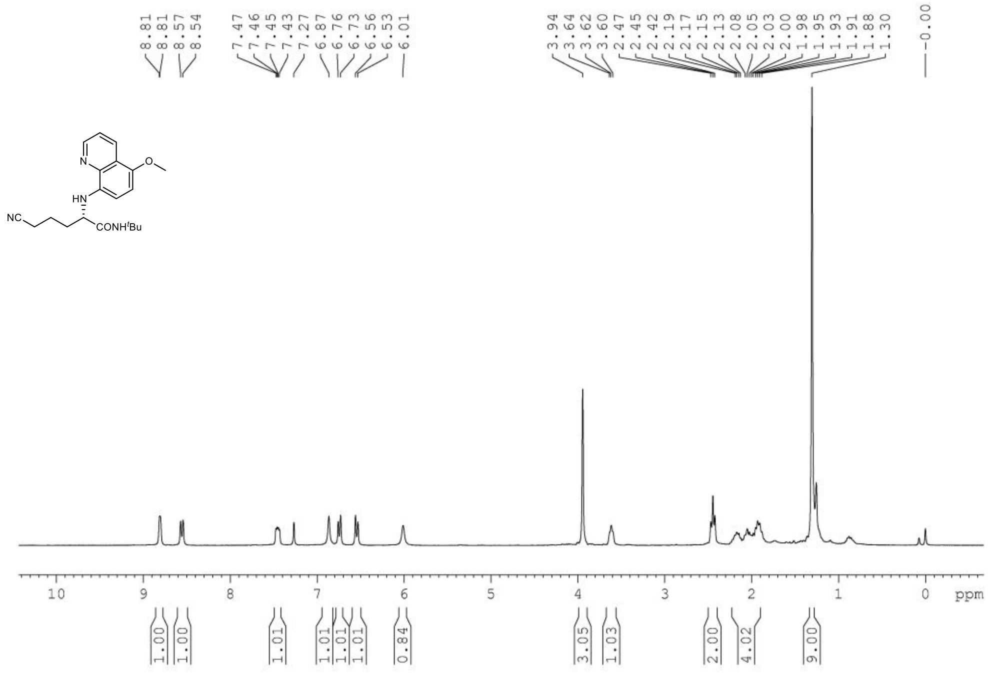

Supplementary Fig. 35. ^{1}H NMR of compound **3ca** (300 MHz, $CDCl_3$)

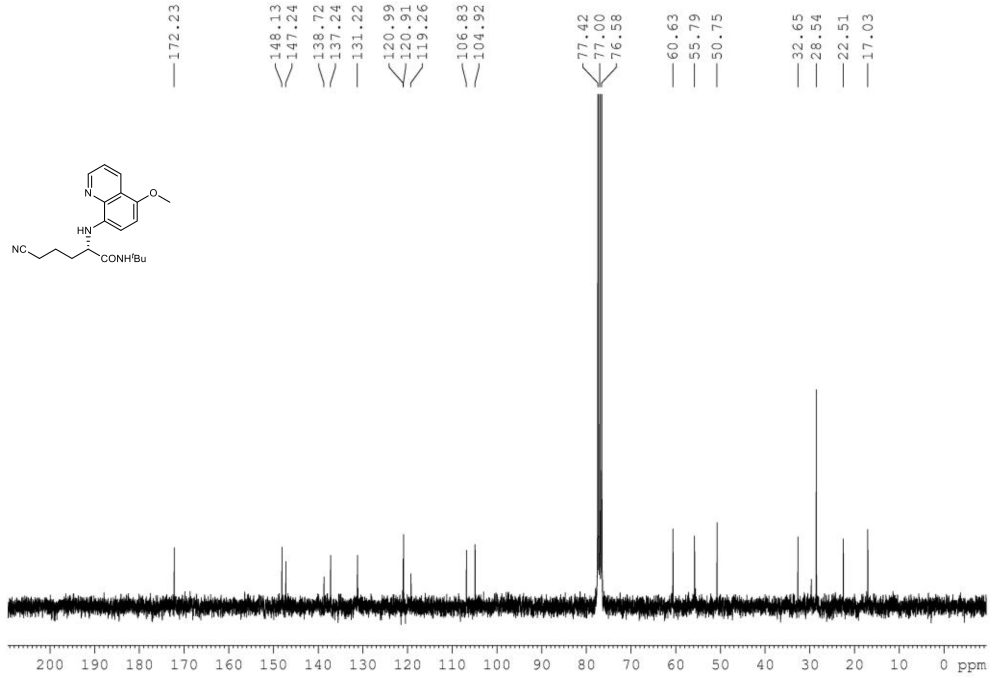

Supplementary Fig. 36. ^{13}C NMR of compound **3ca** (75 MHz, $CDCl_3$)

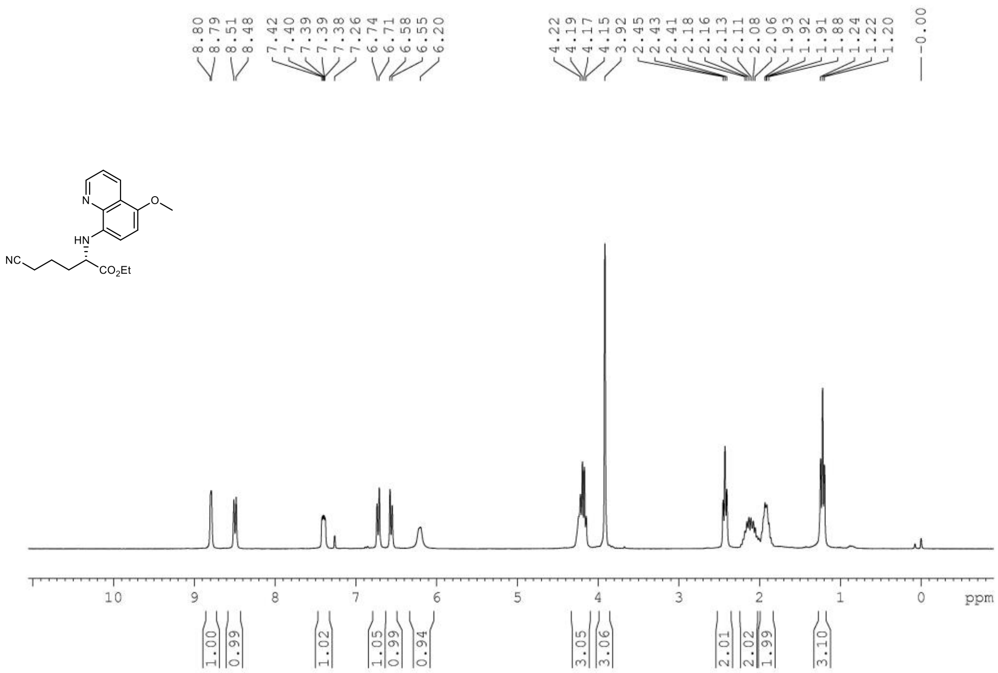

Supplementary Fig. 37. ^{1}H NMR of compound **3da** (300 MHz, $CDCl_3$)

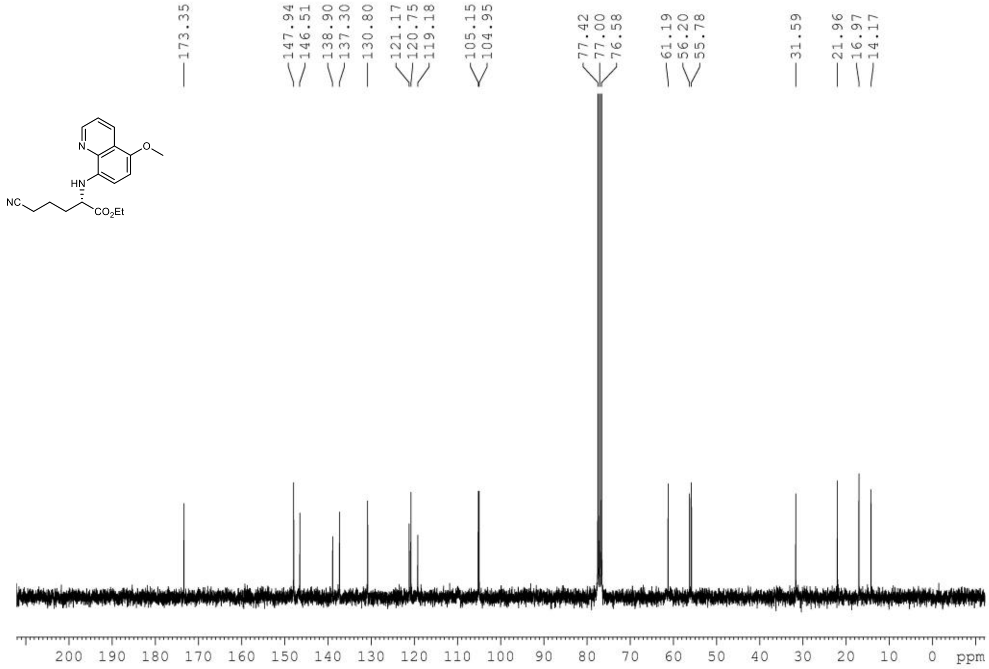

Supplementary Fig. 38. ^{13}C NMR of compound **3da** (75 MHz, $CDCl_3$)

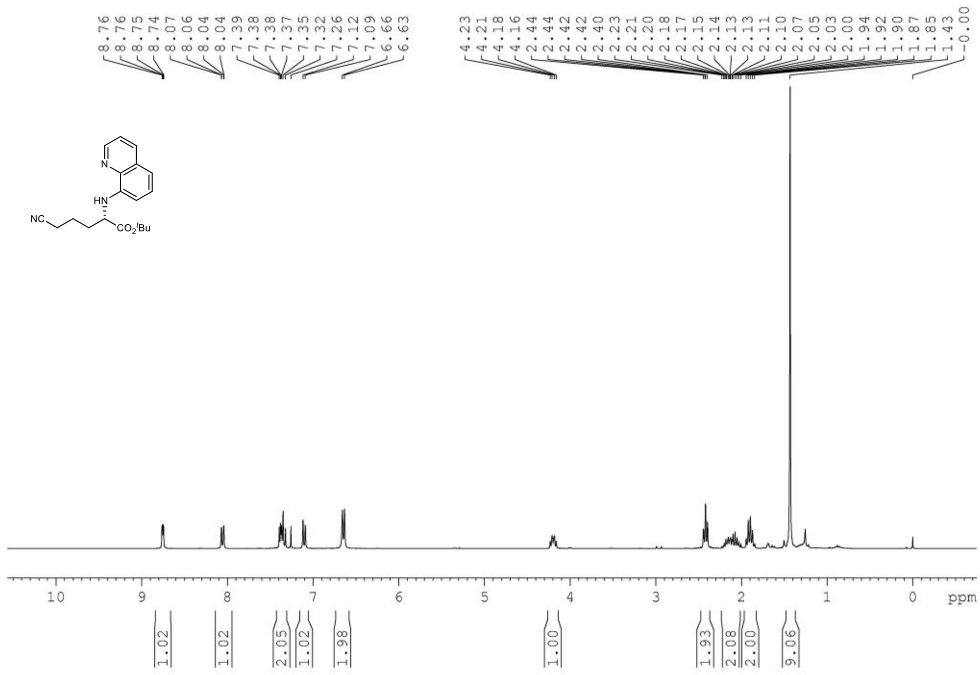

Supplementary Fig. 39. ^{1}H NMR of compound **3ea** (300 MHz, $CDCl_3$)

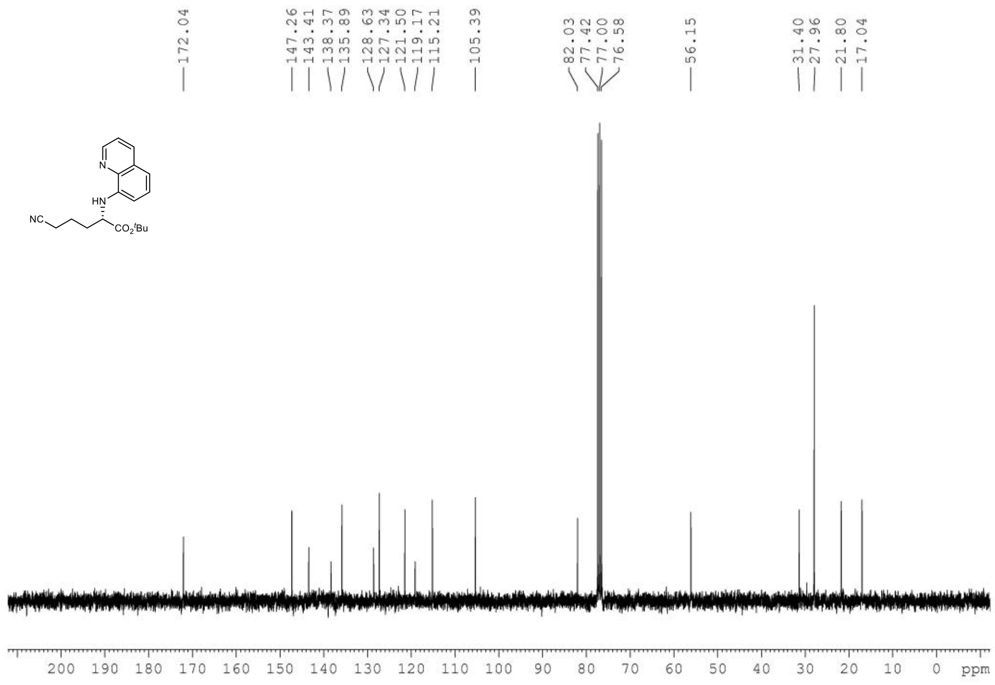

Supplementary Fig. 40. ^{13}C NMR of compound **3ea** (75 MHz, $CDCl_3$)

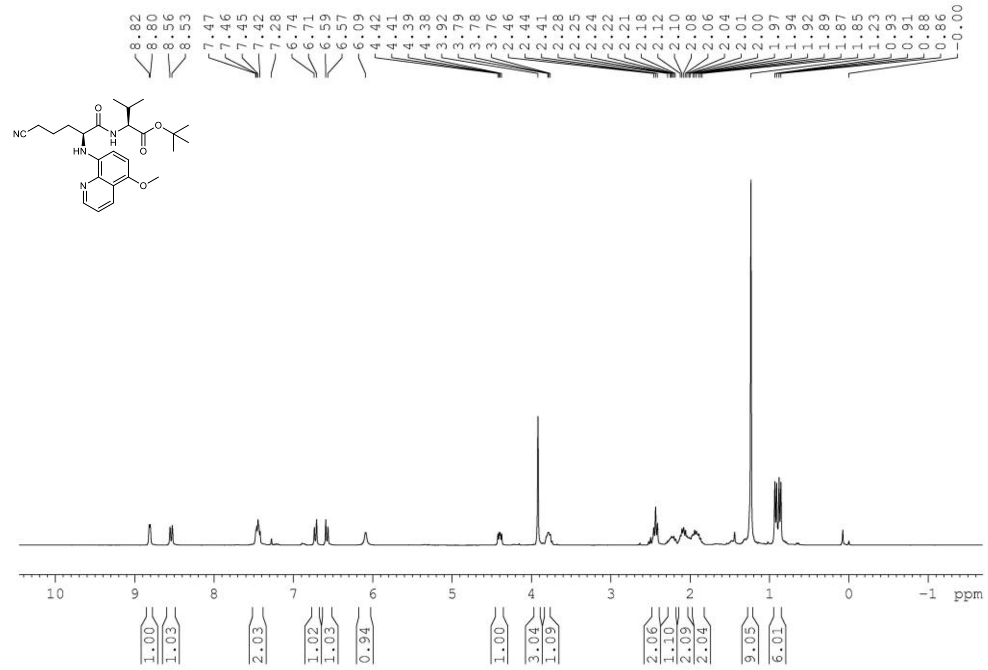

Supplementary Fig. 41. ^{1}H NMR of compound **3fa** (300 MHz, $CDCl_3$)

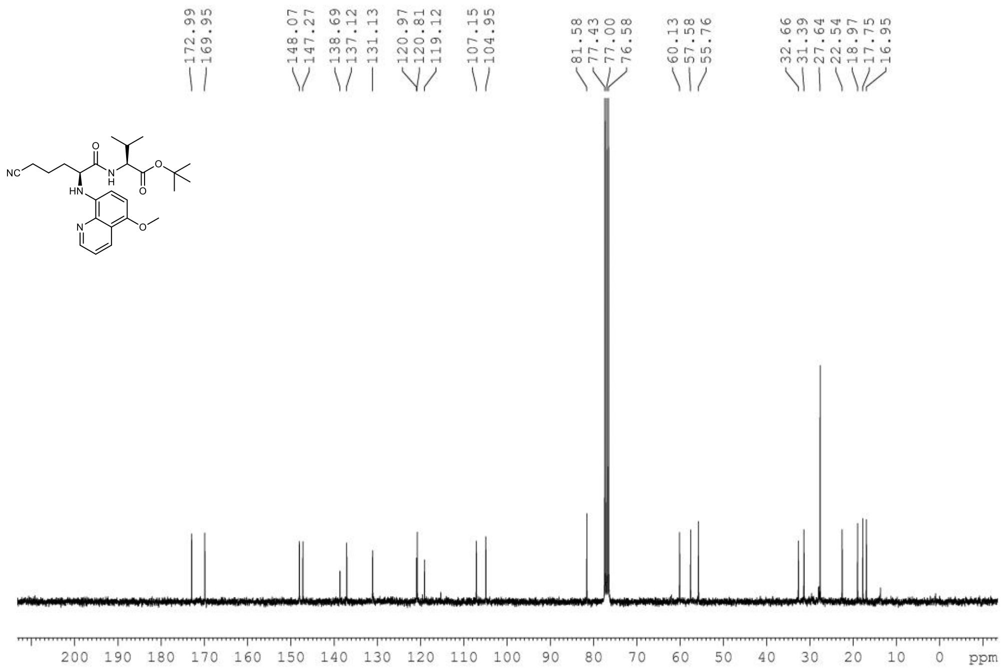

Supplementary Fig. 42. ^{13}C NMR of compound **3fa** (75 MHz, $CDCl_3$)

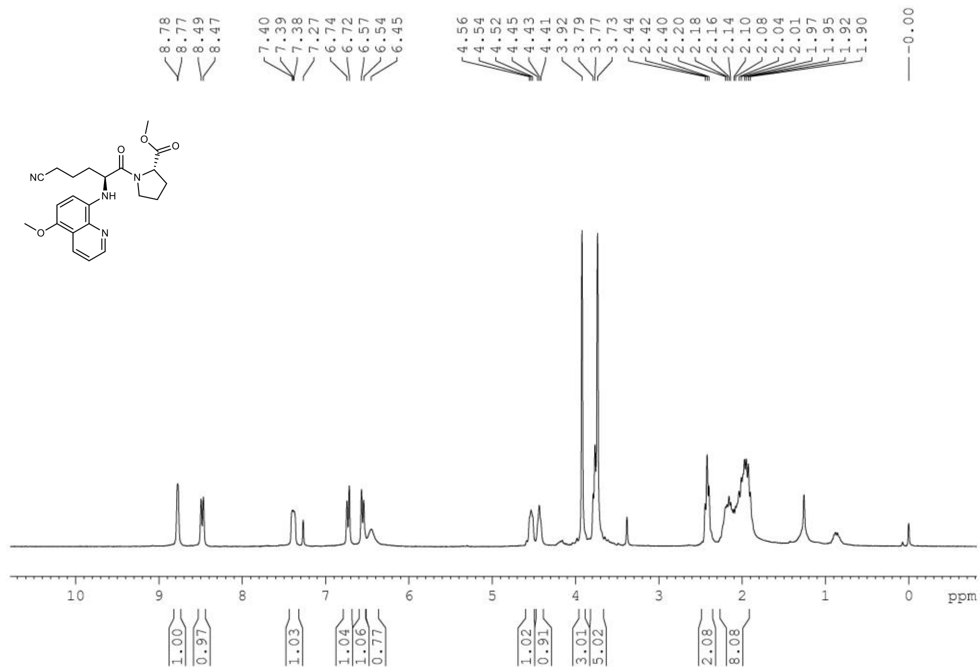

Supplementary Fig. 43. ^{1}H NMR of compound **3ga** (300 MHz, $CDCl_3$)

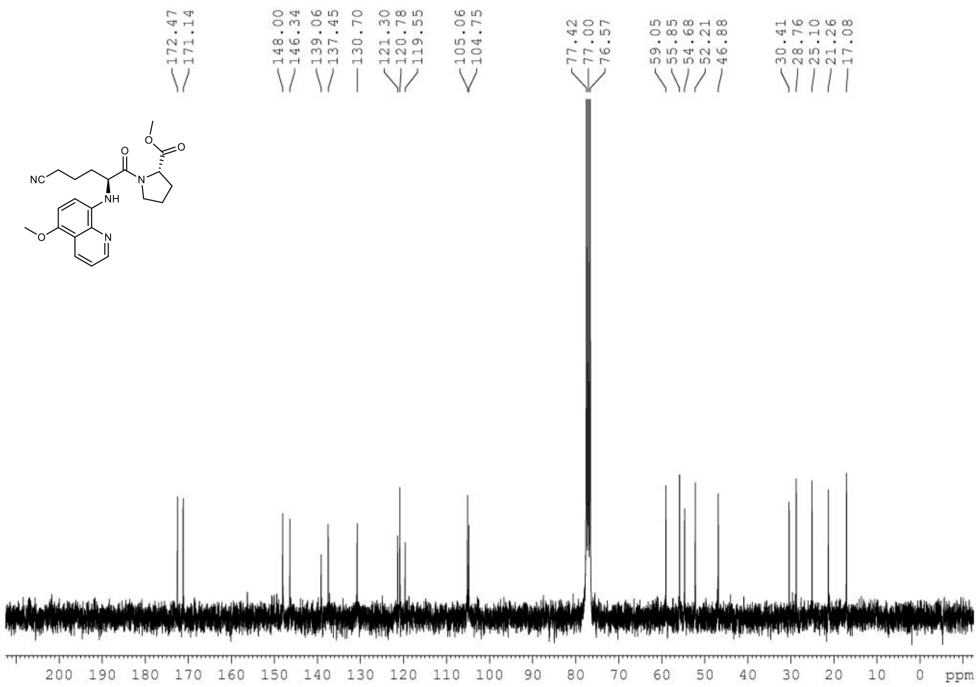

Supplementary Fig. 44. ^{13}C NMR of compound **3ga** (75 MHz, $CDCl_3$)

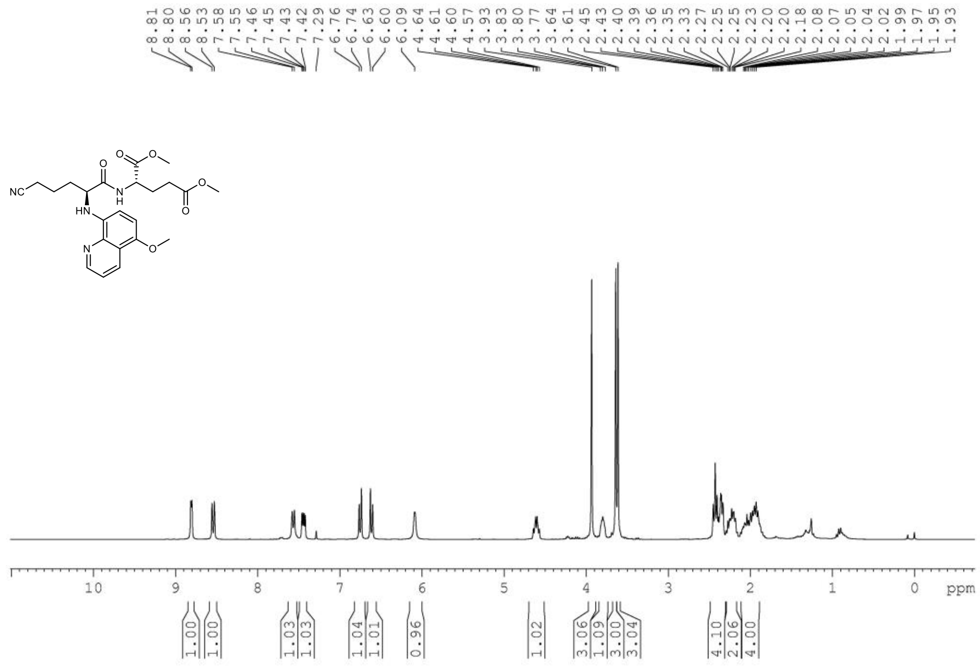

Supplementary Fig. 45. ^{1}H NMR of compound **3ha** (300 MHz, $CDCl_3$)

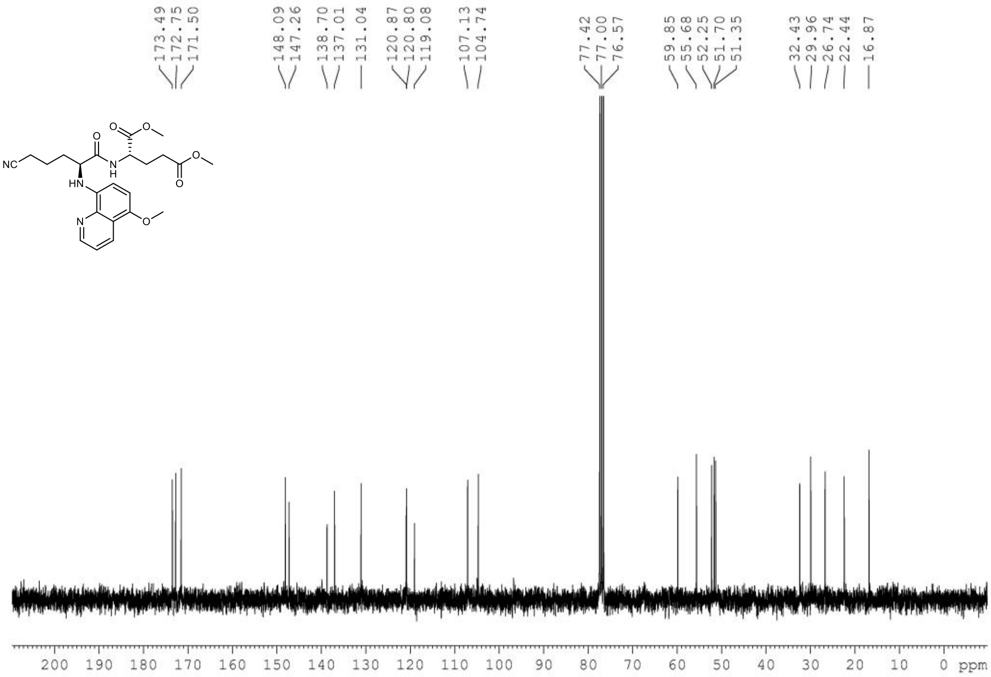

Supplementary Fig. 46. ^{13}C NMR of compound **3ha** (75 MHz, $CDCl_3$)

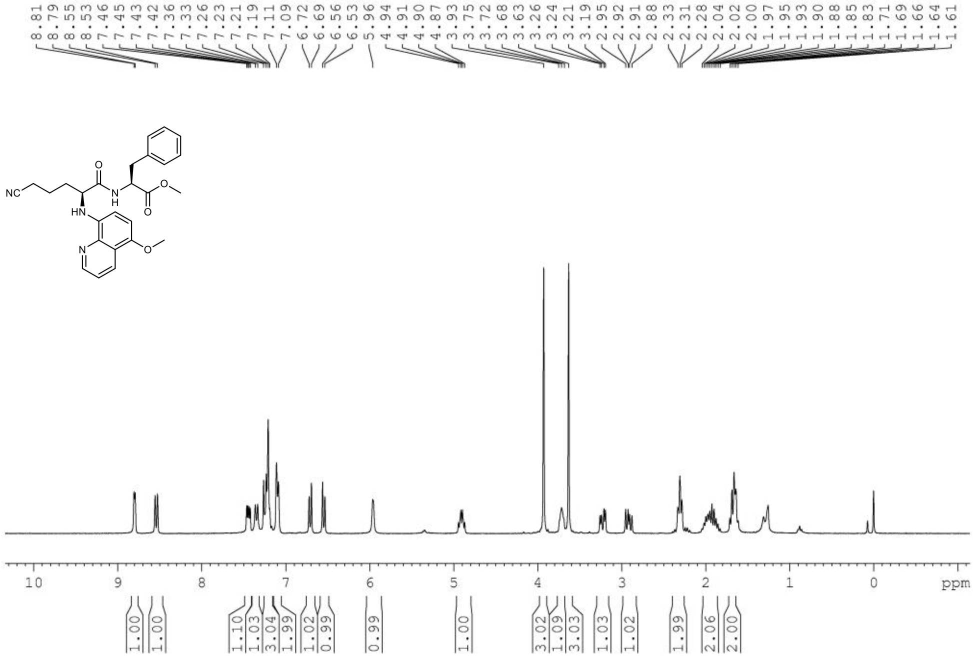

Supplementary Fig. 47. ^{1}H NMR of compound **3ia** (300 MHz, $CDCl_3$)

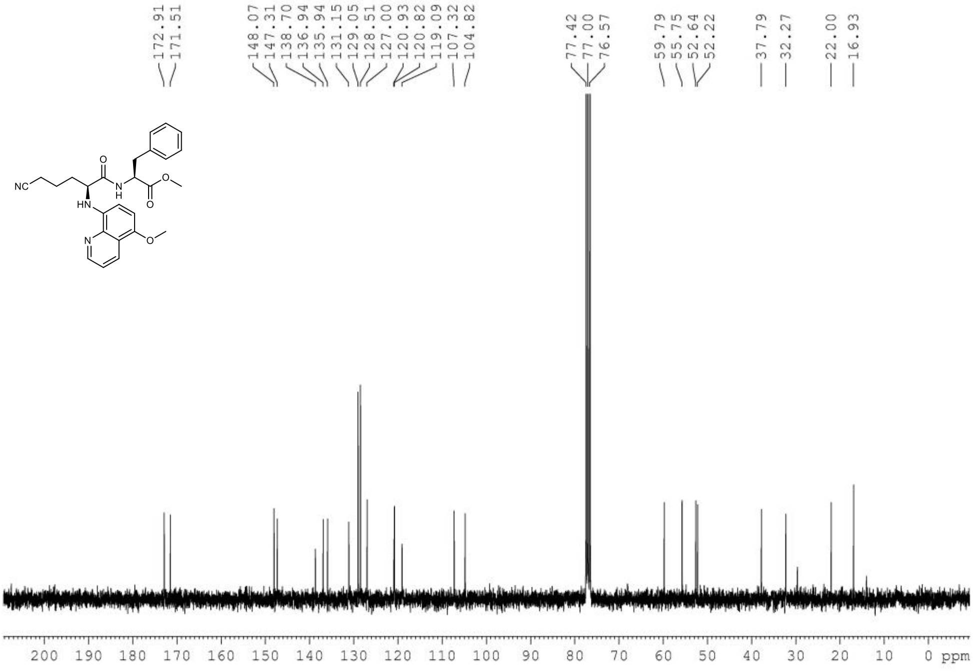

Supplementary Fig. 48. ^{13}C NMR of compound **3ia** (75 MHz, $CDCl_3$)

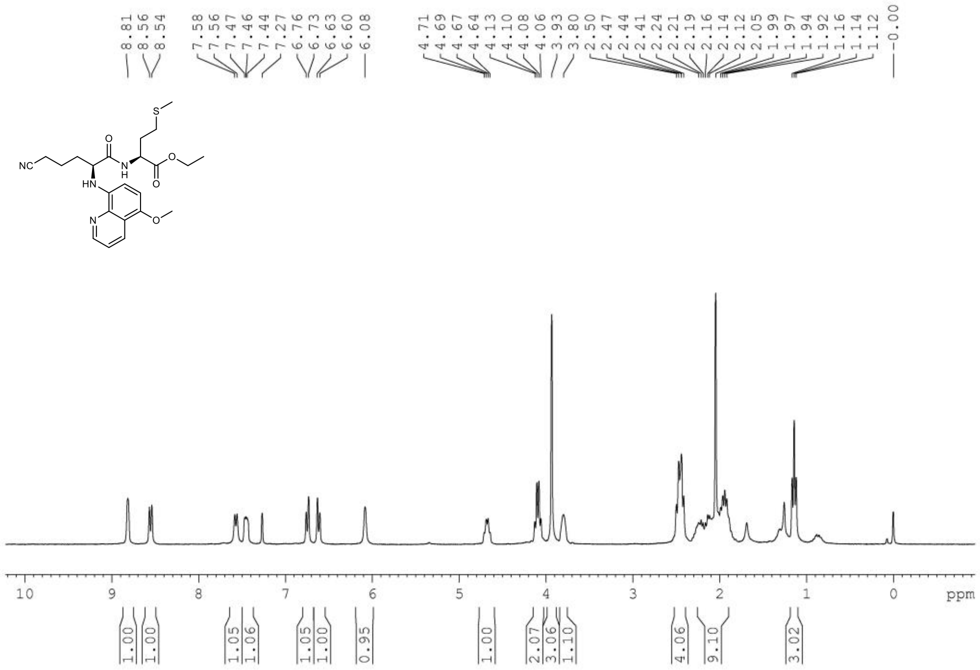

Supplementary Fig. 49. ^{1}H NMR of compound **3ja** (300 MHz, $CDCl_3$)

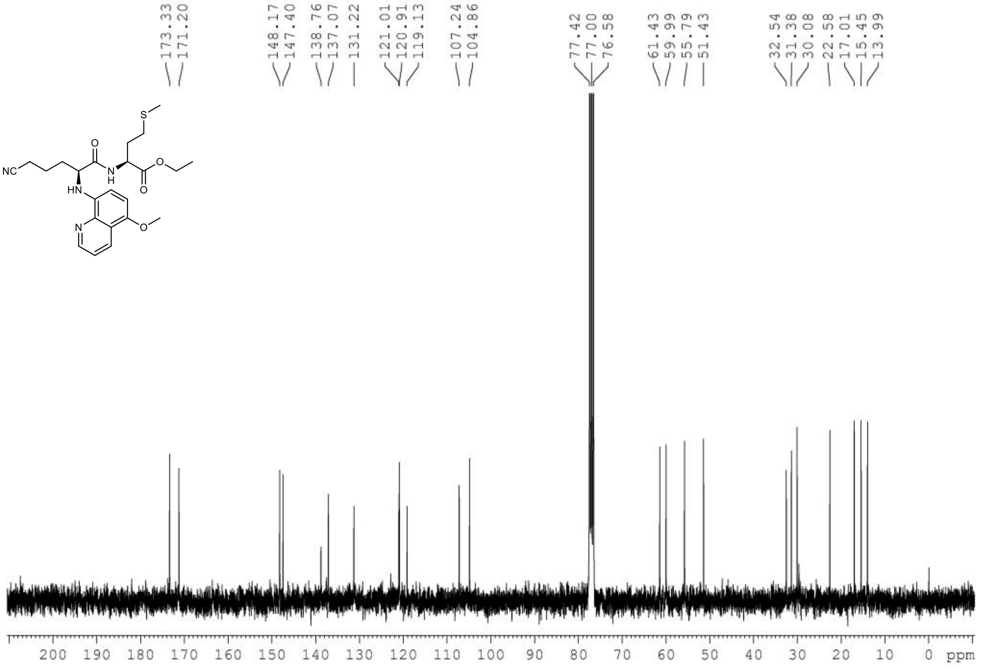

Supplementary Fig. 50. ^{13}C NMR of compound **3ja** (75 MHz, $CDCl_3$)

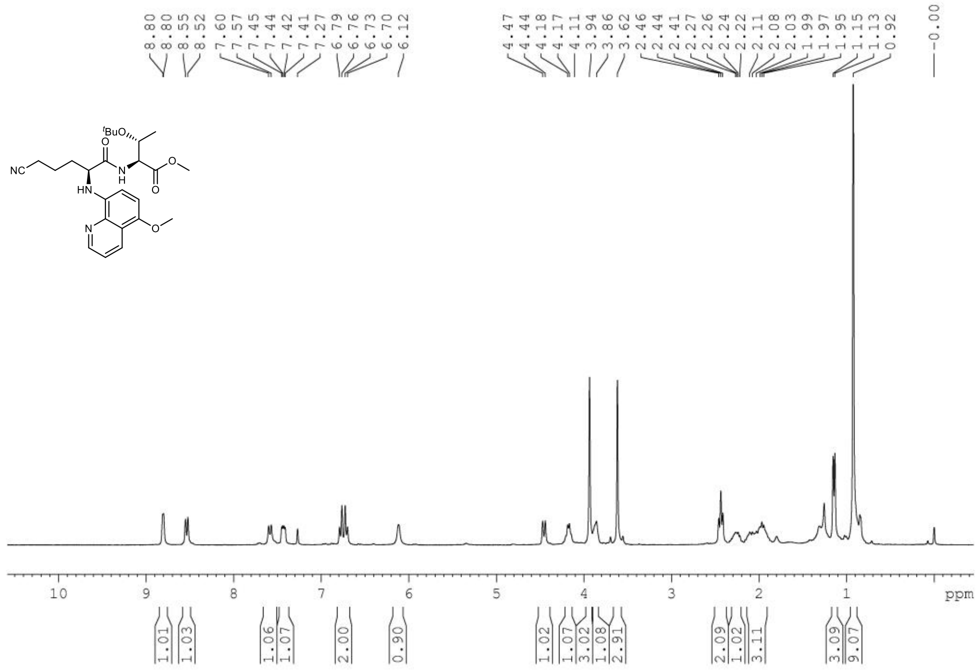

Supplementary Fig. 51. ^{1}H NMR of compound **3ka** (300 MHz, $CDCl_3$)

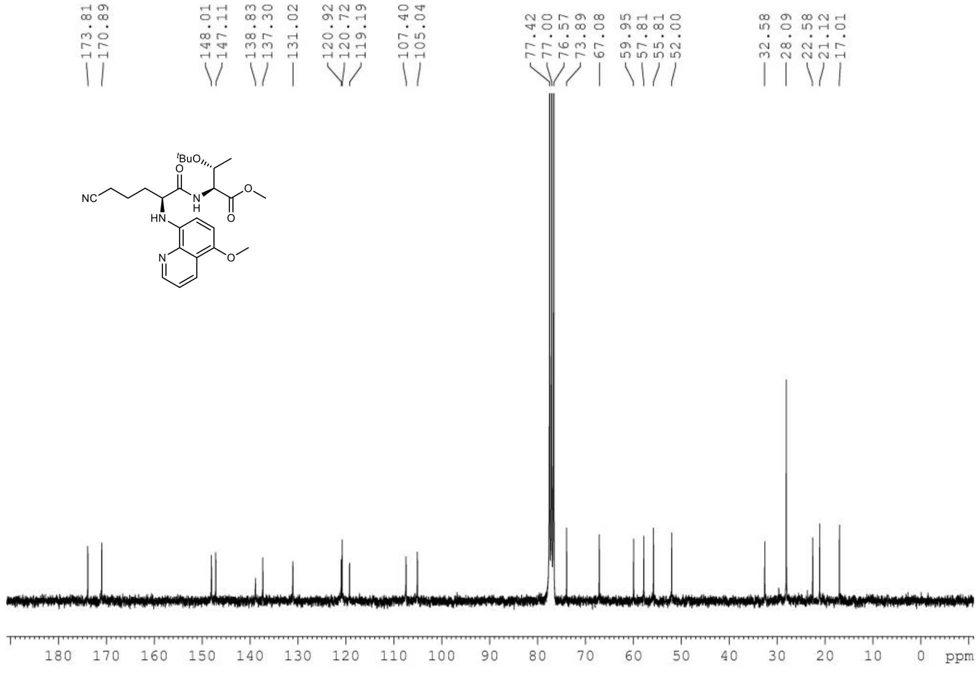

Supplementary Fig. 52. ^{13}C NMR of compound **3ka** (75 MHz, $CDCl_3$)

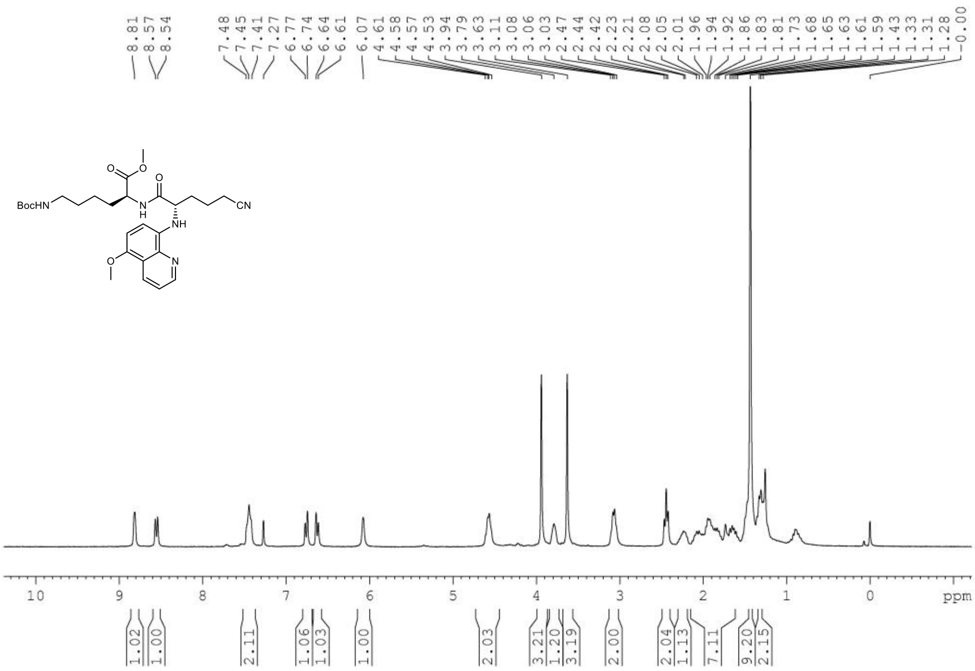

Supplementary Fig. 53. ^{1}H NMR of compound **3la** (300 MHz, $CDCl_3$)

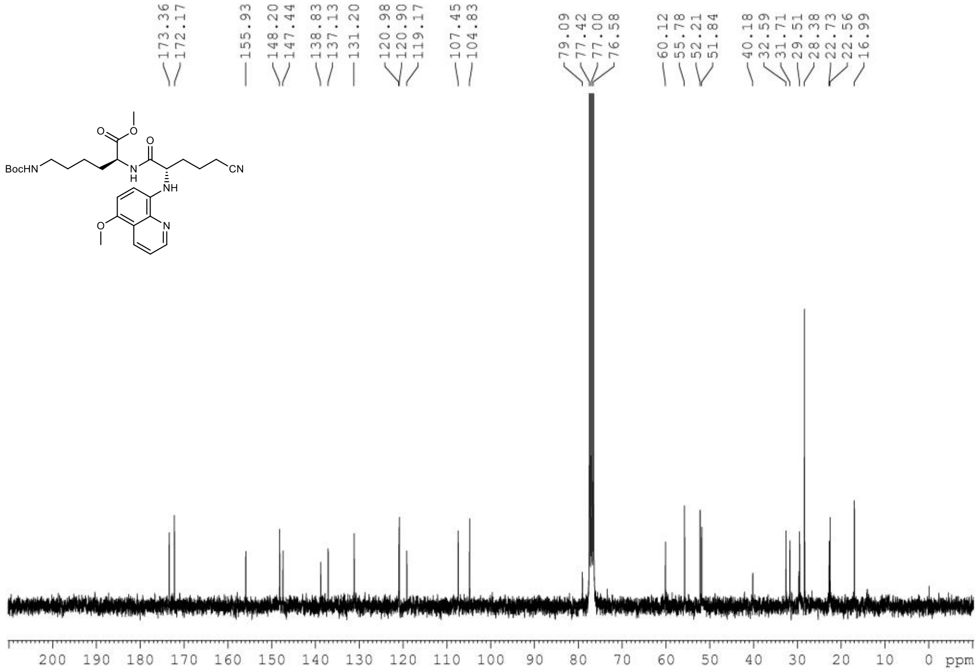

Supplementary Fig. 54. ^{13}C NMR of compound **3la** (75 MHz, $CDCl_3$)

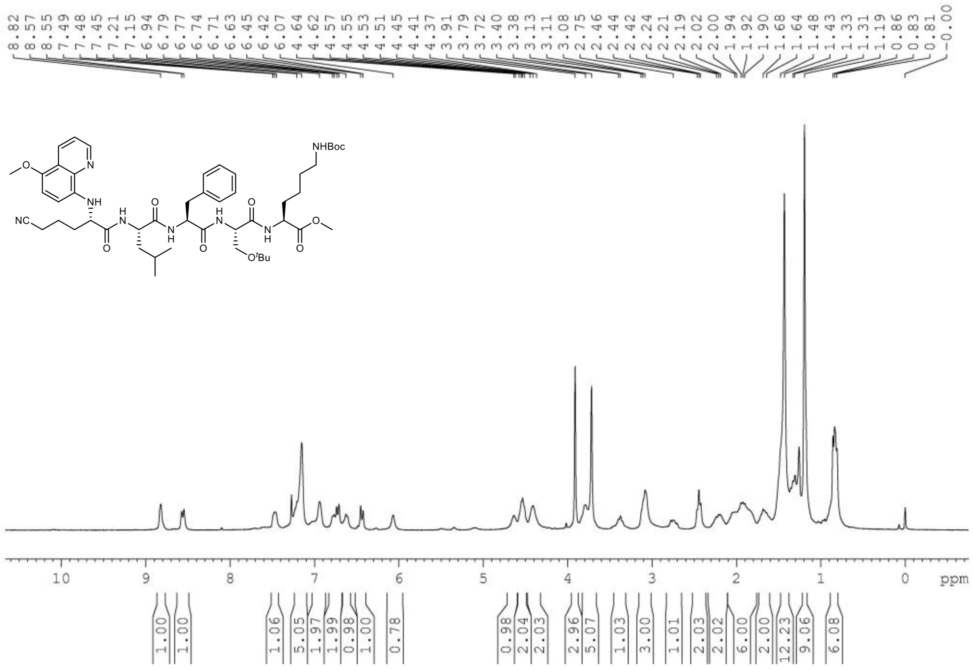

Supplementary Fig. 55. ^{1}H NMR of compound **3ma** (300 MHz, $CDCl_3$)

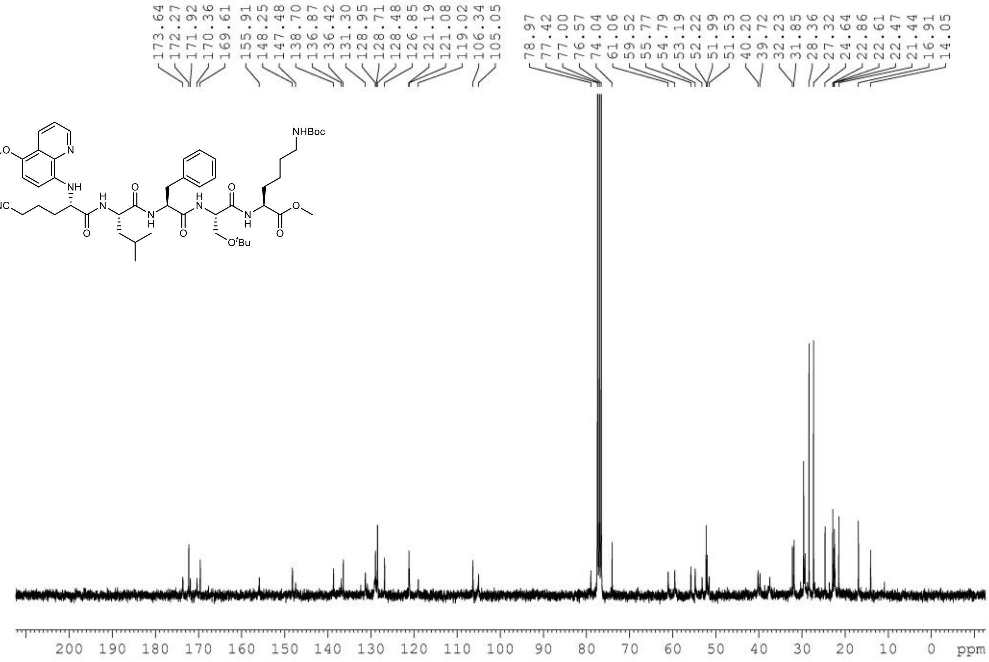

Supplementary Fig. 56. ^{13}C NMR of compound **3ma** (75 MHz, $CDCl_3$)

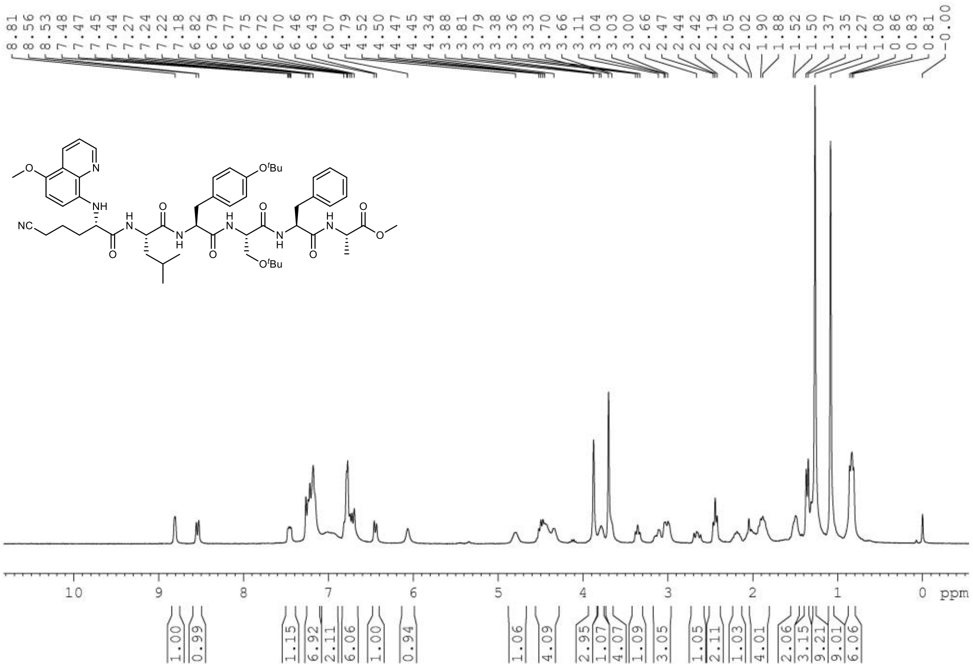

Supplementary Fig. 57. ^{1}H NMR of compound **3na** (300 MHz, $CDCl_3$)

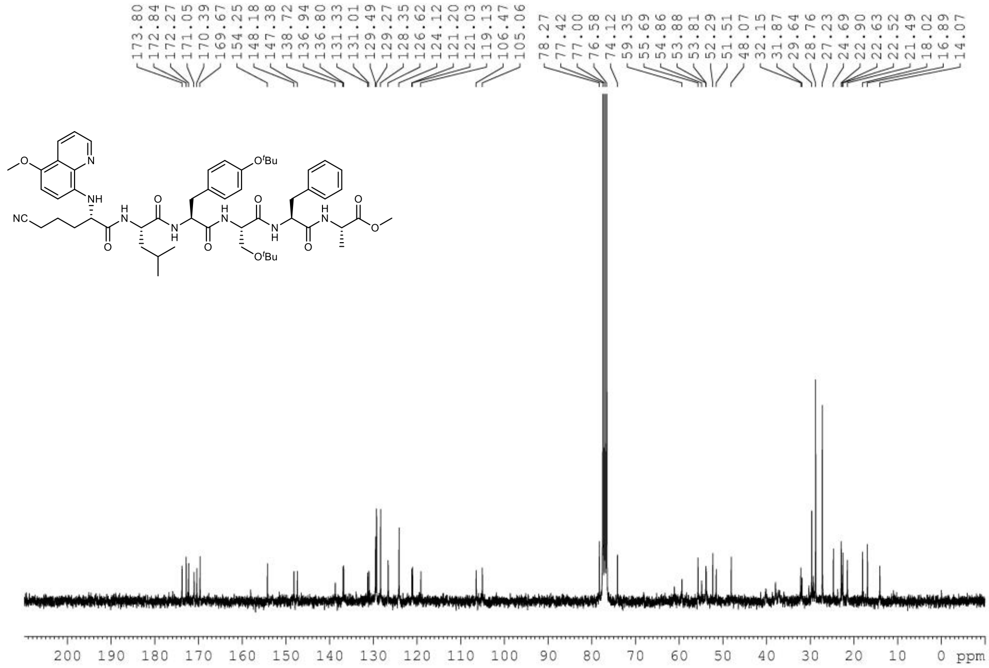

Supplementary Fig. 58. ^{13}C NMR of compound **3na** (75 MHz, $CDCl_3$)

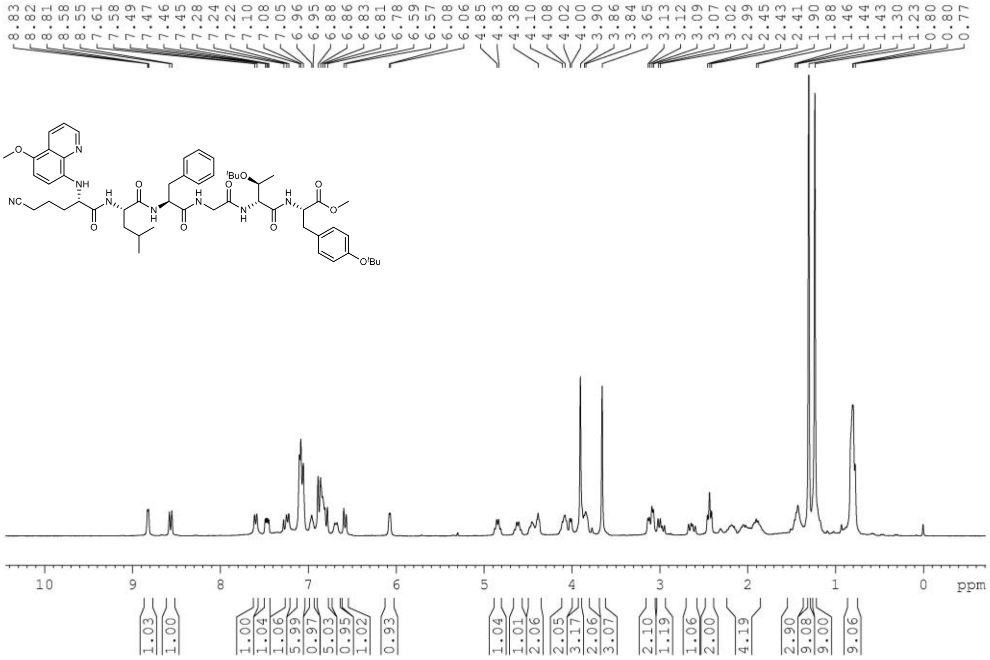

Supplementary Fig. 59. ^{1}H NMR of compound **3oa** (300 MHz, $CDCl_3$)

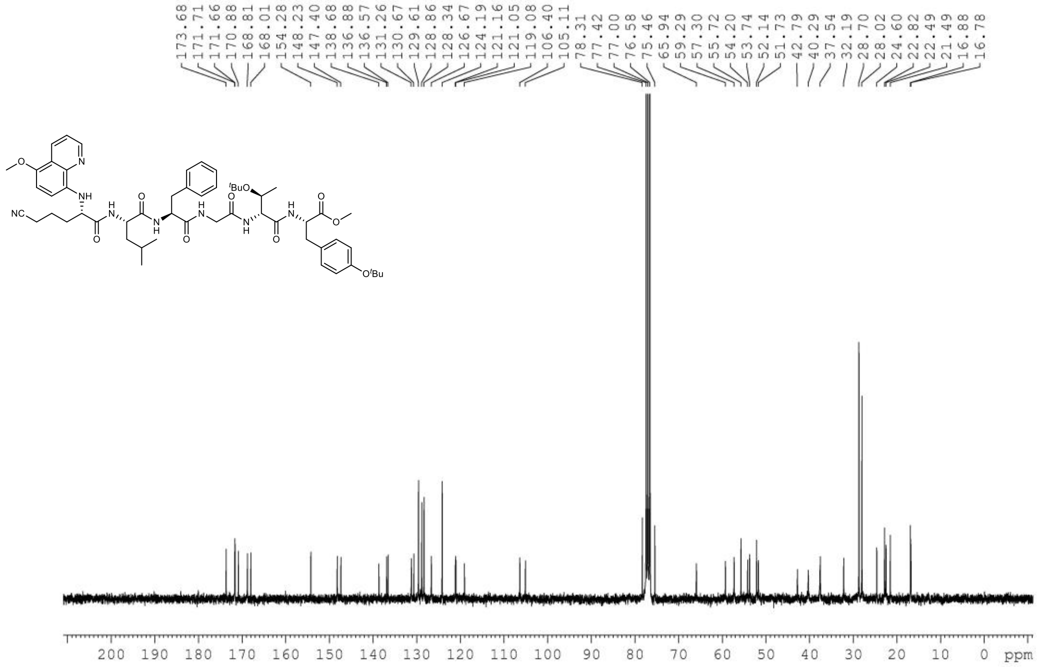

Supplementary Fig. 60. ^{13}C NMR of compound **3oa** (75 MHz, $CDCl_3$)

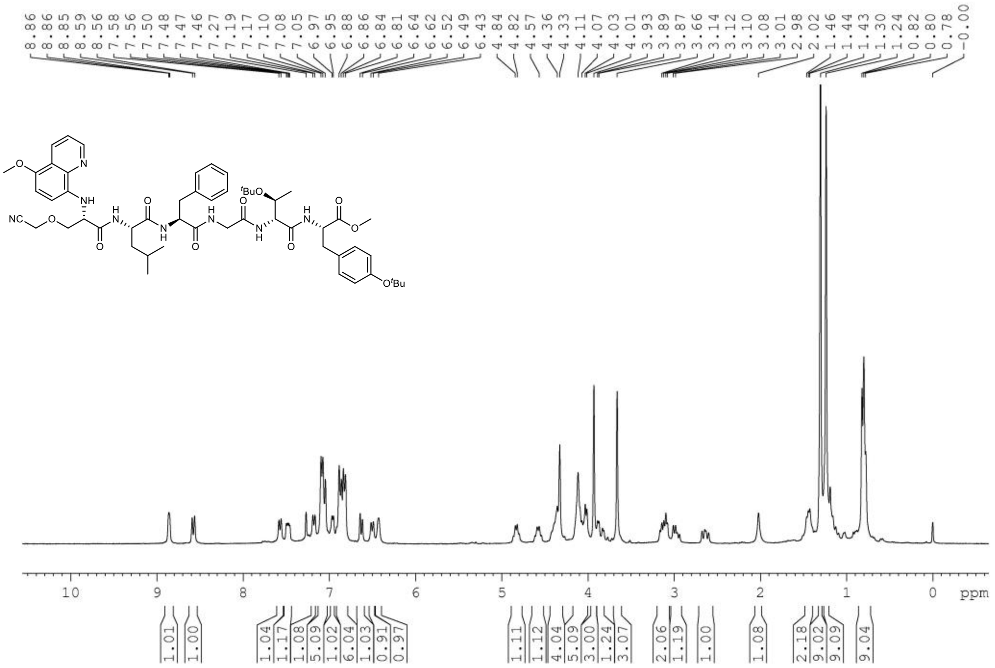

Supplementary Fig. 61. ^{1}H NMR of compound **3ob** (300 MHz, $CDCl_3$)

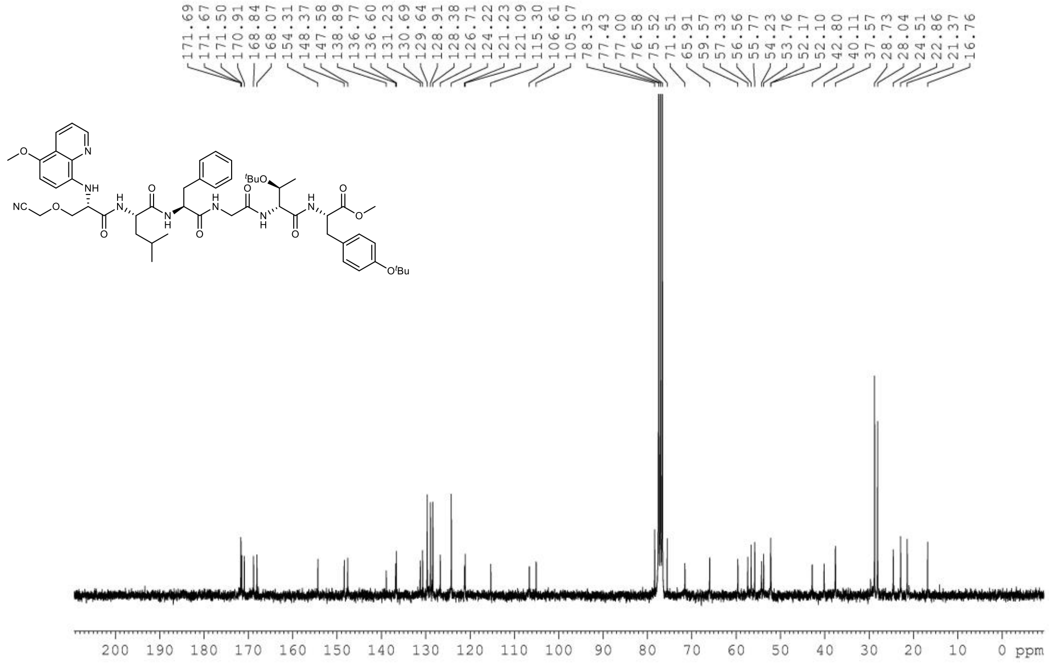

Supplementary Fig. 62. ^{13}C NMR of compound **3ob** (75 MHz, $CDCl_3$)

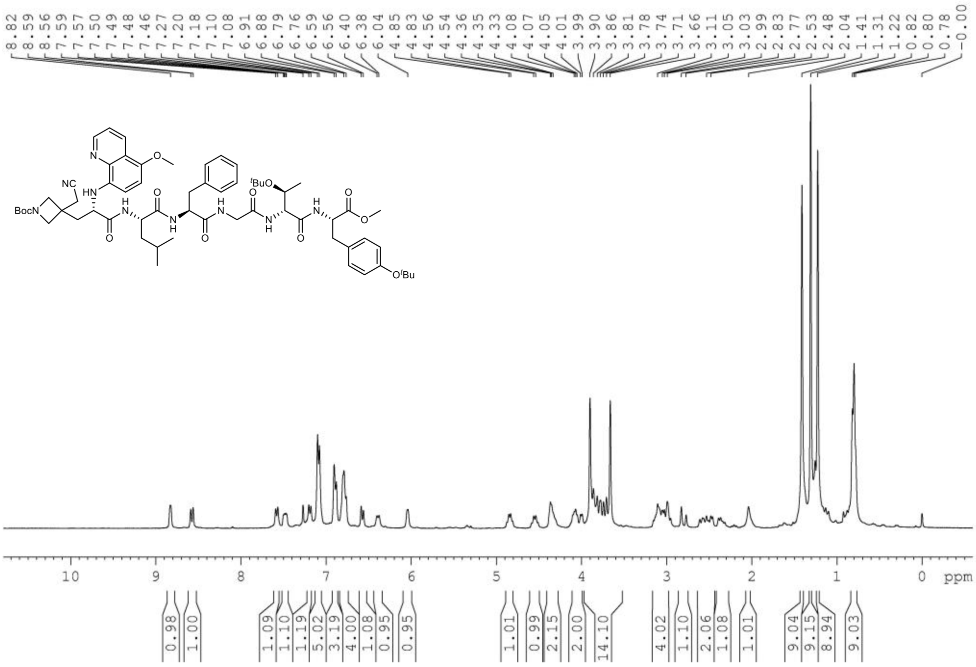

Supplementary Fig. 63. ^{1}H NMR of compound **3oe** (300 MHz, $CDCl_3$)

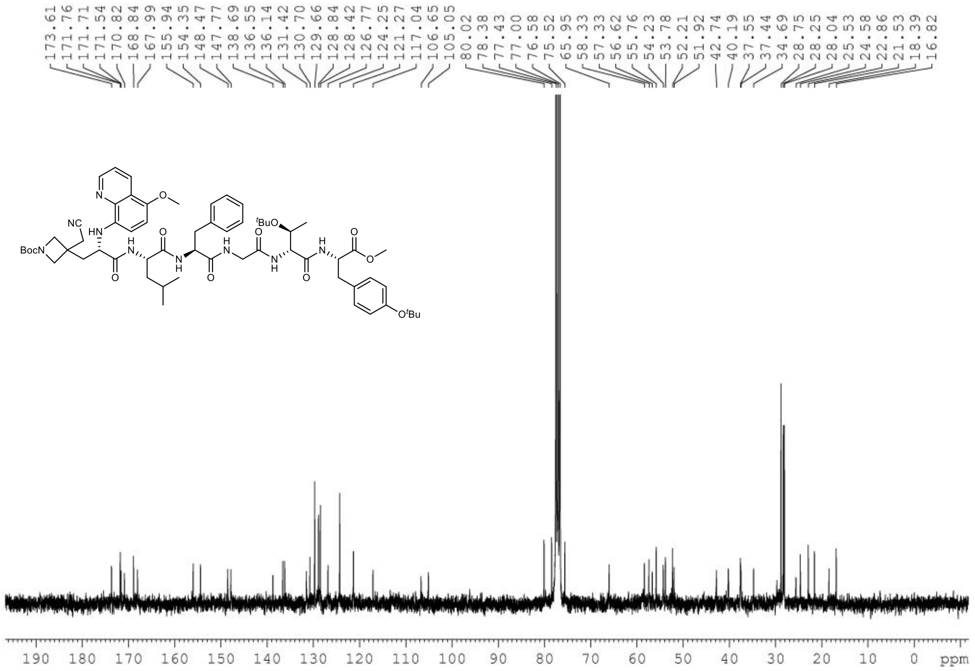

Supplementary Fig. 64. ^{13}C NMR of compound **3oe** (75 MHz, $CDCl_3$)

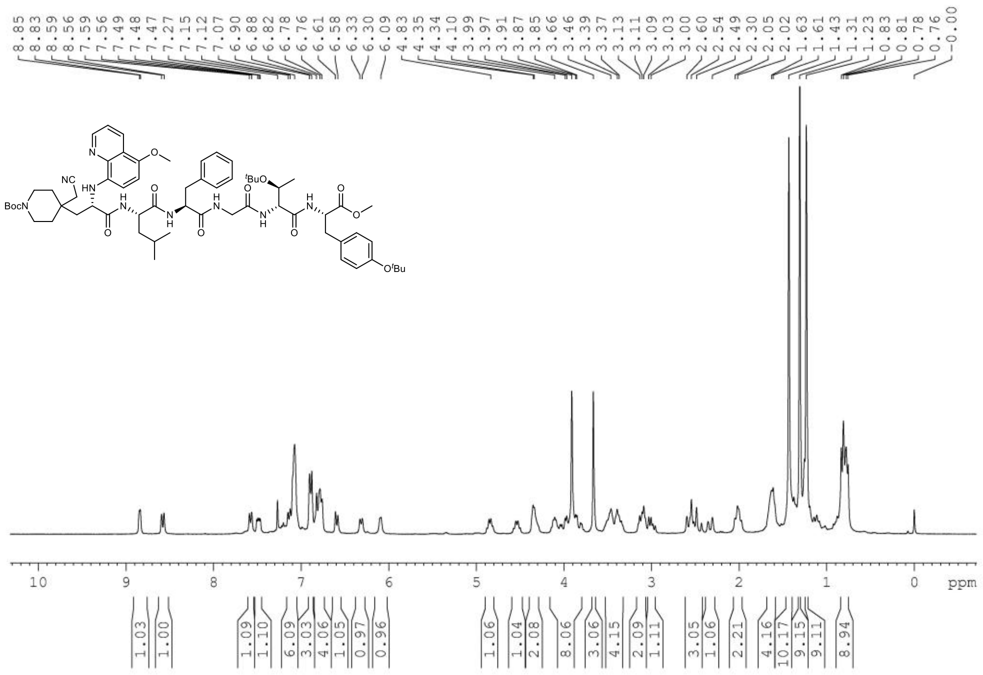

Supplementary Fig. 65. ^{1}H NMR of compound **3of** (300 MHz, $CDCl_3$)

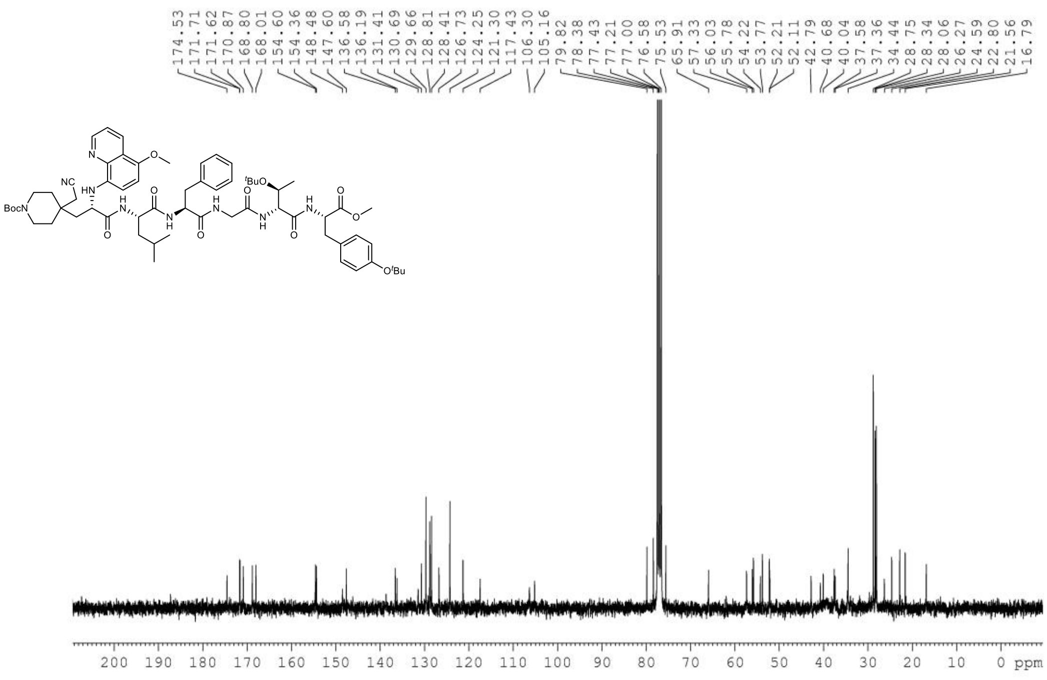

Supplementary Fig. 66. ^{13}C NMR of compound **3of** (75 MHz, $CDCl_3$)

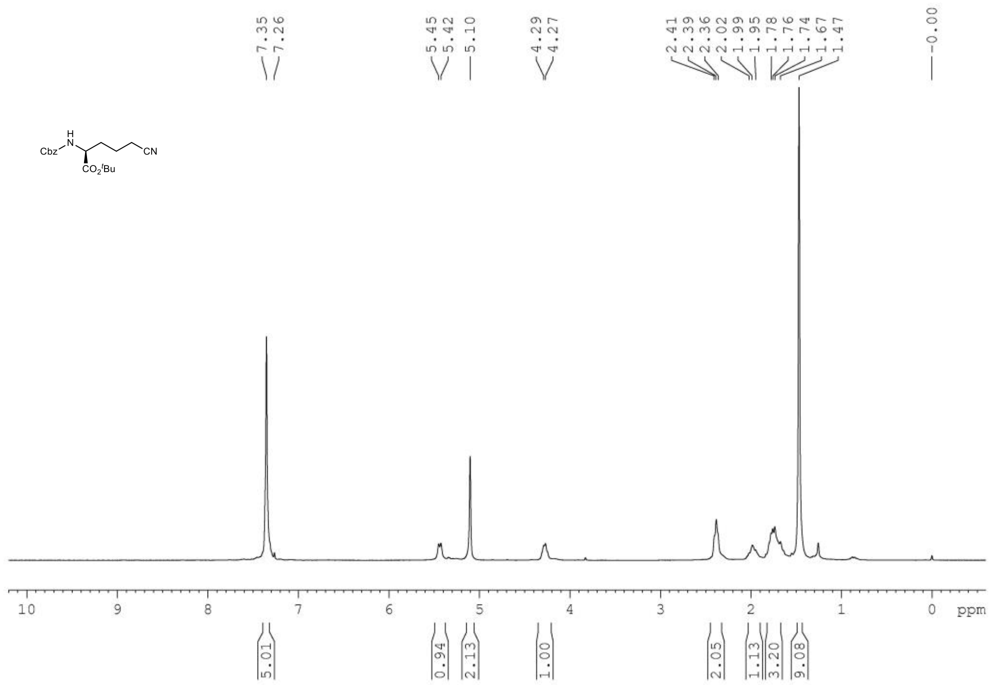

Supplementary Fig. 67. ^{1}H NMR of compound **4** (300 MHz, $CDCl_3$)

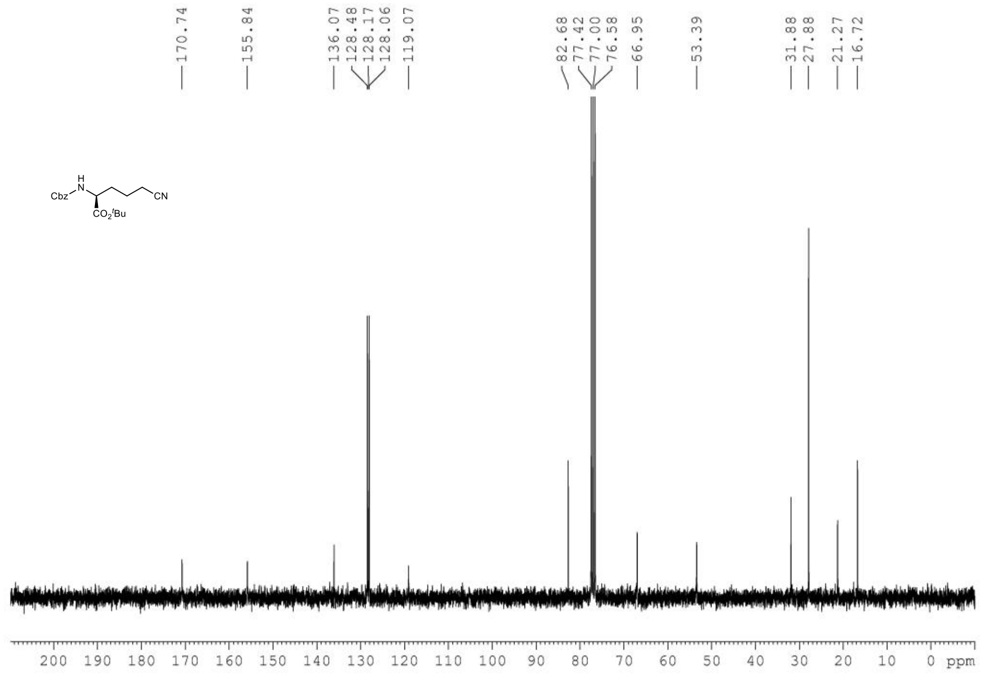

Supplementary Fig. 68. ^{13}C NMR of compound **4** (75 MHz, $CDCl_3$)

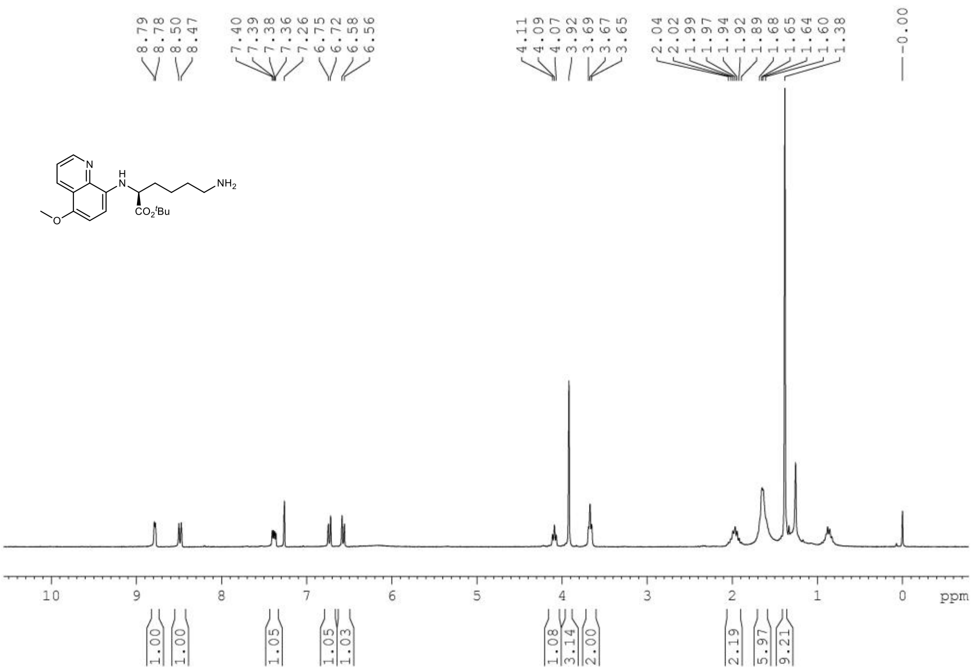

Supplementary Fig. 69. ^{1}H NMR of compound **5** (300 MHz, $CDCl_3$)

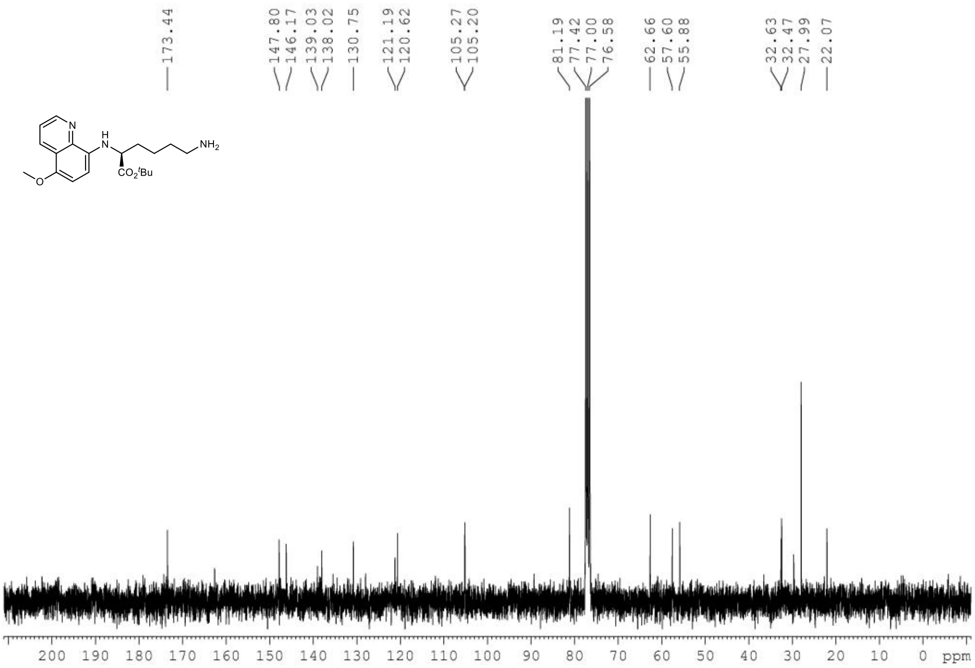

Supplementary Fig. 70. ^{13}C NMR of compound **5** (75 MHz, $CDCl_3$)

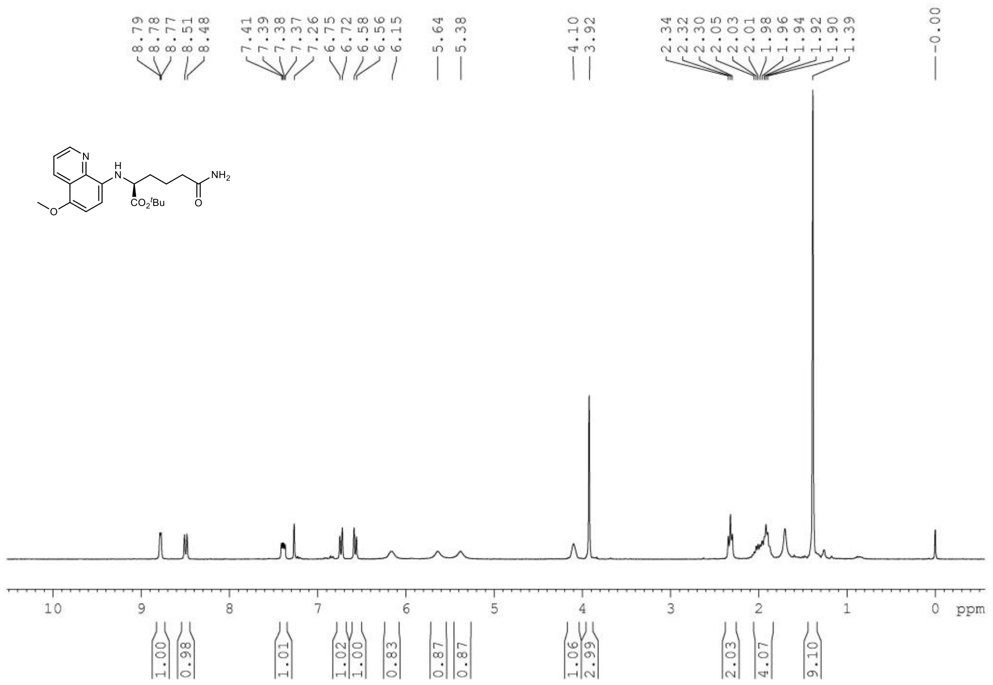

Supplementary Fig. 71. ^{1}H NMR of compound **6** (300 MHz, $CDCl_3$)

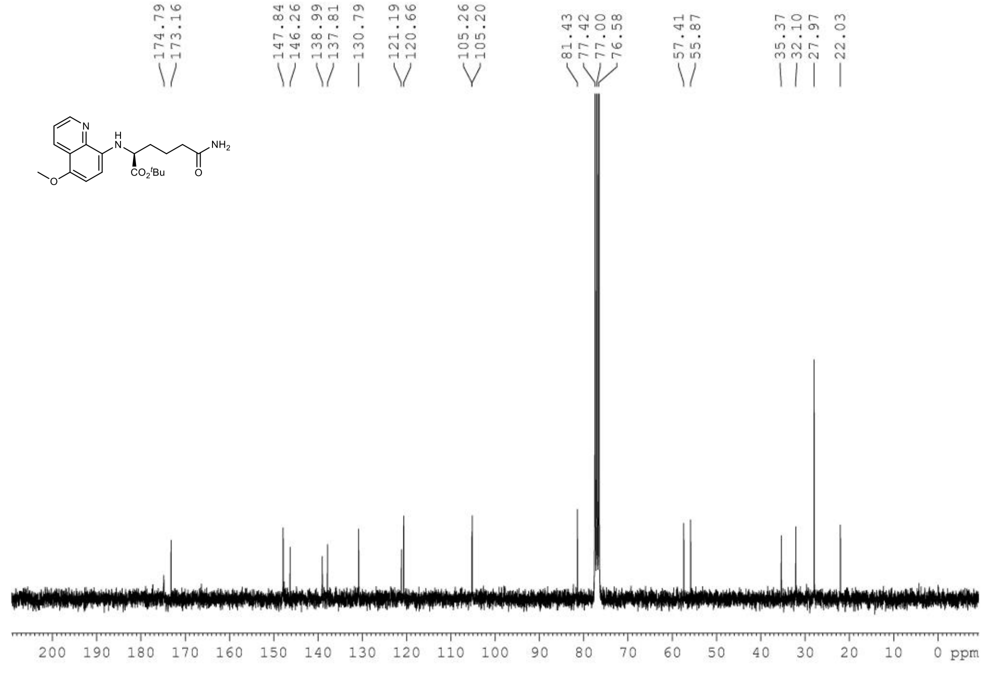

Supplementary Fig. 72. ^{13}C NMR of compound **6** (75 MHz, $CDCl_3$)

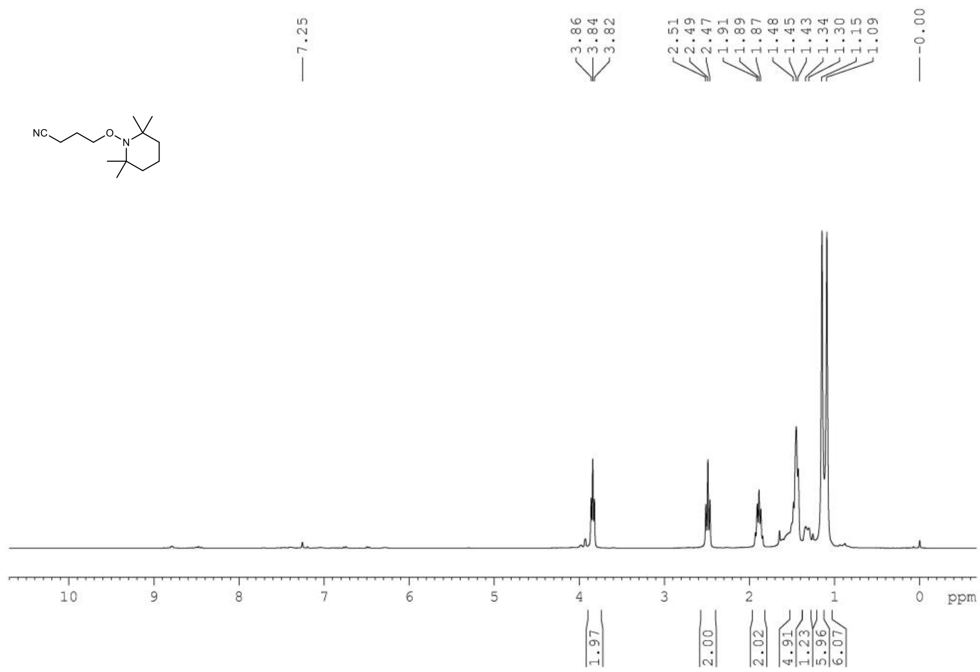

Supplementary Fig. 73. ^{1}H NMR of compound **7** (300 MHz, $CDCl_3$)

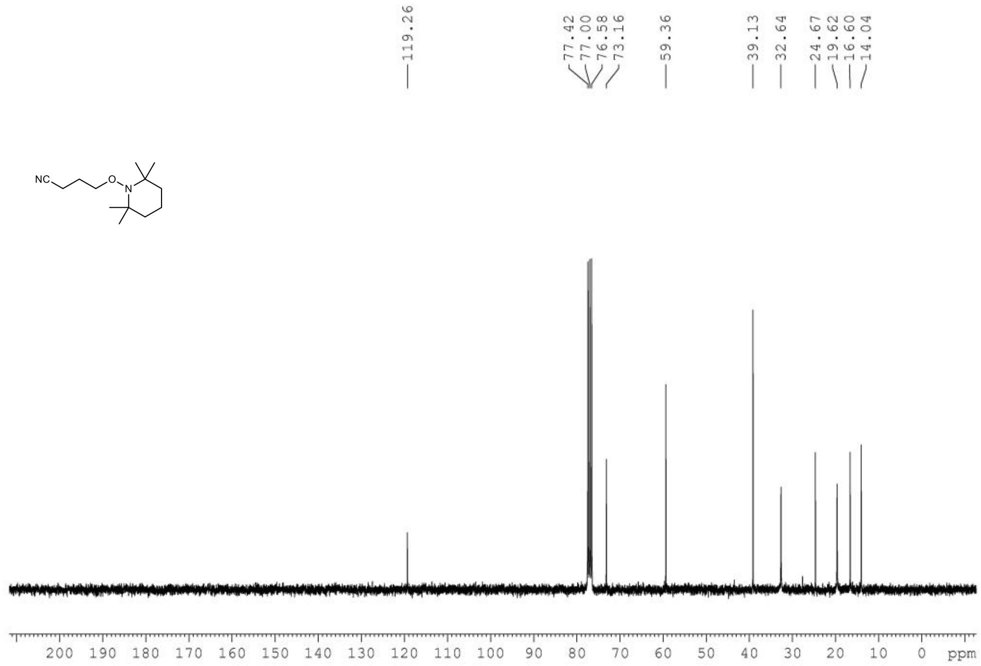

Supplementary Fig. 74. ^{13}C NMR of compound **7** (75 MHz, $CDCl_3$)

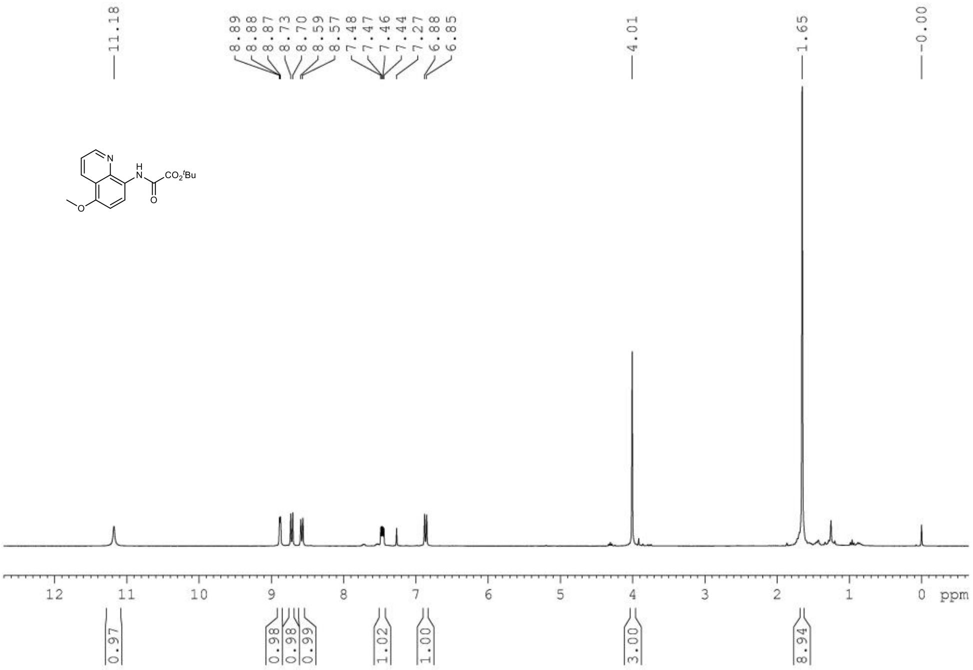

Supplementary Fig. 75. ^{1}H NMR of compound **9** (300 MHz, $CDCl_3$)

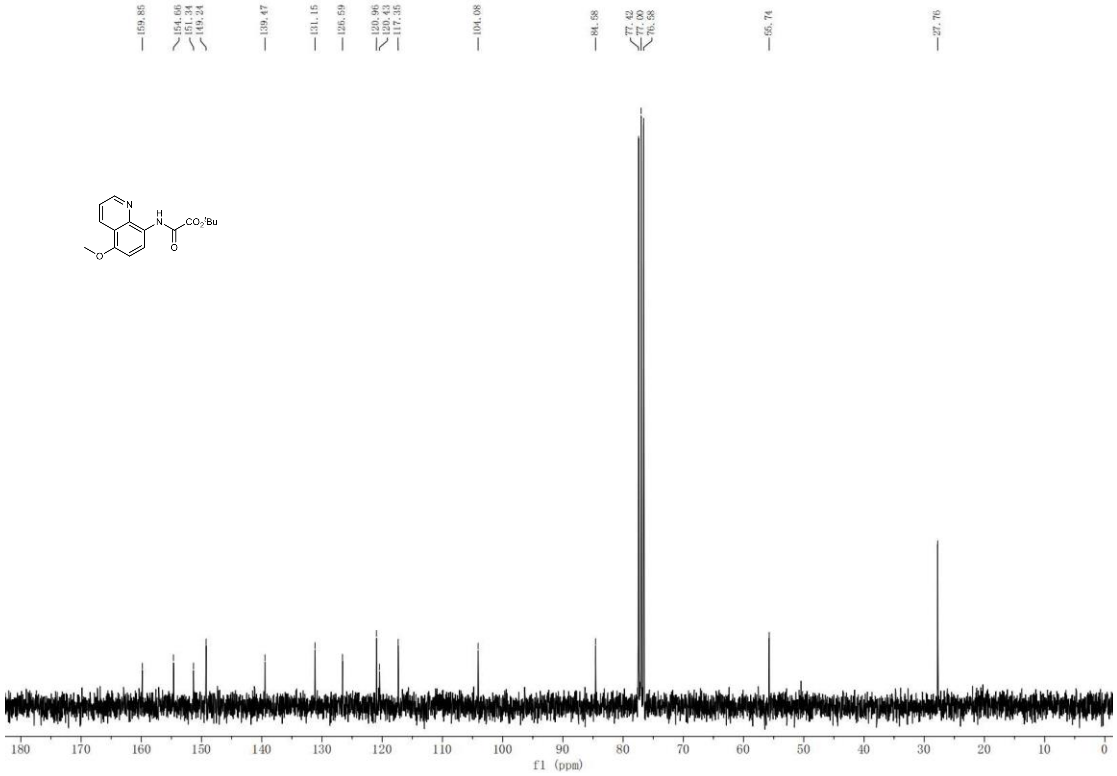

Supplementary Fig. 76. ^{13}C NMR of compound **9** (75 MHz, $CDCl_3$)

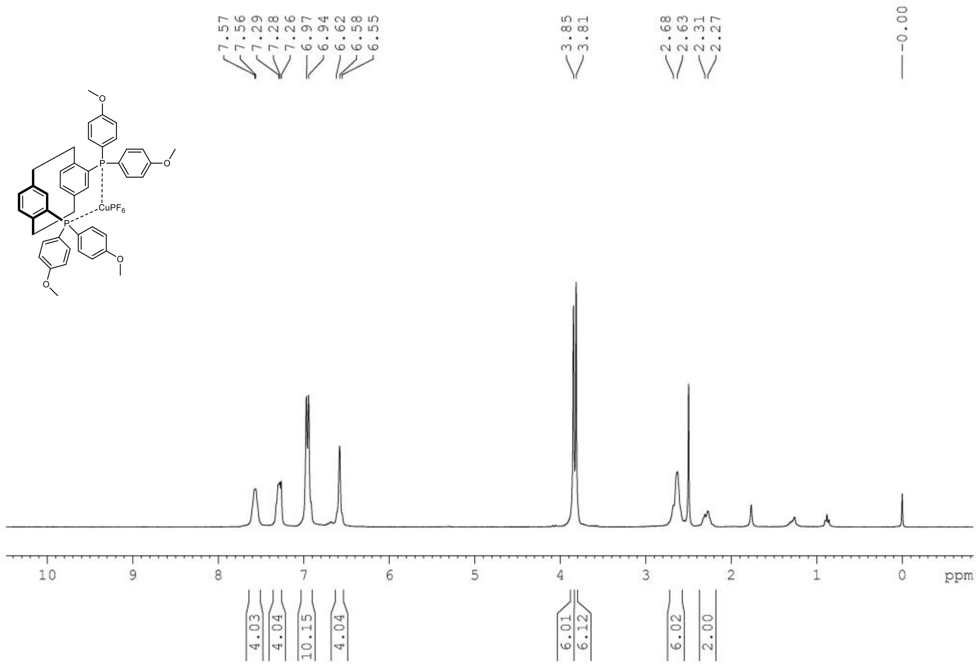

Supplementary Fig. 77. ^{1}H NMR of compound **Complex A** (300 MHz, $CDCl_3$)

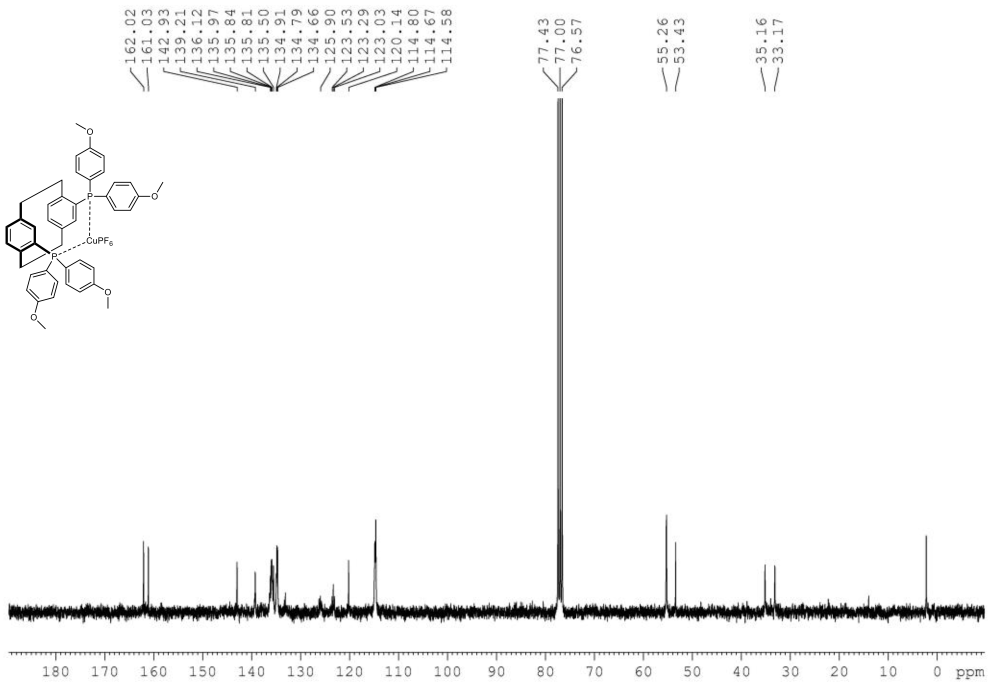

Supplementary Fig. 78. ^{13}C NMR of compound **Complex A** (75 MHz, $CDCl_3$)

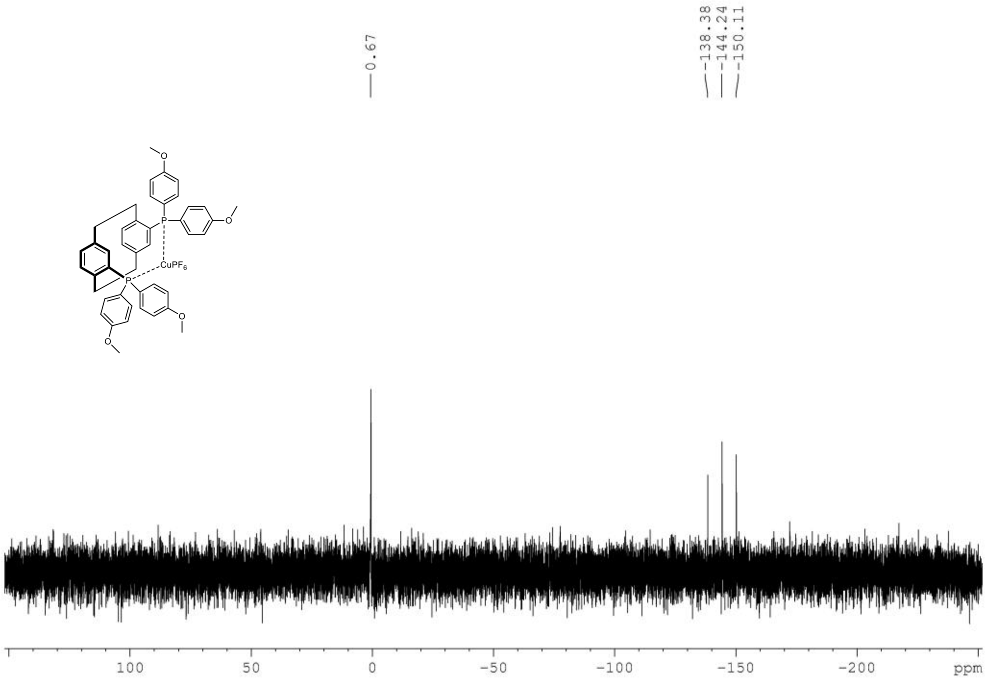

Supplementary Fig. 79. ^{31}P NMR of compound **Complex A** (121.5 MHz, $CDCl_3$)

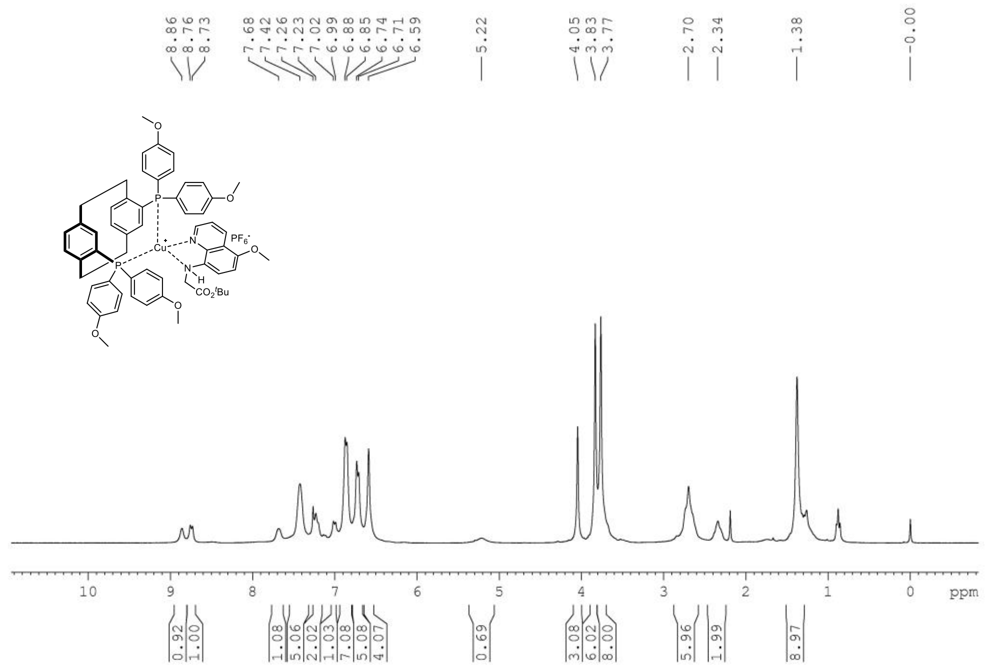

Supplementary Fig. 80. ^{1}H NMR of compound **Complex B** (300 MHz, $CDCl_3$)

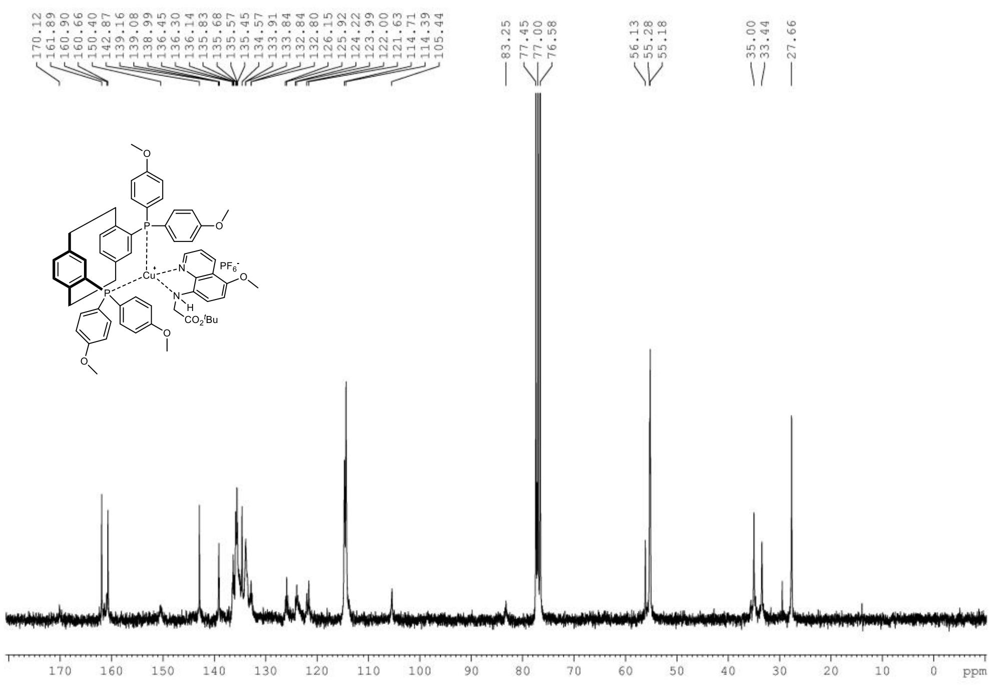

Supplementary Fig. 81. ^{13}C NMR of compound **Complex B** (75 MHz, $CDCl_3$)

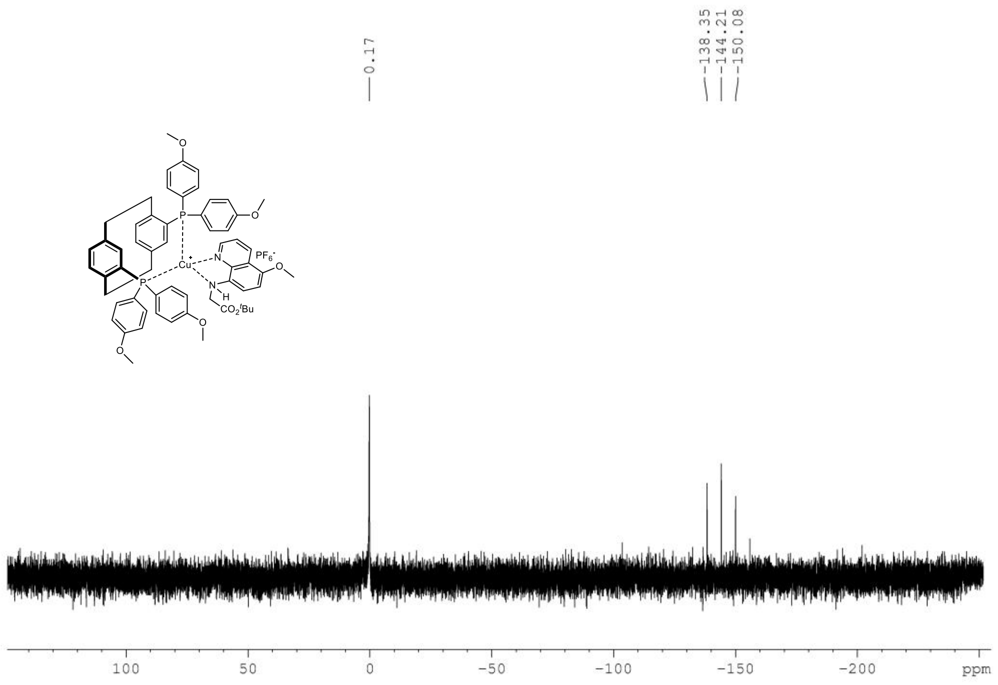

Supplementary Fig. 82. ^{31}P NMR of compound **Complex B** (121.5 MHz, $CDCl_3$)

3 Supplementary References

1. Zhi, H., Ung, S. P-M., Liu, Y., Zhao, L. & Li, C-J. Phosphorylation of glycine derivatives via copper(I)-catalyzed c(sp^3)-H bond functionalization. *Adv. Synth. Catal.* **358**, 2553-2557 (2016).
2. Wang, C. et al. Visible-light-driven, copper-catalyzed decarboxylative C(sp^3)-H alkylation of glycine and peptides. *Angew. Chem. Int. Ed.* **57**, 15841-15846 (2018).
3. Ji, P. et al. Visible-light-mediated, chemo- and stereoselective radical process for the synthesis of *C*-glycoamino acids. *Org. Lett.* **21**, 3086-3092 (2019).
4. Wang, P.-Z. et al. Asymmetric three-component olefin dicarbofunctionalization enabled by photoredox and copper dual catalysis. *Nat. Commun.* **12**, 1815-1824 (2021).
5. Chen, J. et al. Enantioselective radical ring-opening cyanation of oxime esters by dual photoredox and copper catalysis. *Org. Lett.* **21**, 9763-9768 (2019).
6. Qi, R. et al. Visible light induced Cu-catalyzed asymmetric C(sp^3)-H alkylation. *J. Am. Chem. Soc.* **143**, 12777-12783 (2021).
7. Francesca Caprioli, F., Madduri, A. V. R., Minnaard, A. J. & Harutyunyan, S. R. Asymmetric amplification in the catalytic enantioselective 1,2-addition of Grignard reagents to enones. *Chem. Commun*. **49**, 5450-5452 (2013).
8. Dolomanov, O. V., Bourhis, L. J., Gildea, R. J, Howard, J. A. K. & Puschmann, H. J. OLEX2: a complete structure solution, refinement and analysis program. *J. Appl. Cryst*. **42**, 339-341 (2009).
9. Sheldrick, G. M. SHELXT - Integrated space-group and crystal-structure determination. *Acta Cryst*. **A71**, 3-8 (2015).
10. Sheldrick, G. M. Crystal structure refinement with SHELXL. *Acta Cryst*. **C71**, 3-8 (2015).
